# CRY1–GAIP1 complex mediates blue light to hinder the repression of PIF5 on *AGL5* to promote carotenoid biosynthesis in mango fruit

**DOI:** 10.1111/pbi.70100

**Published:** 2025-04-22

**Authors:** Manman Zhang, Yongchen Fang, Fan Jiang, Yifei Liao, Chen Pan, Jiage Li, Jiahao Wu, Qinsong Yang, Rongling Qin, Songling Bai, Yuanwen Teng, Junbei Ni

**Affiliations:** ^1^ College of Agriculture and Biotechnology Zhejiang University Hangzhou Zhejiang Province People's Republic of China; ^2^ Hainan Institute of Zhejiang University Sanya Hainan Province People's Republic of China; ^3^ Key Laboratory for Silviculture and Conservation, Ministry of Education Beijing Forestry University Beijing China

**Keywords:** blue light, mango, carotenoid biosynthesis, AGL, DELLA

## Abstract

Carotenoids are essential natural pigments that not only determine the commercial value of horticultural crops through colouration but also serve as vital antioxidants and provitamin A precursors in the human diet. Our previous research has demonstrated that blue light induces carotenoid biosynthesis in mango fruit. However, a critical knowledge gap remains regarding how blue light regulates carotenoid biosynthesis in fruit. In this study, blue light‐induced *MiAGL5* was identified to promote carotenoid biosynthesis by activating the promoters of *MiBCH1* and *MiZEP*. Subsequently, MiPIF5, a phytochrome interacting factor, transcriptionally inhibited *MiAGL5* expression. MiGAIP1, a DELLA protein, promoted carotenoid biosynthesis by interacting with MiPIF5 and preventing its repression of *MiAGL5*. Furthermore, blue light stabilized MiGAIP1 protein through MiCRY1–MiGAIP1 interaction and reduced MiGAIP1 degradation by decreasing GA content in mango fruit. Additionally, MiGAIP1 mediated the antagonistic effects between blue light and GA in regulating carotenoid biosynthesis. Collectively, these results demonstrate that blue light induces carotenoid biosynthesis through a mechanism involving MiCRY1–MiGAIP1 complex‐mediated inhibition of MiPIF5 repression on MiAGL5. Our work provides solid evidence for CRY‐DELLA‐PIF‐AGL cross‐talk in plant metabolism and establishes a new paradigm for light‐hormone antagonism in the regulation of specialized metabolites.

## Introduction

Carotenoids, a group of isoprenoid metabolites (Sun and Li, 2020), play an important role in the light‐harvesting mechanism and serve as ubiquitous protective agents in photosynthetic organisms (Domonkos *et al*., 2013; Frank and Cogdell, 1985). Furthermore, carotenoids are natural pigments in plants that impart vibrant colour to fruits and act as visible signals to attract insects and animals for seed dispersal (Tanaka *et al*., 2008). In addition to their indispensable functions in plants, carotenoids are crucial for human health as bioactive compounds (Da Ribeiro Silva *et al*., 2014; Nile and Park, 2014), providing dietary antioxidants and vitamin A precursors. Mango (*Mangifera indica* L.), a tropical fruit of global importance, is rich in carotenoid compounds. Deciphering the regulatory mechanisms of carotenoid biosynthesis in mango fruit is of great importance for improving the mango fruit industry.

The enzymatic process of carotenoid biosynthesis in plants has been extensively studied (Sun and Li, 2020). The rate‐limiting step in the carotenoid biosynthetic pathway is the formation of 15‐*cis*‐phytoene by phytoene synthase (PSY). The formation of all‐*trans*‐lycopene is achieved through the continuous desaturation and isomerization of 15‐*cis*‐phytoene by phytoene desaturase (PDS), *ζ*‐carotene desaturase (ZDS), *ζ*‐carotene isomerase (Z‐ISO) and carotenoid isomerase (CRTISO). Lycopene is then cyclized by lycopene *β*‐cyclase (LCYb) to form *β*‐carotene in one branch, and by both LCYb and lycopene *ε*‐cyclase (LCYe) to form *α*‐carotene in another branch. The *α*‐carotene is hydroxylated to lutein, while the *β*‐carotene is catalysed by *β*‐carotene hydroxylase (BCH), zeaxanthin epoxidase (ZEP) and violaxanthin de‐epoxidase (VDE) (Lu and Li, 2008; Moise *et al*., 2014). The carotenoid biosynthetic pathway is regulated by multiple transcription factors (TFs) including MADS, NAC, MYB and PIF (Stanley and Yuan, 2019; Sun and Li, 2020). Classical MADS box TFs, including ripening inhibitor (RIN), AGAMOUS‐LIKE1 (TAGL1) and FRUITFULL (FUL), have been demonstrated to bind directly to the promoters of *PSY1* and a number of other carotenogenic genes, thereby accelerating carotenoid biosynthesis in tomato (Bemer *et al*., 2012; Li *et al*., 2019; Vrebalov *et al*., 2009). Recent studies have demonstrated that CsMADS3 in *Citrus* hesperidium enhances carotenoid biosynthesis by directly binding to and activating the promoters of *CsPSY1* and *CsLCYb2* (Zhu *et al*., 2023). Furthermore, the MADS TFs have been demonstrated to be transcriptionally regulated by other TFs. In tomato, the silencing of *SlARF2A/B* resulted in the formation of green spots, accompanied by a reduction in the mRNA levels of *RIN*, *TAGL1* and *FUL1/2* (Guo *et al*., 2018; Hao *et al*., 2015). However, the precise molecular mechanisms governing the transcription of MADS TFs in relation to carotenoid biosynthesis remain largely unelucidated.

Intriguingly, the activity of these transcriptional regulators, including MADS and PIFs, is not autonomous but is profoundly modulated by environmental or developmental (phytohormones) stimuli. Light, which serves as the most important environmental factor, has been reported to regulate carotenoid biosynthesis in several studies (Liu *et al*., 2015; Llorente *et al*., 2017). Plants absorb blue light through cryptochromes (CRY1 and CRY2) and red/far‐red light through phytochromes (PHYA and PHYB) (Cashmore *et al*., 1999; Smith, 2000). CRYs/PHYs inhibit the degradation of ELONGATED HYPOCOTYL 5 (HY5) (Liu *et al*., 2011; Weidler *et al*., 2012), which positively regulates fruit carotenoid accumulation by targeting the *PSY* gene (Toledo‐Ortiz *et al*., 2014; Wang *et al*., 2021). Furthermore, the CRYs/PHYs can physically interact with phytochrome interacting factors (PIFs), which belong to a subfamily of helix–loop–helix (bHLH) TFs (Franklin *et al*., 2003; Liu *et al*., 2008, 2013; Lorrain *et al*., 2008; Pedmale *et al*., 2016; Yang *et al*., 2017). PIFs negatively regulate carotenoid biosynthesis by repressing the expression of *PSY* (Leivar *et al*., 2009; Toledo‐Ortiz *et al*., 2010). Therefore, HY5 and PIFs exert an antagonistic influence on light‐regulated carotenoid biosynthesis in plants. However, the regulation of HY5 and PIFs on carotenoid biosynthesis is a common phenomenon in light signals, whereas certain monochromatic light signals may involve distinct signal transduction networks (Sharma *et al*., 2023; Tavridou *et al*., 2020; Toledo‐Ortiz *et al*., 2014). The role of monochromatic light in carotenoid biosynthesis has been the subject of several studies (Llorente *et al*., 2017). For example, blue light has been demonstrated to effectively enhance the biosynthesis and accumulation of carotenoids in peach, strawberry and mandarin fruits (Cao *et al*., 2017; Chen *et al*., 2023; Deng *et al*., 2017). CRY2 has been shown to be involved in the regulation of carotenoid biosynthesis in tomato, with overexpression of *CRY2* increasing carotenoid content (Giliberto *et al*., 2005). However, the molecular regulatory pathway from the reception of blue light by CRYs to the biosynthesis of carotenoids remains unclear.

While light provides external instructions, intrinsic phytohormones act as internal rheostats to fine‐tune carotenoid flux. Previous studies have shown that phytohormones, including ethylene (ET), abscisic acid (ABA), brassinosteroid (BR) and jasmonic acid (JA), positively regulate carotenoid metabolism in tomato fruit (Cruz *et al*., 2018; Liu *et al*., 2012, 2014; Toledo‐Ortiz *et al*., 2010). In recent years, the role of gibberellins (GA) in regulating carotenoid biosynthesis has also been investigated. GA_3_ treatment inhibits the accumulation of carotenoids and lycopene in tomato and delays the onset of colour break in citrus peel (Alós *et al*., 2006; Iglesias *et al*., 2001; Li *et al*., 2019). The GA signalling pathway has been well studied in plants. In brief, GA de‐represses its signalling pathway by binding to the GIBBERELLIN INSENSITIVE DWARF1 (GID1) receptor, inducing the ubiquitination and degradation of the master growth‐inhibiting Aspartic acid‐glutamic acid‐leucine‐leucine‐alanine (DELLA) proteins (Sun, 2011). Studies have reported that GA‐regulated DELLA proteins inhibit chlorophyll and carotenoid biosynthesis in dark‐grown etiolated cotyledons of *Arabidopsis* (Cheminant *et al*., 2011). Moreover, GA retards carotenoid content in Valencia oranges by downregulating the expression of carotenoid biosynthesis genes, including *CitPSY*, *CitPDS*, *CitZDS* and *CitLCYb* (Keawmanee *et al*., 2022). Therefore, both GA and GA‐regulated DELLA proteins are involved in the carotenoid biosynthesis process. However, the precise molecular mechanisms by which GA‐regulated DELLA proteins regulate gene expression in the carotenoid pathway remain unclear.

The convergence of environmental and hormonal signals raises a pivotal question: How do plants integrate external light cues with endogenous GA signalling to optimize carotenoid biosynthesis? In *Arabidopsis* seedlings, PIFs inhibit bud outgrowth by suppressing GA synthesis and promoting ABA synthesis (Chen *et al*., 2023). Additionally, blue light controls photomorphogenesis in *Arabidopsis* by inhibiting the degradation of DELLA proteins, thereby suppressing the GA signalling pathway (Xu *et al*., 2021). Recent studies in soybean revealed that low blue light triggers soybean leaf senescence through the GmCRY1‐GmDELLAs‐GmWRKY100 module, which integrates low blue light signals with the internal GA signalling pathway (Li *et al*., 2024). These studies indicate that the crosstalk between light and GA signals plays a crucial role in plants to regulate various biological processes. However, whether this crosstalk specifically coordinates carotenoid biosynthesis, particularly in non‐model fruit crops such as mango, remains to be investigated.

Our previous study demonstrated that blue light promotes carotenoid biosynthesis in ‘Guifei’ mango fruit, while concomitantly altering GA signalling components (Ni *et al*., 2022), leading us to hypothesize a mechanistic link between blue light and GA signalling in regulating mango carotenogenesis. In this study, we demonstrated that blue light‐activated MiCRY1 stabilizes MiGAIP1 to promote the protein interaction between MiGAIP1 and MiPIF5, which hinders the inhibitory effect of MiPIF5 on *MiAGL5*. MiAGL5 is a positive regulator of carotenoid biosynthesis by directly activating the expression of *MiBCH1* and *MiZEP*, ultimately promoting carotenoid biosynthesis in mango fruit. Additionally, blue light reduces GA levels, which decreases the degradation of MiGAIP1, further enhancing the abundance of MiGAIP1 protein. Meanwhile, we found that MiGAIP1 mediates the crosstalk between blue light and GA in antagonistically regulating carotenoid biosynthesis in mango fruit. Our results reveal a regulatory pattern of blue light‐induced carotenoid biosynthesis in mango fruit, which sheds light on the molecular mechanism of light and hormone‐coregulated carotenoid biosynthesis in plants.

## Materials and methods

### Plant materials and treatment

Approximately 100 days after full bloom, bagged ‘Guifei’ mango fruit were harvested from a commercial orchard in Sanya, Hainan Province, China. The fruit were immediately airlifted to the laboratory where the bags were removed, and the fruit stalks were trimmed. Mango fruit of uniform size and free of disease were selected, washed and placed in a climate chamber at 17 °C with 80% relative humidity in darkness overnight to eliminate field heat. The fruit were then subjected to the following treatments: blue light (453.2 nm, 110 μmol/m^2^/s), darkness, GA_3_ + blue light, GA_3_ + darkness, paclobutrazol (PAC, GA biosynthesis inhibitor) + blue light and PAC + darkness. For the GA_3_ + blue light, GA_3_ + darkness, PAC + blue light and PAC + darkness treatments, both GA_3_ and PAC (Solarbio, China) were applied by soaking. GA_3_ and PAC were dissolved in water to a concentration of 200 mg/L, and the fruit were immersed in the respective solutions for 10 min before being transferred to a climate chamber for continuous treatment with blue light or darkness. Each treatment included 162 mango fruits, ensuring that each sampling time point had three biological replicates, with each replicate consisting of 9 fruits. Fruits were randomly sampled at 0 h, 6 h, 24 h, 72 h, 144 h and 216 h after consecutive treatment. The climate chamber was maintained at a constant temperature of 17 °C and 80% relative humidity.

Fruits were sampled and physiological indicators were measured according to our previous study (Ni *et al*., 2022). Briefly, after peeling the fruit with peelers, colour parameters of the fruit flesh were determined using a handheld colorimeter (Chroma Meter CR‐400, China). Fruit firmness was measured using a texture analyser (Stable Micro Systems, UK) equipped with a 5 mm flat probe moving at a constant speed of 1 mm/s. The peeled mango fruit was placed on the platform of the analyser, and a puncture test was performed to a depth of 5 mm. The peak force value was recorded as an indicator of firmness. During the sampling process, the flesh of the 9 mango fruits in each biological replicate was quickly chopped, pooled, rapidly frozen in liquid nitrogen and stored at −80 °C until use. The stored materials were utilized for various analyses, including carotenoid and gibberellin measurements, RNA extraction, cDNA synthesis, RT‐qPCR and active protein extraction. For the treatments involving blue light exposure, samples were collected exclusively from the irradiated side of the fruit. For the dark treatment group, samples were taken randomly.

### Measurement of total carotenoid content and detection of carotenoid component

The total carotenoid content was measured as previously described (Ni *et al*., 2022). Approximately 0.1 g of the sample was used for extraction in 1.0 mL of acetone. The liquid was covered with foil and allowed to react for 50 min before centrifugation to obtain the supernatant. The absorbance of the supernatant was measured at 662, 645 and 470 nm using a spectrophotometer. Each measurement was performed with three biological replicates for biological reproducibility. The carotenoid metabolite profile of the control and transiently overexpressing *MiAGL5* (*MiAGL‐OX*) flesh of mango fruit was analysed using a liquid chromatography‐electrospray ionization‐tandem mass spectrometry system from MetWare Biotechnology Co., Ltd. (Wuhan, China; http://www.metware.cn/). The specific carotenoid metabolites are listed in Table S1.

### GA measurement

Mango fruit flesh samples at 0 h and 72 h under blue light and darkness treatments were selected for GA measurement. Three biological replicates were measured for each treatment and each time point. The fruit flesh samples stored at −80 °C were placed in liquid nitrogen and ground into powder. Subsequently, 0.5 g of sample was weighed and placed in a sample tube that was kept on dry ice to ensure sample stability. The analysis of GA content in mango fruit flesh was performed using ESI‐HPLC–MS/MS (lectrospray Ionization High‐Performance Liquid Chromatography–Tandem Mass Spectrometry).

### RNA extraction, cDNA synthesis and RT‐qPCR

Total RNA was extracted from mango fruit flesh samples of six treatment groups (darkness, blue light, GA_3_ + blue light, GA_3_ + darkness, PAC + blue light, PAC + darkness). Additionally, RNA was extracted from mango and tomato samples used in transient overexpression or silencing experiments. The extraction was performed using a modified CTAB method as described previously (Zhang *et al*., 2012). The concentration and quality of RNA were determined using a BioDrop spectrophotometer (Biochrom, Cambridge, UK) and agarose gel, respectively. The volume of 1 μg of total RNA was calculated based on the RNA concentration, and cDNA was synthesized using 1 μg of RNA according to the manufacturer's instructions (HiScript IV All‐in‐One Ultra RT SuperMix, Vazyme Biotech Co., Ltd). Each sample had three biological replicates, and the cDNA concentrations were adjusted to the same level. Real‐time PCR (RT‐qPCR) was performed using the CFX96 detection system (Bio‐Rad, Harkles, CA). The mango *MiActin* gene (*MiActin*, mango010191) was used as the housekeeping gene for data normalization. Primers used in this study were designed using online tools (https://www.ncbi.nlm.nih.gov/tools/primer‐blast/, accessed October, 2021) and are listed in Table S2.

### Sequence analysis

The phylogenetic tree was constructed using TBtool software (version 2.154) (Chen *et al*., 2020). All protein sequences were downloaded from the NCBI database (https://www.ncbi.nlm.nih.gov/, accessed September, 2022). The final visualization was performed using iTOL (https://itol.embl.de/itol.cgi, accessed October, 2022) (Letunic and Bork, 2021). Multiple protein sequence alignments were performed using DNAMAN software (version 9.0) (LynnonBioSoft, San Ramon, CA). Conserved domains were identified using tools available in the NCBI Conserved Domain Database (https://www.ncbi.nlm.nih.gov/Structure/cdd/wrpsb.cgi, accessed September, 2022). Plant‐CARE (http://bioinformatics.psb.ugent.be/webtools/plantcare/html/, accessed July, 2023) and Plant‐TFDB (https://planttfdb.gao‐lab.org/, accessed October, 2023) were utilized to analyse *cis*‐elements in the promoter regions. The protein sequences used for alignment are listed in File S1.

### Subcellular localization

The full‐length complete coding sequences (CDSs) of *MiAGL5* and *MiPIF5*, without termination codons, were cloned into the pCAMBIA1300‐GFP vector. The CDS of *MiGAIP1* was directly inserted into the pCAMBIA1205‐GFP vector to generate an N‐terminal GFP fusion protein. The plasmids were transferred into *Agrobacterium tumefaciens* GV3101 for preparation. *Agrobacterium* cells containing the vectors were infiltrated into transgenic tobacco (*Nicotiana benthamiana*) leaves, with leaf cell nuclei containing red fluorescent protein (NLS‐mCherry) as described previously. Empty vectors, pCAMBIA1300‐GFP and pCAMBIA1205‐GFP, were used as negative controls. Three days after injection, the fluorescence was observed using confocal laser scanning microscopy (A1, Nikon, Tokyo, Japan). The primer sequences used for constructing vectors are listed in Table S3.

### Trans‐activation activity assay

The full‐length CDSs of *MiAGL5* and *MiGAIP1* were inserted into the pGBKT7 (BD) vector (gene‐BD) and the VP16‐pGBKT7 vector (gene‐VP16‐BD). Gene‐BD and gene‐VP16‐BD constructs were independently transformed into yeast strain AH109 cells. VP16‐BD served as a positive control, while the BD served as a negative control. Transformed yeast cells were initially screened on synthetic defined (SD) medium lacking tryptophan (SD/−Trp), then transferred to SD medium lacking tryptophan and histidine, and supplemented with X‐*α*‐gal (SD/−Trp/−His/X‐*α*‐gal) to select for positive transformants. *β*‐galactosidase activity was measured as previously described (He *et al*., 2012). Each experiment was performed with at least three biological replicates. Primers are listed in Table S3.

### Y1H and Y2H assays

Y1H and Y2H assays were conducted using the Matchmaker Gold Yeast One‐Hybrid and Two‐Hybrid Systems Kit (Takara) according to the manufacturer's protocol. The full‐length coding sequences of *MiAGL5* and *MiGAIP1* were cloned into the pGADT7 (AD) vector to construct the prey (gene‐AD). Promoter fragments of the genes (*MiZEP*, *MiBCH1* and *MiAGL5*) were inserted into the pAbAi vector to construct the bait. The gene‐AD vectors were transformed into Y1HGold cells containing the bait, cultured in SD/−Leu medium and subsequently screened for positive yeast cells on SD/−Leu/AbA medium. Empty‐AD was used as a negative control.

In the Y2H assay, the interacting genes were cloned individually into the pGADT7 and pGBKT7 vectors, respectively, to form recombinant plasmids. Both prey and bait were then co‐transformed into Y2HGold cells. Yeast cells were plated on SD/−Leu/−Trp (DDO) medium and subsequently transferred to SD medium lacking adenine, histidine, leucine, and tryptophan, and containing AbA and X‐*α*‐gal (SD/−Ade/−His/−Leu/−Trp/X‐*α*‐gal/AbA, QDO/X‐*α*‐gal/AbA) for stringent interaction screening.

### Dual‐luciferase assay

The full‐length coding sequences of *MiAGL5*, *MiGAIP1* and *MiPIF5* were cloned into the pGreenII 0029 62‐SK vector (gene‐SK) as effectors. The promoter sequences of *MiZEP*, *MiBCH1* and *MiAGL5* were inserted into the pGreenII 0800‐*LUC* vector containing luciferase (LUC) and Renilla luciferase (REN) as reporters. The recombinant plasmids were transformed into *A. tumefaciens* strain GV3101 carrying psoup. Effectors and reporters were co‐incubated in a 10:1 ratio for 1 h prior to injection into *Nicotiana benthamiana* leaves. After 60 h, firefly luciferase and REN activities were analysed using the Dual‐Luciferase Reporter Assay System (Promega) and the GloMax96 Microplate Luminometer (Promega).

### Electrophoretic mobility shift assay

The fusion proteins MiAGL5‐GST, MiGAIP1‐GST and MiPIF5‐GST were purified from *E. coli* BL21 (DE3) cells. The 3′‐biotinylated probes were annealed in preparation. The unlabelled probe was used as a competitor probe. Electrophoretic mobility shift assay (EMSA) experiments were conducted using the LightShift Chemiluminescent EMSA kit (Thermo Fisher Scientific) according to the manufacturer's instructions. The specific sequences of the labelled and mutant probes are presented in Table S3.

### Transient transformation assay

Bagged mature mango fruit and non‐detached tomato fruit at the mature‐green stage were used as infiltration materials. In the experiment to establish a transient expression system in mango fruit flesh, the *β‐glucuronidase* (*GUS*) gene was used as a reporter gene and inserted into the pCAMBIA1301 and pGreenII 0029 62‐SK (SK) vectors, which were then transformed into the *A. tumefaciens* EHA105 and GV3101, respectively. The Agrobacterium suspension was prepared in infiltration buffer containing 100 mmol/L MES, 10 mmol/L MgCl_2_ and 200 μmol/L acetosyringone, and adjusted to OD600 = 0.8. The bacterial suspension was injected into the mango flesh, which was then transferred to a greenhouse at a temperature of 17 °C for co‐incubation. The fruits were kept in the dark for 1 day and then exposed to white light at an intensity of 110 μmol/m^2^/s. Tissue samples were collected at 5, 7 and 9 days after infiltration for the GUS staining analysis. GUS reporter activity was detected in most of the flesh samples at 7 and 9 days, with the exception of flesh infiltrated with GV3101 carrying the SK‐GUS vector. Given that GUS activity was higher in the mango flesh infiltrated with EHA105 containing the pCAMBIA1301‐GUS vector, and considering the convenience of tissue sampling at 7 days after infiltration, *A. tumefaciens* EHA105 harbouring the pCAMBIA1301‐GUS vector at 7 days was selected as the optimal approach to verify mango gene function.

For transient overexpression experiments, the full‐length coding sequences of *MiZEP*, *MiAGL5*, *MiGAIP1* and *MiPIF5* were independently ligated into the pCAMBIA1301 vector containing the *35S* promoter (gene‐1301). The recombinant plasmids were introduced into *A. tumefaciens* EHA105 cells and cultured. The suspension was injected into the flesh of mango and tomato fruit using a sterile syringe. The infiltrated fruits were then incubated in darkness for a period of time and then transferred to continuous white light (110 μmol/m^2^/s). The empty vector was used as a negative control. All fruits were collected 7 days after infiltration.

Virus‐induced gene silencing (VIGS) experiments were performed specifically on mango fruit. In brief, specific sequence segments of about 200–300 bp within the CDS of the target gene were cloned into the vector pTRV2 (gene‐pTRV2). The recombinant plasmids were transformed into *A. tumefaciens* GV3101 cells. Mango flesh was transiently injected with a 1:1 mixture (gene‐TRV) of gene‐pTRV2 and pTRV1. A mixture of pTRV2 and pTRV1 was co‐infiltrated into the mango flesh as a negative control. The suspension was injected into the upper left and lower right sections of the same surface of the mango fruit, respectively. After a period of incubation in darkness, the fruit were transferred to continuous white light (110 μmol/m^2^/s). For all transient transformation experiments, three independent transformation events were performed using separate batches of mango fruit. Each transformation was carried out with 50 mango fruits per biological replicate to ensure the reliability of the results. During the sampling process, the flesh from the 50 fruits in each biological replicate was pooled and stored for carotenoid measurement, RNA extraction, cDNA synthesis and RT‐qPCR.

### Bimolecular fluorescence complementation assay

The recombinant vectors containing full‐length coding sequences of *MiGAIP1*, *MiPIF5* and *MiCRY1* in p2YN and p2YC vectors were transformed into *A. tumefaciens* strain GV3101 cells. Equal volumes of different mixtures were combined for infiltration into the leaves of transgenic lines carrying red fluorescent protein. The combinations of gene‐2YN + 2YC and 2YN + gene‐2YC were used as negative controls. After co‐culturing for 60 h, followed by a pre‐dark treatment for 4 h, the fluorescence of YFP was observed using confocal laser scanning microscopy (A1, Nikon, Tokyo, Japan).

### Luciferase complementation experiment

The full‐length coding sequences of *MiGAIP1*, *MiPIF5* and *MiCRY1* were cloned into the nLUC and cLUC vectors. The recombinant plasmids were then transformed into *A. tumefaciens* strain GV3101 cells and expressed in *Nicotiana benthamiana* leaves. After 60 h of co‐cultivation, 0.5 mM luciferin (Promega) was injected near the injection site. LUC‐generated chemiluminescence was observed using the NightOWL II LB983 low‐light cooled CCD imaging system.

### Co‐IP assay

The CDS of the potential interactive proteins were inserted into the pCAMBIA1300‐GFP (gene‐GFP) and pCAMBIA1301‐Myc (gene‐Myc) vectors, respectively. *A. tumefaciens* GV3101 cells containing gene‐GFP and gene‐Myc were co‐infiltrated into *Nicotiana benthamiana* leaves. Empty‐GFP and gene‐Myc were co‐infiltrated as negative controls. Whole leaf protein was then extracted from the leaves. The protein was incubated with anti‐GFP magnetic beads for immunoprecipitation of either gene‐GFP or GFP. The immunoprecipitated complexes were subjected to immunoblot analysis using anti‐Myc antibodies.

### GST pull‐down assay

The CDS of the potentially interactive proteins were ligated separately into the pGEX4T and pET32a vectors, and transformed into *E. coli* BL21 (DE3) cells for the expression of fusion proteins (gene‐His and gene‐GST). The GST protein served as a negative control. Equal volumes of gene‐His and gene‐GST proteins were mixed for 2 h and then incubated with glutathione‐Sepharose beads at 4 °C overnight. GST protein was eluted on a column using elution buffer (GST). In Western blot analysis, anti‐His and anti‐GST antibodies were used for detection, with anti‐His antibodies showing the pull‐down assay results.

### Protein degradation assay

The full‐length coding sequence of *MiGAIP1* was inserted into the pGEX4T vector and transformed into *E. coli* BL21 (DE3) cells for the expression of the fusion protein (MiGAIP1‐GST). The full‐length coding sequence of *MiCRY1* was cloned into the pCAMBIA1300‐GFP vector (MiCRY1‐GFP). The MiCRY1‐GFP plasmid was transformed into *A. tumefaciens* GV3101 cells and injected into leaves of *Nicotiana benthamiana* for expression. The MiCRY1‐GFP‐overexpressing leaf protein, extracted after 60 h of injection, was evenly dispersed into reaction tubes containing MiGAIP1‐GST and incubated at 37 °C for a specified time. MiGAIP1‐GST was added to reaction tubes containing GFP leaf extract as a negative control.

For the *in vivo* MiGAIP1 accumulation assay, the coding sequence of *MiGAIP1* was fused with a Myc tag and inserted into the pCAMBIA1301 vector, which was then introduced into *A. tumefaciens* GV3101. MiGAIP1‐Myc and MiCRY1‐GFP or GFP were mixed in a 1:1 ratio and co‐injected into mango fruit flesh or *Nicotiana benthamiana* leaves for overexpression. Samples were collected 3 days after injection. Total protein was isolated and immunoblot analysis was performed using anti‐GFP and anti‐Myc antibodies.

### Preparation of MiGAIP1‐specific antibody and extraction of active protein from mango fruit flesh

The fusion protein of MiGAIP1‐GST was purified *in vitro*. The antibody obtained is an affinity‐purified anti‐MiGAIP1 polyclonal antibody, prepared by Beijing Wanyu Meilan Technology Co., Ltd. Total protein was extracted from 1 g of mango flesh by immersion in 5 mL of acetone for 1 h. After centrifugation, the precipitate was obtained. This procedure was repeated three times. Then, 2.5 mL of 1×PBS (Phosphate Buffered Saline) buffer was added to the precipitate and extracted for 2 h. After centrifugation, the supernatant containing the active protein from mango fruit flesh was obtained.

### Statistical analysis

Student's *t*‐test and a one‐way ANOVA (Tukey) were performed using GraphPad Prism software (version 8.0). For *t*‐test, asterisks indicate significantly different values (*P < 0.05, **P < 0.01). For one‐way ANOVA (Tukey) analysis, different lowercase letters above the error bars indicate significant differences (*P* < 0.05). To control for false positive rates due to multiple comparisons, we applied the Benjamini–Hochberg false discovery rate (FDR) correction method to adjust *P*‐values, with a significance threshold set at *q* < 0.05.

## Results

### Blue light promotes carotenoid biosynthesis and mango fruit flesh colouration

**Figure 1:**
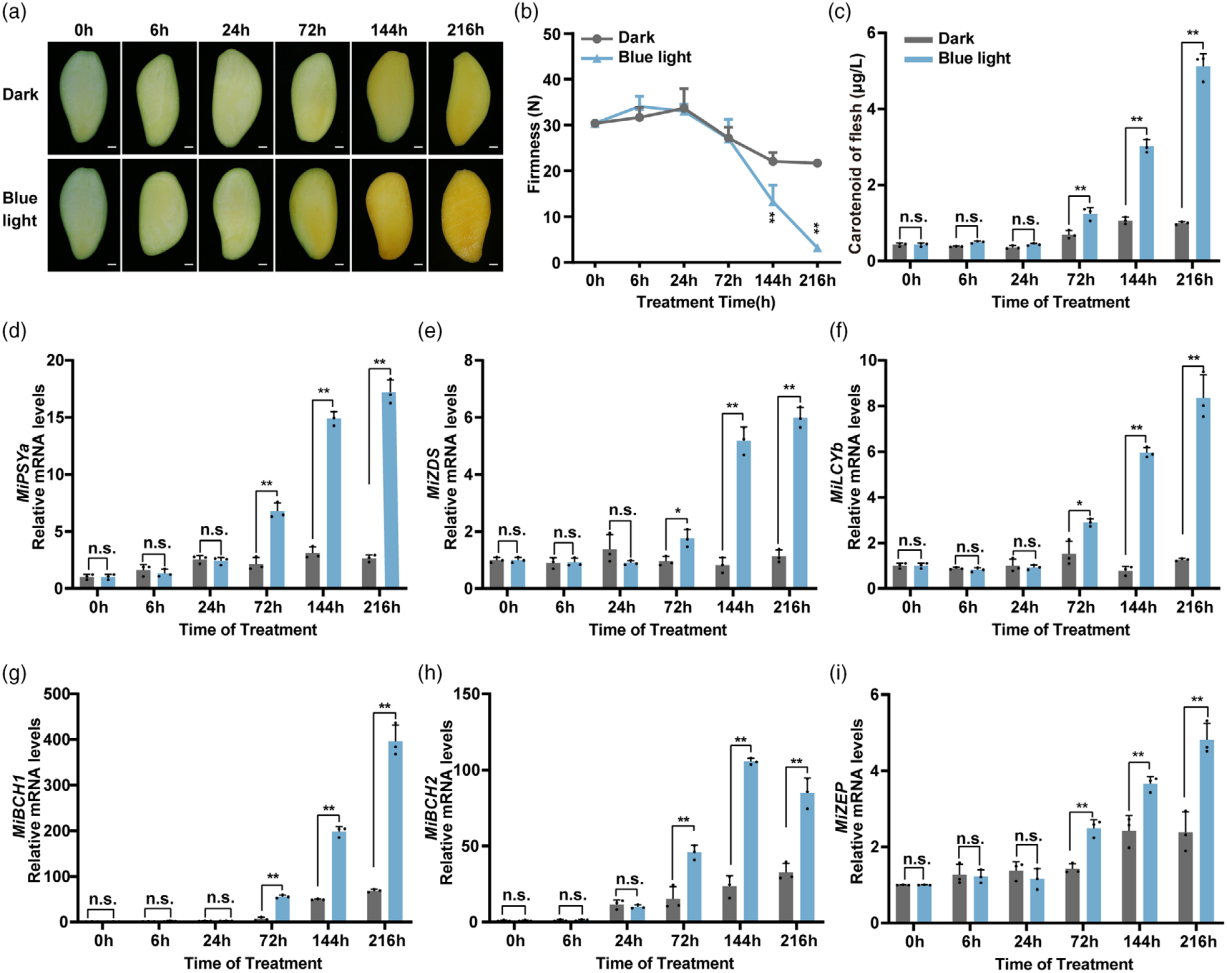
Blue light treatment promotes carotenoid biosynthesis and flesh colouration in ‘Guifei’ mango fruit. (a) Images of the flesh of ‘Guifei’ mango fruit treated with blue light and darkness (control). Scale bars, 1 cm. (b) Effects of blue light on the firmness of mango fruit flesh. (c) Effects of blue light on the carotenoid content in mango fruit flesh. (d–i) Relative mRNA levels of *MiPSYa*, *MiZDS*, *MiLCYb*, *MiBCH1*, *MiBCH2* and *MiZEP*. Error bars represent the standard deviation of three biological replicates. * indicates statistically significant differences (*P < 0.05 and **P < 0.01) as determined by a Student's *t‐*test. n.s., no significant difference.

In our previous study, we found that blue light substantially promoted carotenoid biosynthesis in the flesh of mango fruit (Ni *et al*., 2022). In this study, we further validated this finding with repeated experiments. The results showed that blue light treatment facilitated the yellowing of mango fruit flesh and rapidly reduced the firmness of the flesh while substantially inducing the biosynthesis of carotenoids (Figure 1a–c). Expression analysis demonstrated that the expression levels of structural genes in the carotenoid biosynthesis pathway (*MiPSYa*, *MiZDS*, *MiLCYb*, *MiBCH1*, *MiBCH2* and *MiZEP*) were significantly upregulated under blue light compared with the control (kept in darkness) (Figure 1d–i).

**Figure 2:**
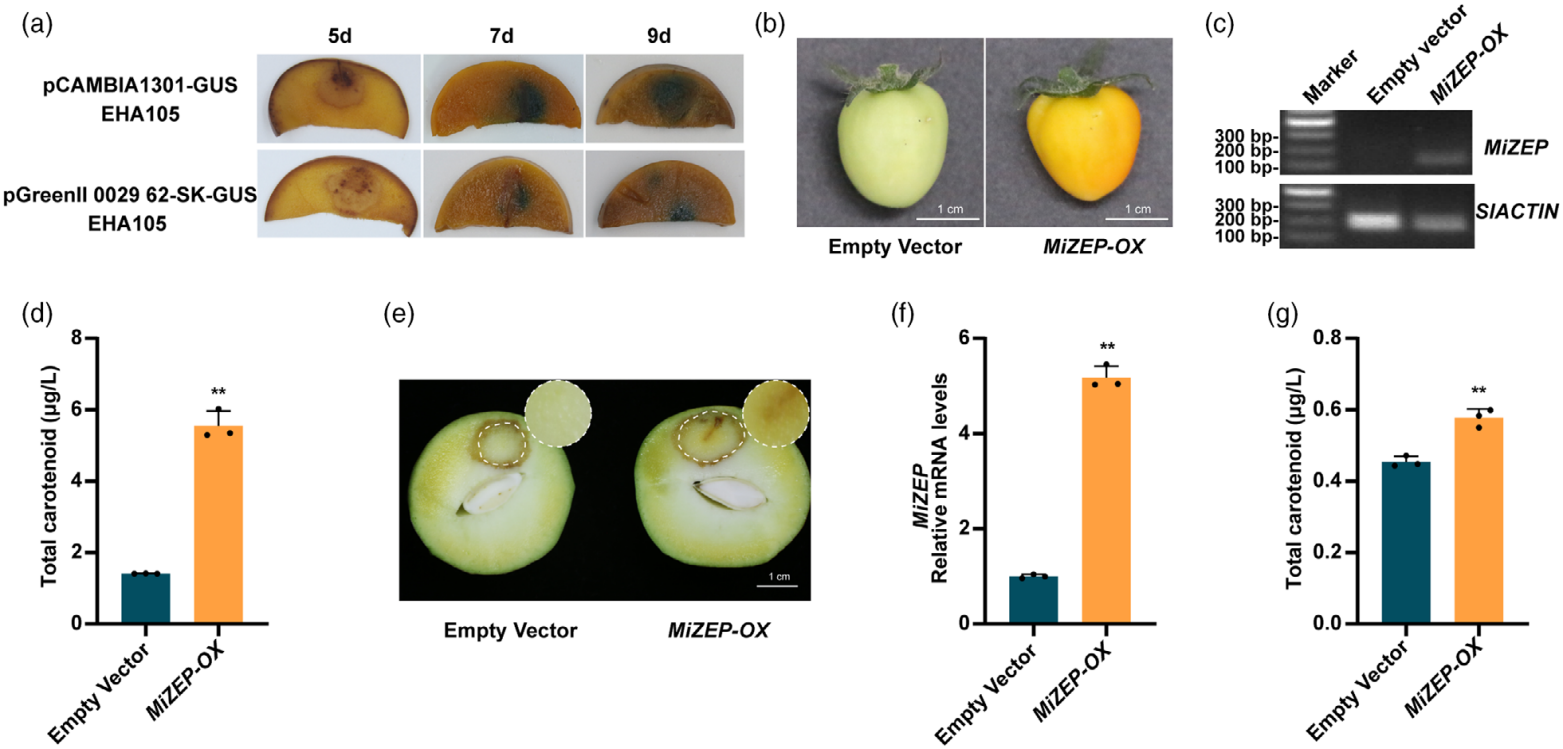
Establishment of a transient overexpression system in mango flesh and functional analysis of MiZEP in ‘MicroTom’ tomato fruit and ‘Guifei’ mango flesh. (a) Representative images of mango flesh after GUS staining following transient expression of the *GUS* reporter gene. (b) Transient overexpression of *MiZEP* (*MiZEP‐OX*) in tomato fruit. Fruits infiltrated with an empty pCAMBIA1301 vector served as the control. The fruits were harvested 7 days after infiltration. (c) Results of the RT‐PCR amplification of the *MiZEP*‐specific fragments in ‘MicroTom’ tomato fruit. (d) Total carotenoid content in tomato fruit transiently overexpressing *MiZEP*. (e) Phenotypes of *MiZEP*‐overexpression (*MiZEP‐OX*) mango flesh around the injection site, with the empty vector as a negative control. (f) The expression levels of *MiZEP* in *MiZEP‐*overexpressing mango flesh. (g) Total carotenoid content in mango flesh with transient overexpression of *MiZEP*. Phenotypes were examined after 2 days of dark and 5 days of white light treatment. Error bars represent the standard deviation of three biological replicates. Scale bars, 1 cm. * indicates statistically significant differences (*P < 0.05 and **P < 0.01) as determined by a Student's *t‐*test.

Analysis of carotenoid compounds in mango flesh revealed that violaxanthin and its derivatives were the most abundant carotenoids in the blue light‐treated mango fruit flesh, which might be due to the increased expression level of *MiZEP* (de Lucas *et al*., 2008). To better elucidate the function of the structural gene *MiZEP* in the carotenoid biosynthetic pathway, we conducted transient overexpression analysis using green‐mature stage tomato fruit and ‘Guifei’ mango fruit. GUS staining assay of mango flesh showed that higher GUS reporter activity in the mango flesh infiltrated with EHA105 containing the pCAMBIA1301‐GUS vector (Figures 2a and S1). In tomato fruit, *MiZEP* overexpression (*MiZEP‐OX*) resulted in a noticeably more intense yellow colour compared to the control, along with a significant increase in total carotenoid content (Figures 2b–d and S2a–c). In mango fruit, we observed that the flesh colour near the injection site became yellow in mango flesh overexpressing *MiZEP*, while it remained pale white in the control (Figure 2e). The expression levels of *MiZEP* in the *MiZEP‐OX* flesh were significantly increased compared to the control, indicating successful overexpression of *MiZEP* (Figure 2f). The total carotenoid content in the *MiZEP‐OX* fruit flesh was significantly higher than that in the control samples (Figure 2g). Taken together, these results demonstrated that the structural gene *MiZEP* in the carotenoid biosynthetic pathway positively participates in carotenoid biosynthesis, thereby promoting the yellow colouration in mango flesh.

### Identification of the blue light‐induced MiAGL5 and its transcriptional activation on *MiZEP*

**Figure 3:**
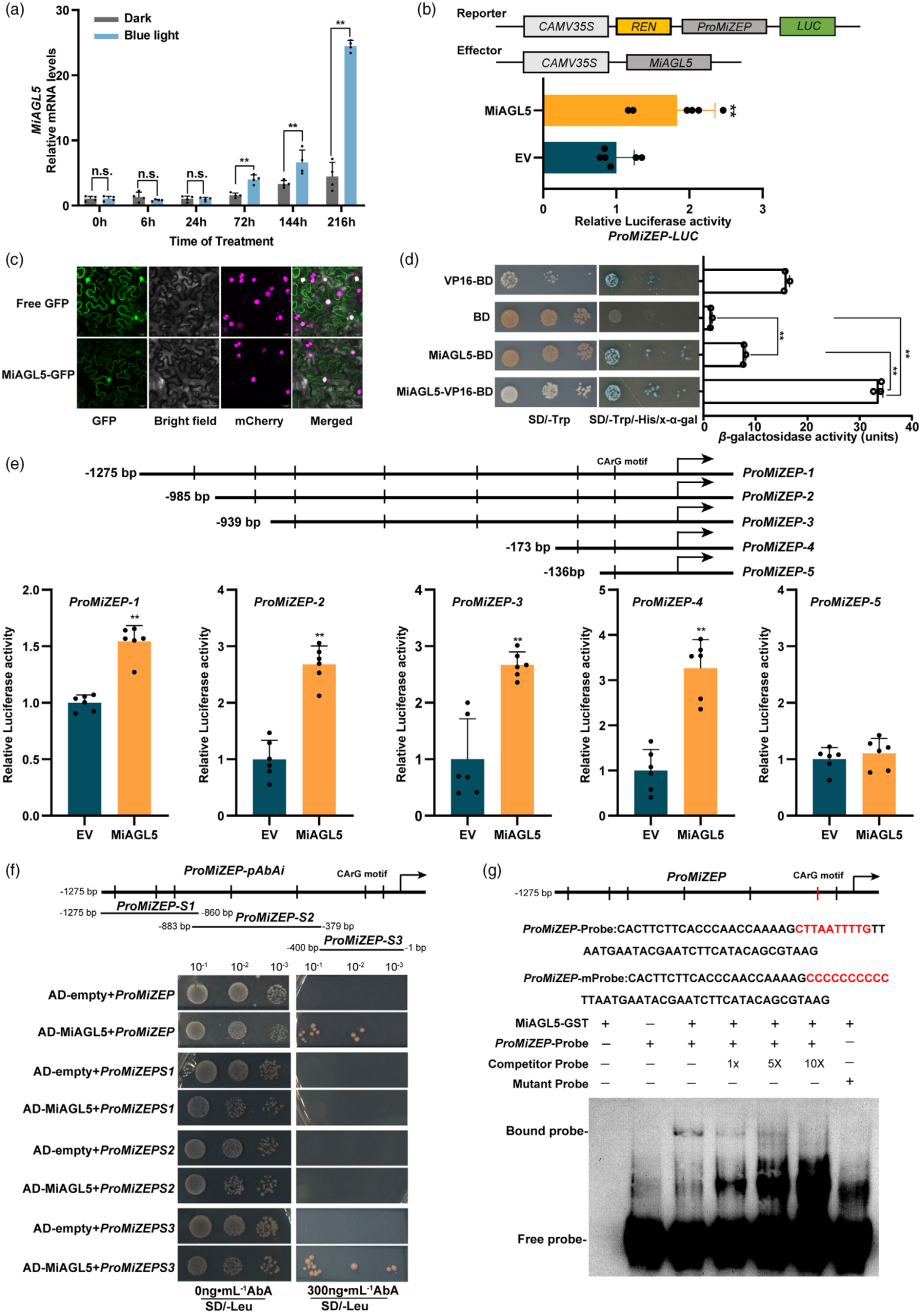
Blue light‐induced MiAGL5 activates *MiZEP* transcription. (a) The expression patterns of *MiAGL5* in mango flesh after blue light treatment. *MiActin* served as an internal reference. (b) Dual‐luciferase assay demonstrating the relative MiAGL5 activation to the promoter of *MiZEP*. The full‐length CDS of *MiAGL5* was cloned into the pGreenII 0029 62‐SK vector as the effector and the *MiZEP* promoter was cloned into the pGreenII 0800‐*LUC* reporter vector as the reporter. The empty SK vector was used as the negative control (EV). The recombinant plasmids were co‐infiltrated into *Nicotiana benthamiana* leaves to analyse the relative LUC activity. (c) Subcellular localization of MiAGL5. The MiAGL5‐GFP fusion vector and GFP were transiently transformed into *Nicotiana benthamiana* leaves, and the localization was observed by confocal laser scanning microscopy. (d) Transcriptional analysis of MiAGL5 using a yeast assay, with *β*‐galactosidase activities reflecting transcriptional activation by MiAGL5. (e) Dual‐luciferase assay showing that MiAGL5 activates the promoter of *MiZEP in vivo*. The promoter of *MiZEP* was segmented and named *ProMiZEP‐1*, *ProMiZEP‐2*, *ProMiZEP‐3*, *ProMiZEP‐4* and *ProMiZEP‐5*. These fragments were cloned into the pGreenII 0800‐*LUC* vector, and the full‐length CDS of *MiAGL5* was inserted into the SK vector. The empty vector of SK was used as a control. Relative luciferase activity was analysed. (f) Yeast one‐hybrid (Y1H) assay indicating that MiAGL5 binds to the truncated *MiZEP* promoter fragments containing the GArG sequence. The promoter of *MiZEP* was segmented and named *ProMiZEPS1*, *ProMiZEPS2* and *ProMiZEPS3*. AD‐empty (pGADT7) served as a negative control. (g) Electrophoretic mobility shift assay (EMSA) assay confirming the binding of MiAGL5 protein to the GArG motif on the promoter of *MiZEP*. An EMSA was performed by using a biotin‐labelled *MiZEP* promoter fragment containing the GArG motif (CTTAATTTTG), while the competitor probe was an unlabelled probe (1‐, 5‐ and 10‐fold molar excess). The mutant probe was identical to the labelled probe, but the CTTAATTTTG was mutated to CCCCCCCCCC. The MiAGL5‐GST used for the EMSA was purified. Error bars represent the standard deviation of three biological replicates. * indicates significantly different values (*P < 0.05 and **P < 0.01) as determined by a Student's *t‐*test.

To identify the potential regulatory mechanism underlying the blue light‐induced carotenoid biosynthesis in mango flesh, transcriptomic analysis identified five candidate TFs, including MiAGL5, MiERF1, MiERF61L, MiMYB70 and MiMUTE (Figure S3) (Ni *et al*., 2022). These candidate TFs were selected for further investigation to elucidate the mechanisms underlying blue light‐regulated carotenoid biosynthesis in mango flesh. Considering that violaxanthin is the carotenoid with the highest content in blue light‐treated mango fruit flesh (Ni *et al*., 2022), and the expression pattern of *MiZEP*, which encodes a key enzyme for violaxanthin biosynthesis, was highly positively correlated with these candidate genes (Figures 3a and S4), this suggests that these genes might regulate the expression of *MiZEP* to affect carotenoid biosynthesis in mango flesh.

The dual‐luciferase assay demonstrated that only MiAGL5 effectively activated the *MiZEP* promoter, while the other four candidate TFs failed to achieve this effect (Figures 3b and S5). Therefore, MiAGL5 was selected for further investigation. Protein sequence analysis and alignment revealed that MiAGL5 contained conserved MADS and K‐box domains (Figure S6). Subcellular localization and transcriptional activity assays revealed that MiAGL5 is a nuclear‐localized transcriptional activator (Figure 3c,d).

To further analyse the transcriptional effect of MiAGL5 on *MiZEP*, we analysed the promoter of *MiZEP* and detected 7 GArG motifs (i.e., the binding sites of MADS TFs) (Figure 3e). Therefore, we hypothesized that MiAGL5 might directly bind to the promoter of *MiZEP* and activate its expression. To validate this hypothesis, we conducted dual‐luciferase assays on segmented fragments of the *MiZEP* promoter by systematically truncating it based on the GArG motifs. The results showed that MiAGL5 activated the relative LUC activity in *ProMiZEP‐1*, *ProMiZEP‐2*, *ProMiZEP‐3* and *ProMiZEP‐4*, but not in *ProMiZEP‐5*, suggesting that the GArG motif located between −173 bp and −136 bp may serve as the binding site for MiAGL5 (Figure 3e). Subsequently, Y1H assays showed that MiAGL5 bound to the promoter of *MiZEP* and *MiZEP‐S3* (Figure 3f), indicating that MiAGL5 binds to the S3 fragment (−1 bp to −400 bp) of the promoter of *MiZEP* in the yeast system. An electrophoretic mobility shift assay (EMSA) further verified that MiAGL5 specifically binds to the CArG motif (−164 bp to −154 bp) of the *MiZEP* promoter (Figure 3g). Taken together, these results suggest that MiAGL5 promotes *MiZEP* transcription by binding to the GArG motif on the promoter.

### MiAGL5 promotes carotenoid biosynthesis by inducing the expression of carotenoid biosynthetic genes

**Figure 4:**
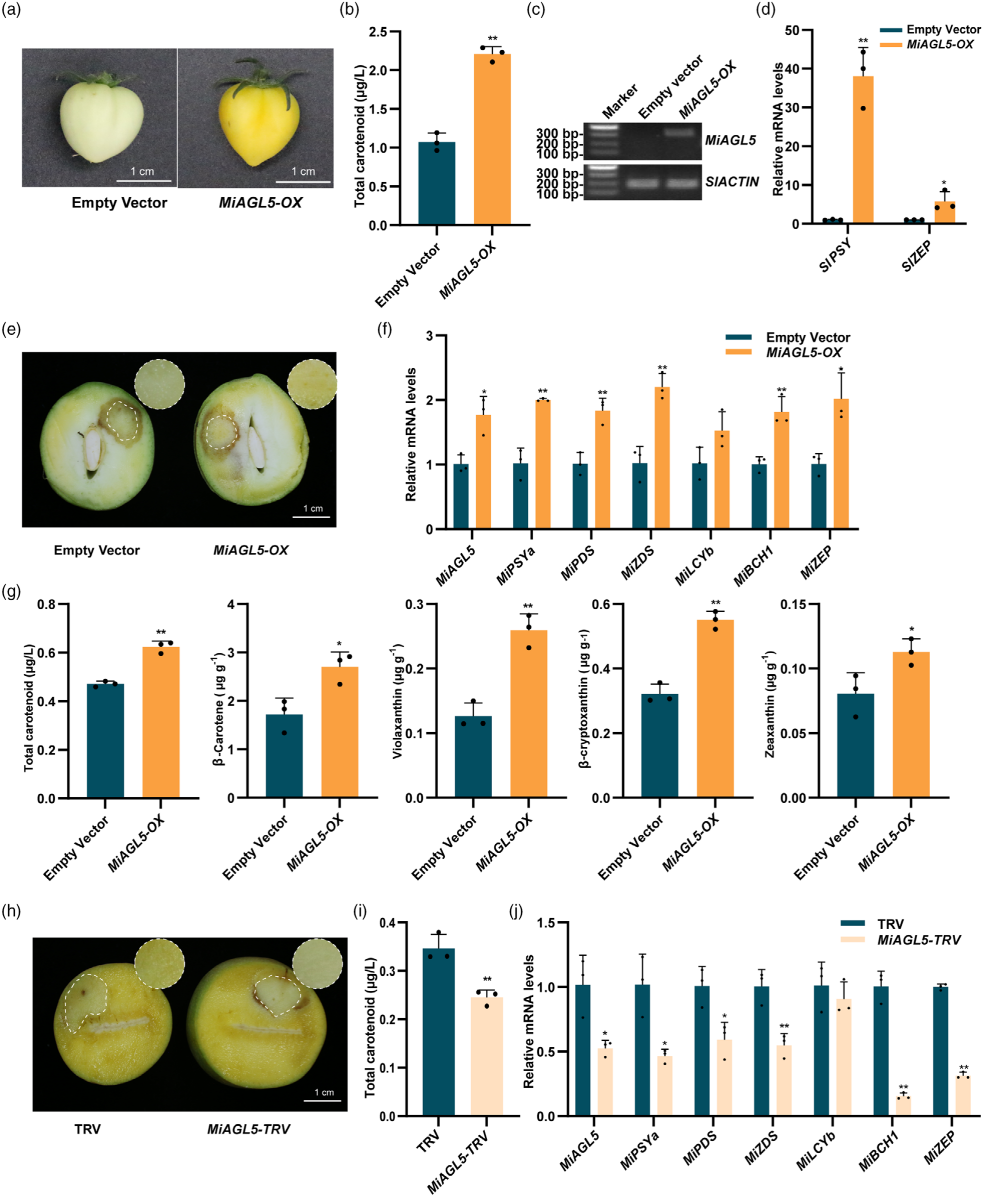
Functional analysis of MiAGL5 in ‘MicroTom’ tomato fruit and ‘Guifei’ mango fruit. (a) Transient overexpression of *MiAGL5* (*MiAGL5‐OX*) in on‐tree tomato fruit. Tomato fruit were infiltrated with *A. tumefaciens* EHA105 cells containing the recombinant pCAMBIA1301‐*MiAGL5* plasmid at the green‐mature stage. Empty vector was used as a negative control. Fruits were harvested 7 days after infiltration. (b) Total carotenoid content in tomato fruit transiently overexpressing *MiAGL5*. (c) Results of the RT‐PCR amplification of the *MiAGL5*‐specific fragments in ‘MicroTom’ tomato fruit. (d) The expression levels of carotenoid biosynthetic genes in *MiAGL5*‐overexpressing tomato fruits. (e) Phenotypes of *MiAGL5*‐overexpression (*MiAGL5‐OX*) mango fruit flesh around the injection site. Empty vector was used as the negative control. The injected mango fruit were placed in the dark for 2 days, then transferred to white light (110 μmol/m^2^/s) for co‐cultivation for 5 days. The flesh from the injection sites was collected. (f) Expression patterns of carotenoid biosynthesis‐related genes in fruit flesh transiently overexpressing *MiAGL5*. (g) Carotenoid content and compounds in *MiAGL5*‐overexpressing mango fruit flesh. (h) Transient silencing of *MiAGL5* in fruit flesh. The empty vector (pTRV1 + pTRV2) was used as negative control. (i) The total carotenoid content in the fruit flesh in which *MiAGL5* was transiently silenced. (j) The expression patterns of carotenoid biosynthesis‐related genes in the fruit flesh in which *MiAGL5* was transiently silenced. Bar = 1 cm. Error bars represent the standard deviation of 3 biological replicates. * indicates statistically significant differences (*P < 0.05 and **P < 0.01) as determined by a Student's *t‐*test.

To determine whether MiAGL5 promotes carotenoid biosynthesis, we performed a transient overexpression analysis in ‘MicroTom’ tomato fruit. We observed that tomato fruit overexpressing MiAGL5 (*MiAGL5‐OX*) turned yellow, while the control fruit remained pale‐green, which was supported by a significantly higher total carotenoid content in *MiAGL5‐OX* compared to the control (Figure 4a–c). Moreover, the expression of structural genes of the carotenoid biosynthesis pathway, *SlPSY* and *SlZEP*, was significantly upregulated in the *MiAGL5‐OX* tomato fruit (Figure 4d).

To further verify the function of MiAGL5 in regulating carotenoid biosynthesis in mango fruit, we performed transient overexpression analysis and VIGS analysis using bagged ‘Guifei’ mango fruit. The results showed that the flesh overexpressing *MiAGL5* (*MiAGL5‐OX*) turned yellow, while the control fruit flesh remained white (Figure 4e). The expression levels of *MiPSYa*, *MiPDS*, *MiZDS*, *MiBCH1* and *MiZEP* were significantly higher in the *MiAGL5‐OX* fruit flesh than in the control (Figure 4f). Total carotenoid content was considerably increased in the *MiAGL5‐OX* mango fruit flesh (Figure 4g). LC–MS/MS analysis showed that *MiAGL5* overexpression significantly increased the contents of *β*‐carotene, violaxanthin, *β*‐cryptoxanthin and zeaxanthin compared to the control (Table S1, Figure 4g). Conversely, silencing *MiAGL5* suppressed carotenoid biosynthesis and displayed slightly lighter yellow colouration in mango fruit flesh around the injection site (Figures 4h,i and S7). Silencing of *MiAGL5* significantly reduced the expression of the carotenoid biosynthetic genes *MiPSYa*, *MiPDS*, *MiZDS*, *MiBCH1* and *MiZEP* (Figure 4j). These results indicated that MiAGL5 promotes the colouration of mango fruit flesh by inducing the expression of carotenoid biosynthesis genes.

### MiAGL5 directly binds to the promoter of *MiBCH1* and activates its expression

Transient overexpression and VIGS of *MiAGL5* affected the expression of certain carotenoid biosynthetic genes besides *MiZEP*, suggesting that these genes might also be targets of MiAGL5. We then screened for potential *cis*‐elements in the promoters of these genes and detected four GArG motifs in the *MiBCH1* promoter. Yeast one‐hybrid assay results demonstrated that MiAGL5 binds to the promoter of *MiBCH1* (Figure S8a). Dual‐luciferase assay results demonstrated that MiAGL5 activated the transcription of *MiBCH1* (Figure S8b). The EMSA results confirmed that MiAGL5 bound directly to the GArG motif located at −1006 bp to −1019 bp in the *MiBCH1* promoter (Figure S8c,d). These results indicated that, besides *MiZEP*, MiAGL5 can also directly bind to the promoter of *MiBCH1* and activate its expression in mango fruit flesh.

### Identification of the MiGAIP1 and its effect on *MiAGL5* transcription

**Figure 5:**
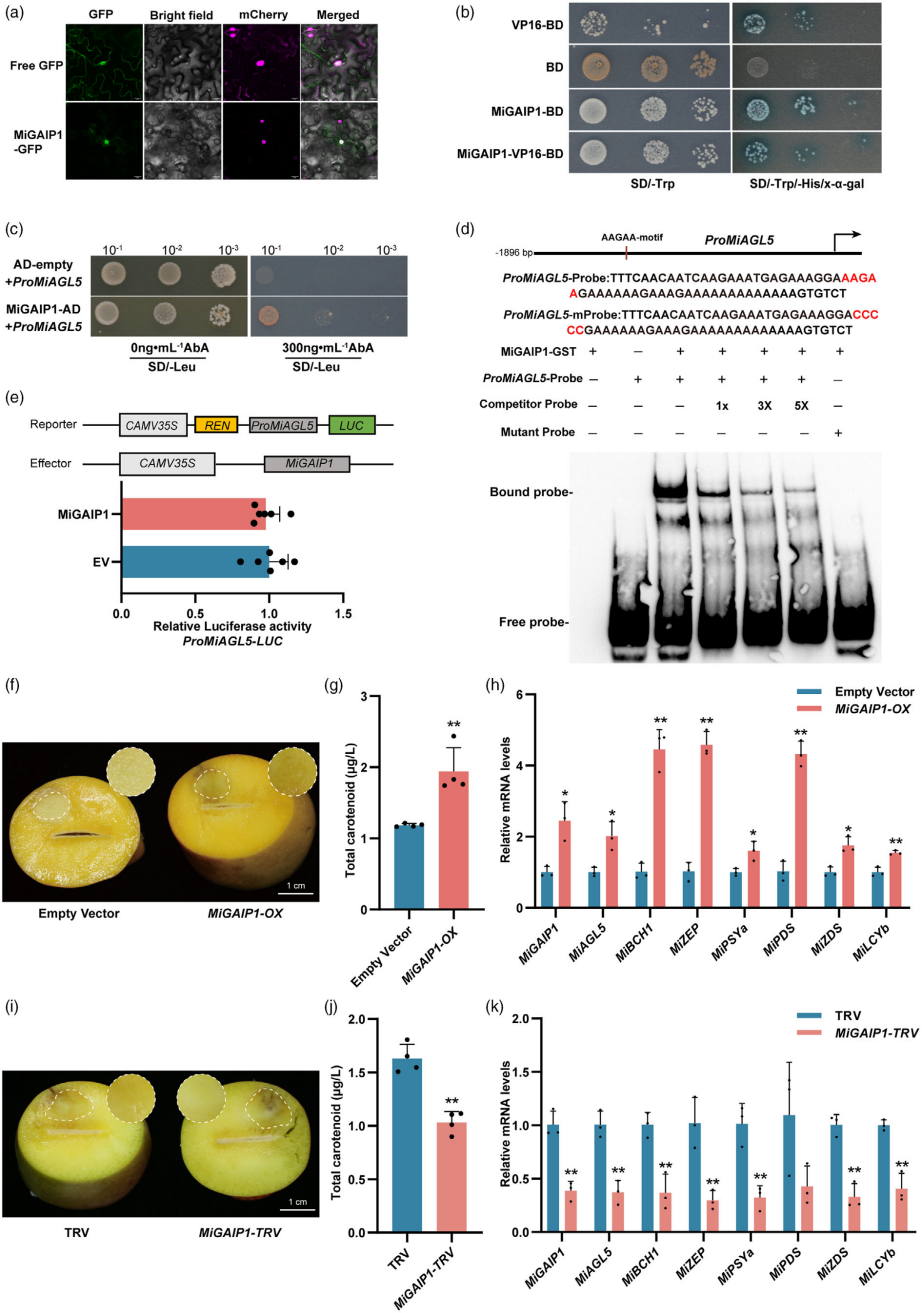
Identification of MiGAIP1 and its effect on *MiAGL5* transcript levels. (a) Subcellular localization of MiGAIP1 in *Nicotiana benthamiana* leaf cells. (b) Transcriptional activation of MiGAIP1 in yeast cells. (c) Y1H assays showing the binding of MiGAIP1 to the *MiAGL5* promoter. AD‐empty (pGADT7) served as a negative control. (d) Binding of the MiGAIP1 protein to the AAGAA motif on the *MiAGL5* promoter. EMSA was performed using a biotin‐labelled *MiAGL5* promoter fragment containing the AAGAA motif, while the competitor probe was an unlabelled probe identical to the labelled probe (1‐, 3‐, 5‐fold molar excess). The mutant probe was identical to the labelled probe, but with the AAGAA motif mutated to CCCCC. The MiGAIP1‐GST protein used for the EMSA was purified. (e) Dual‐luciferase assay revealing the relative activation of MiGAIP1 to the promoter of *MiAGL5* in *Nicotiana benthamiana* leaf cells. The promoter region of *MiAGL5* was inserted into the pGreenII 0800‐*LUC* vector, while the CDS of *MiGAIP1* was inserted into the SK vector. The empty vector of SK was used as the control. Relative luciferase activity was analysed. (f) Phenotypes of *MiGAIP1*‐overexpressing (*MiGAIP1‐OX*) mango fruit flesh. Mango fruits were subjected to infiltration with *A. tumefaciens* EHA105 cells carrying the pCAMBIA1301‐MiGAIP1. Empty vector was used as negative control. Fruit samples were collected 7 days after infiltration. Scale bar = 1 cm. (g) The total carotenoid content in mango fruit flesh with *MiGAIP1*‐overexpression. (h) Expression patterns of carotenoid biosynthesis‐related genes in *MiGAIP1*‐overexpressing mango flesh. (i) Representative images of *MiGAIP1*‐silenced (*MiGAIP1‐TRV*) mango fruit flesh. The empty vector (pTRV1 + pTRV2) was used as negative control. (j) Total carotenoid content in *MiGAIP1*‐silenced mango fruit flesh. (k) Expression patterns of carotenoid biosynthesis‐related genes in *MiGAIP1* transiently silenced flesh. Error bars represent the standard deviation of 3 biological replicates. * indicates statistically significant differences (*P < 0.05 and **P < 0.01) as determined by a Student's *t‐*test.

To elucidate how MiAGL5 responds to blue light, we used a Y1H system to identify its potential upstream TFs. The screening identified a DELLA protein, MiGAIP1, which was homologous to AtRGA1, CsGAIP‐B, CrRGA and GmGAIP1 (Figure S9). Subcellular localization assays and yeast transcriptional activation assays demonstrated that MiGAIP1 is localized to the nucleus and may have transcriptional activation functions (Figure 5a,b). To further verify the binding of MiGAIP1 to the *MiAGL5* promoter, the Y1H assay was performed. Our results indicated that MiGAIP1 directly binds to the promoter of *MiAGL5* in yeast cells (Figure 5c). The EMSA results demonstrated that MiGAIP1 binds to the AAGAA motif in the promoter of *MiAGL5* (Figure 5d). However, the dual‐luciferase assay revealed that MiGAIP1 had no effect on the *MiAGL5* promoter activity (Figure 5e). Similar to the DELLA proteins in *Arabidopsis* (Griffiths *et al*., 2006; Willige *et al*., 2007; Xu *et al*., 2021), the yeast two‐hybrid assay showed that MiGAIP1 interacts with SLY1 (SLEEPY1, mediating the GA pathway by interacting with DELLA proteins and promoting their ubiquitination and subsequent degradation) and depends on GA_3_ to interact with GID1 (GA‐INSENSITIVE DWARF1, GA receptor) (Figures S10 and S11) (Ariizumi *et al*., 2011; Sun, 2011).

To further investigate the role of MiGAIP1 in *MiAGL5* expression and carotenoid biosynthesis, we conducted transient overexpression and VIGS experiments in mango fruit flesh. The overexpression of *MiGAIP1* (*MiGAIP1‐OX*) resulted in a more intense yellow colouration around the injection sites compared to the control (Figure 5f). The carotenoid content in *MiGAIP1‐OX* mango fruit flesh was significantly higher than in the control (Figure 5g). The expression of most of the genes involved in carotenoid biosynthesis in *MiGAIP1‐OX* mango flesh, including *MiAGL5*, *MiBCH1*, *MiZEP*, *MiPSYa*, *MiPDS*, *MiZDS* and *MiLCYb*, was remarkably upregulated compared to the control (Figure 5h). Furthermore, the silencing of *MiGAIP1* (*MiGAIP1‐TRV*) inhibited the yellow colouration and carotenoid biosynthesis in mango flesh (Figure 5i,j). In *MiGAIP1‐TRV* fruit flesh, the expression levels of *MiAGL5*, *MiBCH1*, *MiZEP*, *MiPSYa*, *MiPDS*, *MiZDS* and *MiLCYb* were downregulated (Figure 5k). These results demonstrated that MiGAIP1 induced MiAGL5 expression and carotenoid biosynthesis in mango fruit flesh.

### MiGAIP1 interacts with MiPIF5 to hinder the inhibition of MiPIF5 on MiAGL5

**Figure 6:**
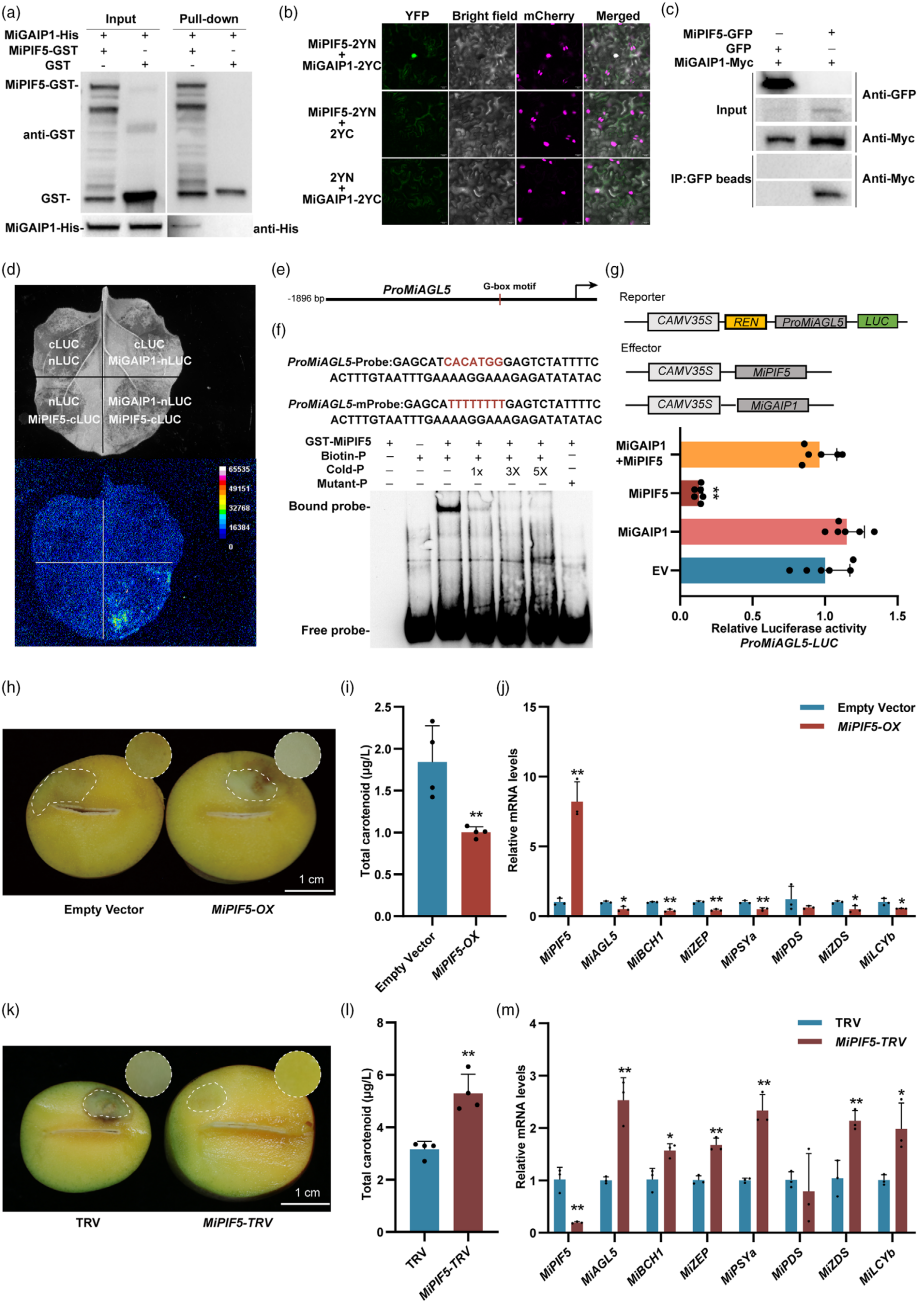
The interaction between MiGAIP1 and MiPIF5 disrupts the transcriptional inhibition of MiPIF5 on MiAGL5. (a) Pull‐down assay showed the interaction between MiGAIP1 and MiPIF5. The MiGAIP1‐His and MiPIF5‐GST fusion proteins were expressed in *E. coli* BL 21 and purified. MiPIF5‐GST was bound to GST magnetic beads. The assays were analysed using immunoblotting with anti‐GST and anti‐His antibodies. The band detected by the anti‐His antibody in the pulled‐down samples indicates an interaction between MiGAIP1 and MiPIF5. (b) Physical interaction between MiGAIP1 and MiPIF5 was confirmed by BiFC assay. (c) Co‐IP assay showed the interaction between MiGAIP1 and MiPIF5. GFP‐tagged MiPIF5 and Myc‐tagged MiGAIP1 were co‐overexpressed in *Nicotiana benthamiana* leaves. GFP magnetic beads were used to capture GFP and MiPIF5‐GFP. Anti‐GFP and anti‐Myc antibodies were used for immunoblotting analysis. Co‐overexpression of GFP and MiGAIP1‐Myc was used as a control. The band detected by the anti‐Myc antibody in the IP samples indicates an interaction between MiGAIP1 and MiPIF5. (d) Split‐luciferase complementation imaging assays indicated that MiGAIP1 interacts with MiPIF5 in *Nicotiana benthamiana* leaves. nLUC and cLUC were used as negative controls. Chemiluminescence generated by luciferase in these regions was imaged 5 min after injecting luciferin. (e) Analysis of the G‐box motif in the *MiAGL5* promoter. (f) EMSA indicated that MiPIF5 directly binds to the *MiAGL5* promoter via the G‐box motif. The EMSA was conducted with a biotin‐labelled *MiAGL5* promoter fragment containing the G‐box motif (CACATGG), whereas the competitor was an unlabelled probe identical to *MiAGL5* promoter fragment (at 1‐, 3‐, 5‐fold molar excess). The mutant probe was identical to the labelled probe, but the CACATGG was mutated to TTTTTTT. The MiPIF5‐GST protein used for the EMSA was purified. (g) Dual‐luciferase assay indicated the effect of MiPIF5 and MiGAIP1–MiPIF5 complex on the activities of *MiAGL5* promoter. The *MiAGL5* promoter was cloned into the pGreenII 0800‐*LUC* vector, and the full‐length CDS of *MiGAIP1* and *MiPIF5* were cloned into the SK vector, respectively. Combinations of MiGAIP1 and MiPIF5 were co‐infiltrated with the *MiAGL5* promoter into *Nicotiana benthamiana* leaves. The empty SK vector was used as a control. Relative luciferase activity was analysed. (h) The overexpression of *MiPIF5* significantly inhibited carotenoid biosynthesis in mango fruit flesh. Mango fruits were subjected to infiltration with *A. tumefaciens* EHA105 cells carrying the pCAMBIA1301‐MiPIF5. Empty vector was served as the negative control. (i) Total carotenoid content in *MiPIF5*‐overexpressing mango fruit flesh. (j) The expression patterns of carotenoid biosynthesis‐related genes in *MiPIF5*‐overexpressing mango flesh. (k) Representative images of mango fruit flesh silenced for *MiPIF5* (*MiPIF5‐TRV*). The empty vector (pTRV1 + pTRV2) served as the negative control. (l) Total carotenoid content in *MiPIF5*‐silenced mango fruit flesh. (m) The expression patterns of carotenoid biosynthesis‐related genes in *MiPIF5*‐silenced fruit flesh. Scale bar = 1 cm. Error bars represent the standard deviation of 3 biological replicates. * indicates statistically significant differences (*P < 0.05 and **P < 0.01) as determined by a Student's *t‐*test.

Considering that MiGAIP1 directly binds to the promoter of *MiAGL5* without an activation effect, but the transient expression of *MiGAIP1* significantly affected *MiAGL5* expression, it is reasonable to speculate that MiGAIP1 might regulate *MiAGL5 via* its interacting protein. PIFs are important TFs in the light response process. Previous reports have shown that PIF3 and PIF4 directly interact with DELLA proteins (de Lucas *et al*., 2008; Feng *et al*., 2008). To identify potential partner proteins of MiGAIP1, we screened two blue light‐responsive MiPIFs, including *MiPIF1* (mango021722) and *MiPIF5* (mango026944), from the transcriptome data. Yeast two‐hybrid showed that MiPIF1 could not interact with MiGAIP1 (Figure S12). A pull‐down assay demonstrated that MiPIF5 interacted with MiGAIP1 *in vitro* (Figure 6a). Subcellular localization demonstrated that MiPIF5 localized in the nucleus (Figure S13a). qPCR assays showed that the expression of *MiPIF5* is downregulated under blue light (Figure S13b). Furthermore, BiFC assay, Co‐IP and split‐luciferase complementation assays confirmed the interaction between MiPIF5 and MiGAIP1 *in vitro* and *in vivo* (Figure 6b–d). Accordingly, these results suggest that MiGAIP1 directly interacts with MiPIF5.

EMSA assay confirmed that MiPIF5 directly binds to the G‐box of the *MiAGL5* promoter (Figure 6e,f). Dual‐luciferase assay showed that MiPIF5 inhibited the activity of the *MiAGL5* promoter (Figure 6g). These results indicated that MiPIF5 directly represses *MiAGL5* transcription by binding to the G‐box of the promoter. To further investigate the role of MiPIF5 in the regulation of *MiAGL5* and carotenoid biosynthesis, the results demonstrated that the injection region overexpressing *MiPIF5* showed lighter yellow colouration and decreased total carotenoid content compared with the control (Figure 6h,i). Gene expression analysis revealed that overexpression of *MiPIF5* downregulated the expression of *MiAGL5*, *MiBCH1*, *MiZEP*, *MiPSYa*, *MiZDS* and *MiLCYb* (Figure 6j). In contrast, silencing of *MiPIF5* significantly increased the total carotenoid content in mango flesh (Figure 6k,l), with an upregulation of *MiAGL5*, *MiBCH1*, *MiZEP*, *MiPSYa*, *MiZDS* and *MiLCYb* (Figure 6m). These results suggested that MiPIF5 negatively regulates carotenoid biosynthesis by inhibiting the expression of *MiAGL5*. We then aimed to investigate the effects of the interaction between MiPIF5 and MiGAIP1 on the expression of *MiAGL5*. Dual‐luciferase assay showed that co‐injection of MiPIF5 and MiGAIP1 hindered the inhibitory effect of MiPIF5 on *MiAGL5* (Figure 6g). Taken together, MiPIF5 represses *MiAGL5* transcription by directly binding to its promoter, but MiGAIP1 hinders the inhibitory effect of MiPIF5 on *MiAGL5* by interacting with MiPIF5.

### Blue light‐activated MiCRY1 interacts with MiGAIP1 and enhances MiGAIP1 protein stability

**Figure 7:**
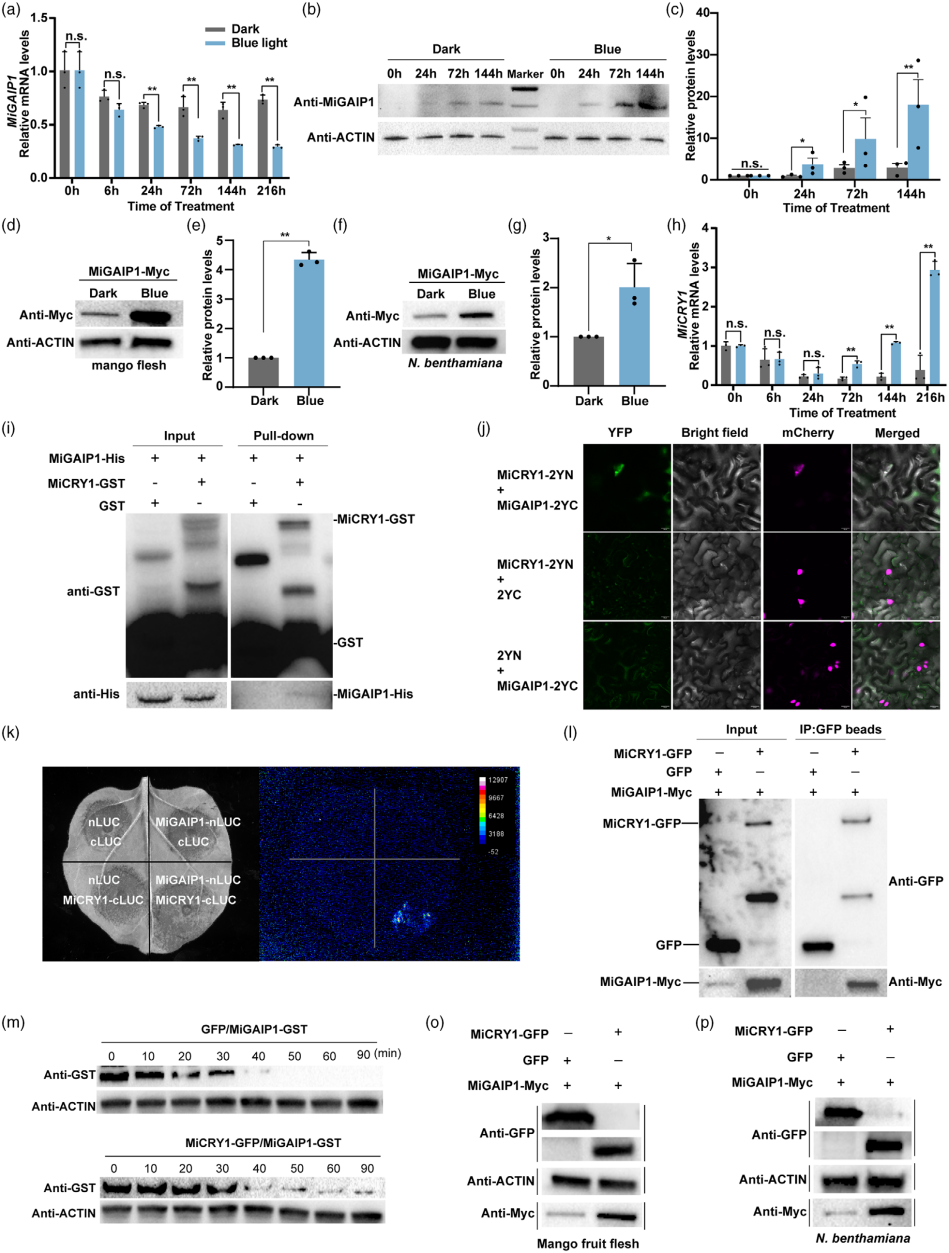
MiCRY1 Interacts with MiGAIP1 to increase its protein stability. (a) Expression pattern of *MiGAIP1* in mango fruit flesh under blue light treatment. *MiActin* was used as an internal reference. (b) Changes in the MiGAIP1 protein abundance in mango fruit flesh. Mango flesh samples that exposed to blue light and darkness for 0, 24, 72 and 144 h were selected. The MiGAIP1 protein was detected by Western blot analysis using a polyclonal anti‐MiGAIP1 antibody. (c) Expression patterns of relative MiGAIP1 protein levels. The band intensity was measured using Image Lab software (version 6.1) and quantitatively normalized with 0 h as the reference. (d–g) Effects of blue light on the abundance of MiGAIP1 protein in mango fruit (d) and *Nicotiana benthamiana* (f). Mango fruit and tobacco leaves overexpressing *MiGAIP1‐Myc* were kept in darkness for 1 day and then transferred to continuous blue light (110 μmol/m^2^/s) for 2 days. MiGAIP1‐overexpressing mango fruit and *Nicotiana benthamiana* kept in continuous darkness were used as controls. Proteins were extracted from mango flesh and *Nicotiana benthamiana* leaves overexpressing MiGAIP1‐Myc and coimmunoprecipitated proteins were probed by anti‐Myc antibody. The relative protein levels of MiGAIP1‐Myc in mango flesh (e) and *Nicotiana benthamiana* leaves (g). (h) The relative expression levels of *MiCRY1* in mango fruit flesh under blue light treatment. (i) Pull‐down assay showed that MiGAIP1 interacts with MiCRY1 *in vitro*. Immobilized GST and MiCRY1‐GST (mango021673) were used to pull down MiGAIP1‐His, and immunoprecipitated fractions were detected using anti‐His antibody. The bait proteins were probed with anti‐GST antibody. (j) Physical interaction between MiGAIP1 and MiCRY1 was confirmed by BiFC assay. (k) Split‐luciferase complementation imaging assays showed the interaction between MiGAIP1 and MiCRY1 in *Nicotiana benthamiana* leaves. nLUC and cLUC were used as negative controls. Chemiluminescence generated by luciferase in these regions was imaged 5 min after injecting luciferin. (l) Co‐IP assay showed the interactions between MiGAIP1 and MiCRY1. GFP‐tagged MiCRY1 and Myc‐tagged MiGAIP1 were co‐overexpressed in *Nicotiana benthamiana* leaves. GFP magnetic beads were used to capture GFP and MiCRY1‐GFP. Anti‐GFP and anti‐Myc antibodies were used for immunoblotting analysis. Co‐overexpression of GFP and MiGAIP1‐Myc served as a control. The band detected by the anti‐Myc antibody in the IP samples indicates an interaction between MiGAIP1 and MiCRY1. (m) Time course of MiGAIP1 degradation in protein extracts of *Nicotiana benthamiana* leaves infiltrated with GFP or MiCRY‐GFP protein. Equal amounts of MiGAIP1‐GST were added to crude plant extracts. Anti‐Actin was used as a loading control. MiGAIP1‐GST proteins were expressed in *E. coli* BL 21 and purified. (o, p) Transient overexpression assays showed that MiCRY1 stabilized MiGAIP1 protein in mango fruit flesh (o) and *Nicotiana benthamiana* leaves (p). GFP with MiGAIP1‐Myc, and MiCRY1‐GFP with MiGAIP1‐Myc were co‐overexpressed in mango flesh and *Nicotiana benthamiana* leaves. MiGAIP1 was detected by immunoblotting with anti‐Myc antibodies. Anti‐GFP antibodies were used for immunoblotting analysis to detect MiCRY1‐GFP and GFP. Anti‐Actin antibodies were used to quantify the total amount of protein loaded in each lane. Error bars represent the standard deviation of 3 biological replicates. * indicates statistically significant differences (*P < 0.05 and **P < 0.01) as determined by a Student's *t‐*test.

Given that MiGAIP1 promotes carotenoid biosynthesis and its expression is downregulated under blue light (Figure 7a), we speculated that the protein level of MiGAIP1 might be induced by blue light. We extracted mango flesh proteins and detected MiGAIP1 protein using the MiGAIP1‐specific antibody. The results showed that the MiGAIP1 protein was significantly accumulated after blue light treatment compared with the dark control from 24 h to 144 h (Figure 7b,c). We also analysed the response of MiGAIP1 protein levels to blue light using the transient overexpressing assay. The results showed that the levels of MiGAIP1 protein were significantly upregulated in mango fruit flesh and tobacco leaves treated with blue light compared to the dark control (Figure 7d–g). Considering that CRY1 interacts with GID1 in a blue light‐dependent manner to inhibit the degradation of DELLA protein (Xu *et al*., 2021), we identified two *MiCRY2s* and two *MiCRY1s* (Ni *et al*., 2022). The Y2H assays suggested that the two MiCRY2s did not interact with MiGAIP1 (Figure S14). Pull‐down assays showed that MiGAIP1 interacts with MiCRY1 (mango024864) (Figure 7i), but not with MiCRY1 (mango021673) *in vitro* (Figure S15). Thus, MiCRY1 (mango024864) was selected for further investigation. qPCR assays showed that *MiCRY1* was upregulated under blue light (Figure 7h). BiFC, split‐luciferase complementation and Co‐IP assays confirmed the interaction between MiCRY1 and MiGAIP1 both *in vitro* and *in vivo* (Figure 7j–l). These results indicated that MiCRY1 interacts with MiGAIP1.

To investigate the effect of MiCRY1 on MiGAIP1 protein stability, we performed a cell‐free protein degradation assay. The results showed that MiGAIP1 was degraded more slowly in extracts with MiCRY1‐GFP protein than with GFP protein (Figure 7m). To determine whether MiCRY1 increases the stability of MiGAIP1 protein *in vivo*, we transiently co‐overexpressed MiGAIP1‐Myc and MiCRY1‐GFP in mango flesh and *Nicotiana benthamiana* leaves. Immunoblotting analysis revealed that the MiGAIP1 protein levels were significantly increased by co‐overexpression of *MiCRY1* compared to the control (Figure 7o,p). These results demonstrate that blue light increases MiGAIP1 protein levels by promoting its stability through the interaction between MiCRY1 and MiGAIP1.

### Blue light and GA antagonistically regulate carotenoid biosynthesis via MiGAIP1 protein as a hub

**Figure 8:**
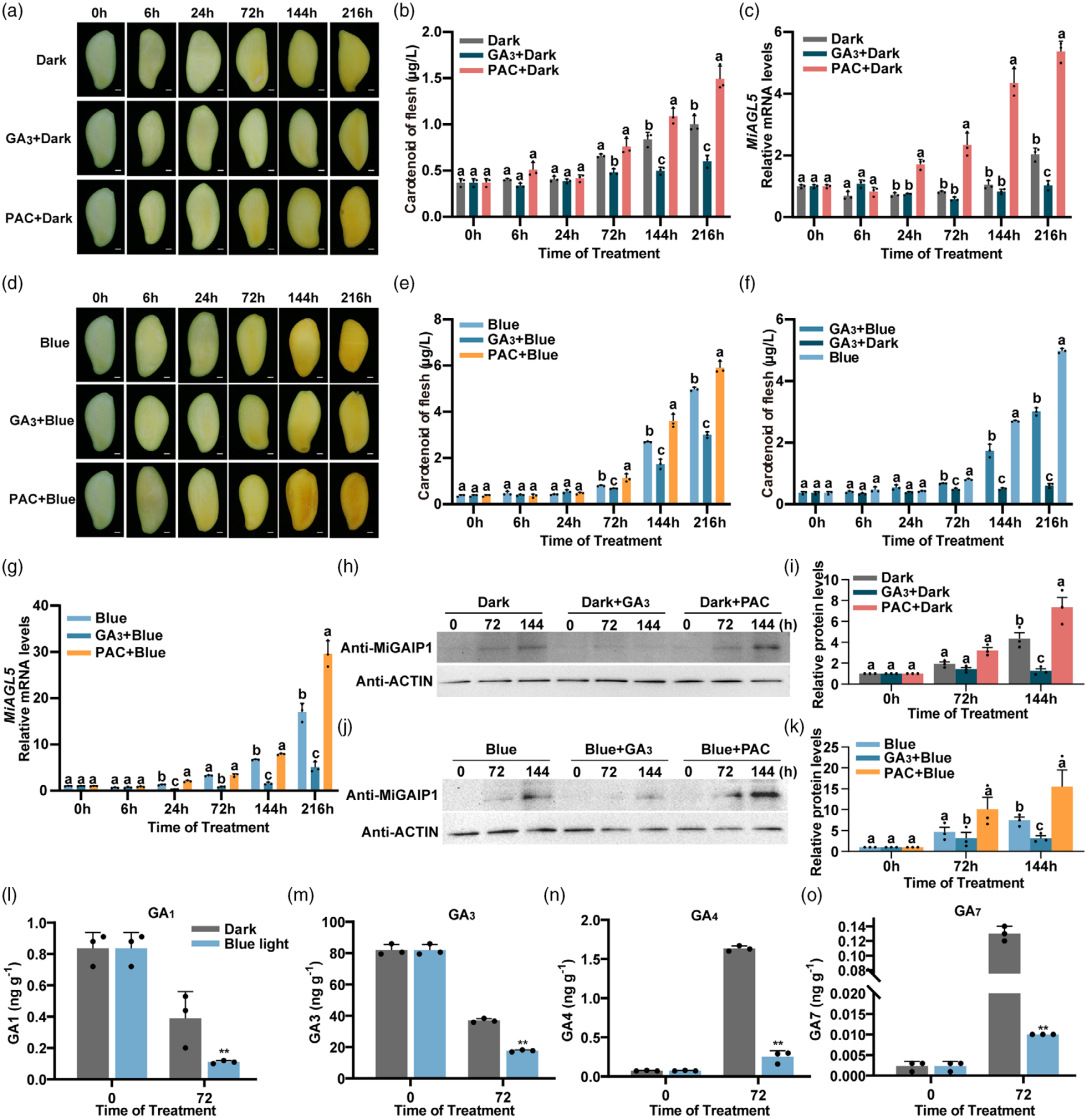
GA_3_ and blue light antagonistically regulate the carotenoid biosynthesis and the yellowing of mango fruit flesh. (a) Representative images of mango flesh treated in the dark (control), GA_3_ + dark and PAC + dark. Scale bars, 1 cm. (b) Total carotenoid content of mango flesh treated by dark (control), GA_3_ + dark and PAC + dark. (c) mRNA levels of *MiAGL5* were determined using RT‐qPCR. The numbers below X axis indicate the time after treatment. (d) Representative images of mango flesh treated with blue light (control), GA_3_ + blue light and PAC + blue light. (e) Total carotenoid content of mango flesh treated with blue light (control), GA_3_ + blue light and PAC + blue light. (f) Total carotenoid content in mango fruit flesh treated by blue light, GA_3_ + dark, and GA_3_ + blue light. (g) Expression pattern of *MiAGL5* in mango fruit flesh treated by blue light (control), GA_3_ + blue light and PAC + blue light. (h) Immunoblot analysis of MiGAIP1 protein levels in mango fruit flesh under dark (control), GA_3_ + dark and PAC + dark treatments. (i) Relative protein levels of MiGAIP1 in mango fruit flesh under dark, dark + GA_3_ and dark + PAC treatments. (j) Immunoblot analysis of MiGAIP1 protein levels under blue light, blue light + GA_3_ and blue light + PAC treatments. (k) Relative protein levels of MiGAIP1 in mango fruit flesh under blue light, blue light + GA_3_ and blue light + PAC treatments. Image Lab software was used to detect band intensity. All ratios were normalized using 0 h as the reference. (l–o) The levels of four bioactive GA in blue light‐treated mango fruit flesh. GA measurements were taken from mango fruit flesh samples at 0 h and 72 h under blue light and darkness. The analysis focused on four bioactive GA: GA_1_ (l), GA_3_ (m), GA_4_ (n) and GA_7_ (o). Darkness served as a control. Error bars represent the standard deviation of three biological replicates. * indicates statistically significant differences (*P < 0.05 and **P < 0.01) as determined by a Student's *t‐*test. Different lowercase letters above the error bars indicate significant differences, as determined by one‐way ANOVA (Tukey) (*P* < 0.05).

Considering that GA induces the degradation of DELLA proteins (Sun and Li, 2020), while our finding showed that blue light stabilized DELLA protein MiGAIP1, we suspected that GA might inhibit carotenoid biosynthesis in mango fruit flesh and play antagonistic roles with blue light. ‘Guifei’ mango fruit were treated with GA_3_ or PAC (paclobutrazol, a GA biosynthetic inhibitor) under darkness and blue light. The results showed that exogenous application of GA_3_ in the darkness significantly inhibited the yellowing process and decreased the total carotenoid content of the mango fruit flesh, while PAC promoted it (Figure 8a,b). qPCR assays showed that the expression patterns of *MiAGL5* were downregulated under GA_3_ treatment compared to the control, while their expression patterns were upregulated under PAC treatment (Figure 8c). *MiPSYa*, *MiBCH1* and *MiZEP* showed similar expression patterns to *MiAGL5* (Figure S16a–c). Furthermore, GA_3_ + blue light treatment accelerated the yellowing process and carotenoid accumulation of mango fruit flesh compared with GA_3_ + Dark treatment, while it reduced carotenoid accumulation compared with blue light treatment (Figure 8a,d,f). Conversely, PAC + blue light treatment significantly increased carotenoid accumulation compared with PAC treatment or blue light treatment (Figure 8b,e). qPCR assays revealed that the expression levels of *MiAGL5*, *MiPSYa*, *MiBCH1* and *MiZEP* were downregulated under blue light + GA_3_ treatment compared to the control, while their expression levels were upregulated under blue light + PAC treatment (Figures 8g and S16d–f). These results indicated that blue light and GA antagonistically regulate carotenoid biosynthesis in mango fruit.

Furthermore, we analysed the protein levels of MiGAIP1 in mango fruit flesh treated with GA_3_ or PAC under darkness and blue light. Compared to the dark treatment, the protein level of MiGAIP1 in mango flesh treated with GA_3_ decreased at 144 h. In contrast, the protein level of MiGAIP1 increased under PAC treatment (Figure 8h,i). In addition, mango fruit flesh under blue light + GA_3_ conditions showed a slight decrease in MiGAIP1 protein levels compared to the control, while the accumulation of MiGAIP1 protein increased under blue light + PAC treatment (Figure 8j,k). These results indicated that MiGAIP1 acts as a pivotal integrator mediating the antagonistic effects of blue light and GA in regulating carotenoid biosynthesis.

### Blue light reduces GA content in mango fruit flesh to stabilize MiGAIP1 protein

To investigate the effect of blue light on GA content in mango fruit flesh, the content of four bioactive GA was measured. The results showed that the content of GA_1_ and GA_3_ in mango fruit flesh significantly decreased under blue light compared to the control (Figure 8l,m). The levels of GA_4_ and GA_7_ were higher at 72 h than at 0 h. However, the levels of GA_4_ and GA_7_ significantly decreased under blue light compared to the dark at 72 h (Figure 8n,o). Taken together, these results indicated that blue light significantly reduced the GA content in mango fruit flesh, which could further lead to an increase in the abundance of the key regulator MiGAIP1 protein, which in turn promotes carotenoid biosynthesis.

## Discussion

### Blue light‐induced MiAGL5 positively regulates carotenoid biosynthesis in mango fruit by activating *MiZEP* and *MiBCH1* transcription

In this study, we found that blue light significantly promotes carotenoid biosynthesis in mango fruit flesh (Figure 1), which is consistent with our previous research in mango fruit (Ni *et al*., 2022) and other groups' researches in different fruits including mandarin (Yuan *et al*., 2017), strawberry (Chen *et al*., 2023) and peach (Cao *et al*., 2017). MADS‐box TFs are key components of the regulatory network that regulates carotenoid biosynthesis. Classic MADS‐box TFs, including RIN, FUL1, FUL2 and TAGL1, have been shown to positively regulate the transcription of *PSY1*, *PDS* and *ZDS*, thereby promoting lycopene biosynthesis in tomato (Dong *et al*., 2014; Shima *et al*., 2013; Vrebalov *et al*., 2009). In contrast, MADS1 functions as a negative regulator of *PSY1*, while MBP8, another MADS‐box TF, inhibits the expression of *PSY1*, *PDS* and *ZDS*, thereby suppressing lycopene biosynthesis in tomato (Dong *et al*., 2013; Yin *et al*., 2017). In *Citrus* hesperidium, CsMADS3 positively regulates the expression of *CsPSY1* and *CsLCYb2* to promote carotenoid biosynthesis (Zhu *et al*., 2023). In kiwifruit, AcMADS32 has been shown to positively regulate carotenoid biosynthesis by activating the transcription of *AcBCH1/2* (Xia *et al*., 2023). The majority of studies on MADS‐box TFs in regulating the carotenoid biosynthesis pathway have focused on the regulation of genes including *PSY*, *PDS*, *ZDS*, *LCYb* and *BCH*, while research on the regulation of *ZEP* remains limited.

Violaxanthin is the most abundant carotenoid in the blue light‐treated mango fruit flesh (Ni *et al*., 2022), and the expression pattern of the violaxanthin synthesis gene *MiZEP* was highly and positively correlated with the carotenoid accumulation pattern (Figure 1c,i). Therefore, we speculated that blue light promotes violaxanthin biosynthesis mainly by regulating the expression of *MiZEP*. By transcriptome analysis, we identified a MADS TF, MiAGL5, which directly activated *MiBCH1* and *MiZEP* by binding to their promoters (Figures 3e–g and S8). The expression levels of *MiBCH1* and *MiZEP* in *MiAGL5*‐overexpressing mango fruit flesh were significantly higher than those in the control (Figure 4f), providing further evidence for MiAGL5‐induced expression of *MiBCH1* and *MiZEP*. Additionally, overexpression of *MiAGL5* significantly increased the levels of violaxanthin in mango fruit flesh (Figure 4g), indicating that blue light‐induced MiAGL5 promoted the biosynthesis of the major carotenoid compound, violaxanthin, by activating *MiBCH1* and *MiZEP*. Taken together, these results suggest that MADS‐box proteins in different species may adapt to their unique carotenoid accumulation patterns by selectively targeting different carotenoid biosynthetic genes. The divergence in target genes of MADS‐box proteins may be closely related to the evolutionary need to produce different types of carotenoid compounds, such as lycopene in tomato versus violaxanthin in mango fruit.

### MiGAIP1 hinders the inhibitory effect of MiPIF5 on *MiAGL5* to positively regulate carotenoid biosynthesis in mango fruit

In *Arabidopsis* and tomato, several PIF family members negatively regulate lycopene accumulation by specifically repressing the expression of *PSY* (Llorente *et al*., 2016; Toledo‐Ortiz *et al*., 2010). However, research on the regulation of genes and compounds in the *β*‐branch of carotenoid biosynthesis by PIFs remains limited. The results of this study indicated that blue light inhibited MiPIF5 transcriptionally repressed *MiAGL5* expression, which could directly activate *MiBCH1* and *MiZEP* expression, suggesting that MiPIF5 could inhibit the biosynthesis of *β*‐branch carotenoid compounds through MiAGL5 (Figure 6). These results provide new insights into the transcriptional mechanisms that govern MADS‐box TFs related to carotenoid biosynthesis.

In citrus, the overexpression of *CsMADS3* leads to an accumulation of ABA and a reduction of GA. Additionally, changes in GA may exert a negative feedback regulation on CsMADS3 and carotenoid biosynthesis (Zhu *et al*., 2023), suggesting a potential interconnection among MADS TFs, GAs and carotenoid biosynthesis. DELLA proteins are the core regulators of the GA signalling pathway (Silverstone *et al*., 2001). In maize sprouts, it is hypothesized that different light qualities stimulate DELLA proteins, thereby inducing carotenoid biosynthesis (Xiang *et al*., 2022). Similarly, in etiolated *Arabidopsis* cotyledons, the accumulation of DELLAs in darkness is known to depress carotenoid biosynthesis, thereby preventing light‐induced oxidative damage in seedlings (Cheminant *et al*., 2011). However, the molecular mechanisms by which DELLAs regulate carotenoid biosynthesis remain largely unexplored. In the present study, MiGAIP1, a DELLA protein, was identified as an upstream regulator of *MiAGL5* (Figure 5a–d), but MiGAIP1 could not activate *MiAGL5* in a dual‐luciferase assay (Figure 5e). Nevertheless, the expression of *MiGAIP1* was induced or inhibited, respectively, by overexpressing or silencing of *MiAGL5* in mango fruit (Figure 5f–k). It has been reported that DELLA proteins probably function by interacting with some TFs and impeding their transcriptional activities. DELLA proteins interact with several TFs, including PIF3/PIF4, AUXIN RESPONSE FACTORs (ARFs), and BRASSINAZOLE‐RESISTANT 1 (BZR1) proteins, to repress their DNA binding activity to regulate photomorphogenesis, auxin, and BR signalling, respectively (Bai *et al*., 2012; de Lucas *et al*., 2008; Feng *et al*., 2008; Oh *et al*., 2014). In this study, we demonstrated that MiGAIP1 physically interacted with MiPIF5, hindering MiPIF5's repression of *MiAGL5* transcription, thereby promoting carotenoid biosynthesis (Figure 6). Thus, the DELLA‐PIF interaction in mango is repurposed to regulate specialized metabolism rather than developmental programs, reflecting use of core regulatory elements for multifunctional purposes during plant evolution. Furthermore, these results also suggest that the antagonistic effects of DELLA proteins and PIFs on different biological processes by protein–protein interaction might be conserved among different species. However, whether the binding ability of GAIP1 is conserved in other species requires further investigation. Furthermore, future studies could consider incorporating more advanced statistical models, such as interaction regression models or Bayesian network analysis, to further elucidate the complex regulatory relationship between MiGAIP1 and MiAGL5.

### The antagonistic regulation of carotenoid biosynthesis by blue light and GA is mediated by the adverse tuning of MiGAIP1 protein stability

Plants capture blue light signals through the photoreceptor cryptochromes (CRYs) and adjust their growth and development accordingly (Cashmore *et al*., 1999). The function of CRY1 has been extensively studied in transgenic *Arabidopsis* plants. Studies have shown that overexpression of *CRY1* in *Arabidopsis* results in a dwarf phenotype and anthocyanin accumulation, while CRY1 mutants exhibit an early flowering phenotype under long‐day conditions (Exner *et al*., 2010; Gu *et al*., 2012; Hong *et al*., 2008). The present study demonstrates that CRY1 promoted carotenoid biosynthesis by stabilizing the GAIP1 protein, thus contributing to the growing body of knowledge regarding the functional role of CRY1 in plants.

In *Arabidopsis*, the well‐studied mechanism of blue light signalling involves the interaction of CRY1 with COP1 to regulate photomorphogenesis and flowering by mediating the degradation of LONG HYPOCOTYL (HY5) and CONSTANS (CO) (Liu *et al*., 2008; Osterlund *et al*., 2019; Wang *et al*., 2001). Recently, CRY1 has been reported to play a role in photomorphogenesis in *Arabidopsis* and leaf senescence in soybean by enhancing the stability of DELLAs (Li *et al*., 2024; Xu *et al*., 2021; Zhong *et al*., 2021). In darkness, GA is recognized by the GID1 protein, which then induces the capture of the DELLA proteins by the GID1‐GA complex, leading to the ubiquitination and subsequent degradation of DELLAs by the SCF^SLY1^. Conversely, upon exposure to blue light, CRY1 has been shown to inhibit the formation of the GA‐GID1‐DELLA complex, thereby preventing the degradation of DELLAs (Xu *et al*., 2021). Similarly, the CRY1‐DELLA signalling module has been demonstrated to inhibit the transcription of *GmWRKY100*, thereby delaying soybean leaf senescence under low blue light (Li *et al*., 2024). In our study, MiCRY1 was shown to interact with MiGAIP1, thereby strengthening its protein stability. This interaction then promoted carotenoid biosynthesis under blue light by hindering MiPIF5's repression on *MiAGL5* transcription (Figure 7).

Furthermore, exogenous treatment of mango fruit with GA_3_ resulted in a reduction in MiGAIP1 protein levels and a hindrance to carotenoid biosynthesis. Conversely, treatment with the GA biosynthesis inhibitor PAC led to increased accumulation of MiGAIP1 protein and stimulation of carotenoid biosynthesis, thereby accelerating the yellowing transition of mango fruit flesh (Figure 8a–g). The results demonstrate an antagonistic relationship between blue light and GA_3_ on MiGAIP1 protein abundance and carotenoid biosynthesis in mango fruit flesh (Figure 8h–k). Previous research has indicated that blue light‐dependent CRYs regulate GA catabolic and metabolic genes, resulting in a reduction in GA concentrations in *Arabidopsis* (Zhao *et al*., 2007). Similarly, exposure to blue light significantly decreased GA_1_ content under the control of CRYs in wild‐type pea plants (Reid *et al*., 2002). The present study revealed that the content of bioactive gibberellins in mango fruit flesh was markedly reduced under blue light (Figure 8l–o). In light of these findings, we put forth the hypothesis that blue light facilitates the accumulation of the MiGAIP1 protein, which in turn promotes carotenoid biosynthesis in mango fruit flesh via the following pathway. First, blue light stabilizes MiGAIP1 protein levels by interacting with MiCRY1. Furthermore, blue light reduces the endogenous GA levels in mango fruit flesh, thereby preventing the GA‐induced degradation of MiGAIP1 and consequently promoting carotenoid biosynthesis. Collectively, our results indicate that MiCRY1 mediates blue light to stabilize MiGAIP1 protein through a dual mechanism (direct protein–protein interaction as well as regulation of GA levels), and the intricate regulatory pattern may facilitate the achievement of homeostasis in blue light‐regulated carotenoid biosynthesis in mango fruit.

However, mango quality is influenced by several environmental factors, including temperature, light intensity and nutrition, in addition to light quality (Nunes *et al*., 2007). Potential confounding environmental factors such as temperature and light could provide novel approaches to improve mango quality. While advances in signal transduction and physiological research have expanded the role of temperature in fruit development, the specific functions and molecular mechanisms by which temperature modulates carotenoid metabolism and mango development remain poorly understood (Nunes *et al*., 2007). The carotenoid metabolic network merits further investigation, particularly to determine whether temperature interacts with blue light signalling pathways to regulate mango fruit development. Additional research is also needed to identify TFs within this network. Furthermore, given the influence of genetic diversity on transcriptional regulation, future investigations incorporating population‐level approaches, such as genome‐wide association studies (GWAS) or quantitative trait locus (QTL) mapping, could provide deeper insights into the regulation of natural variation in carotenoid biosynthesis across different mango genotypes, thereby enhancing the applicability and generalizability of our findings.

## Conclusion

**Figure 9:**
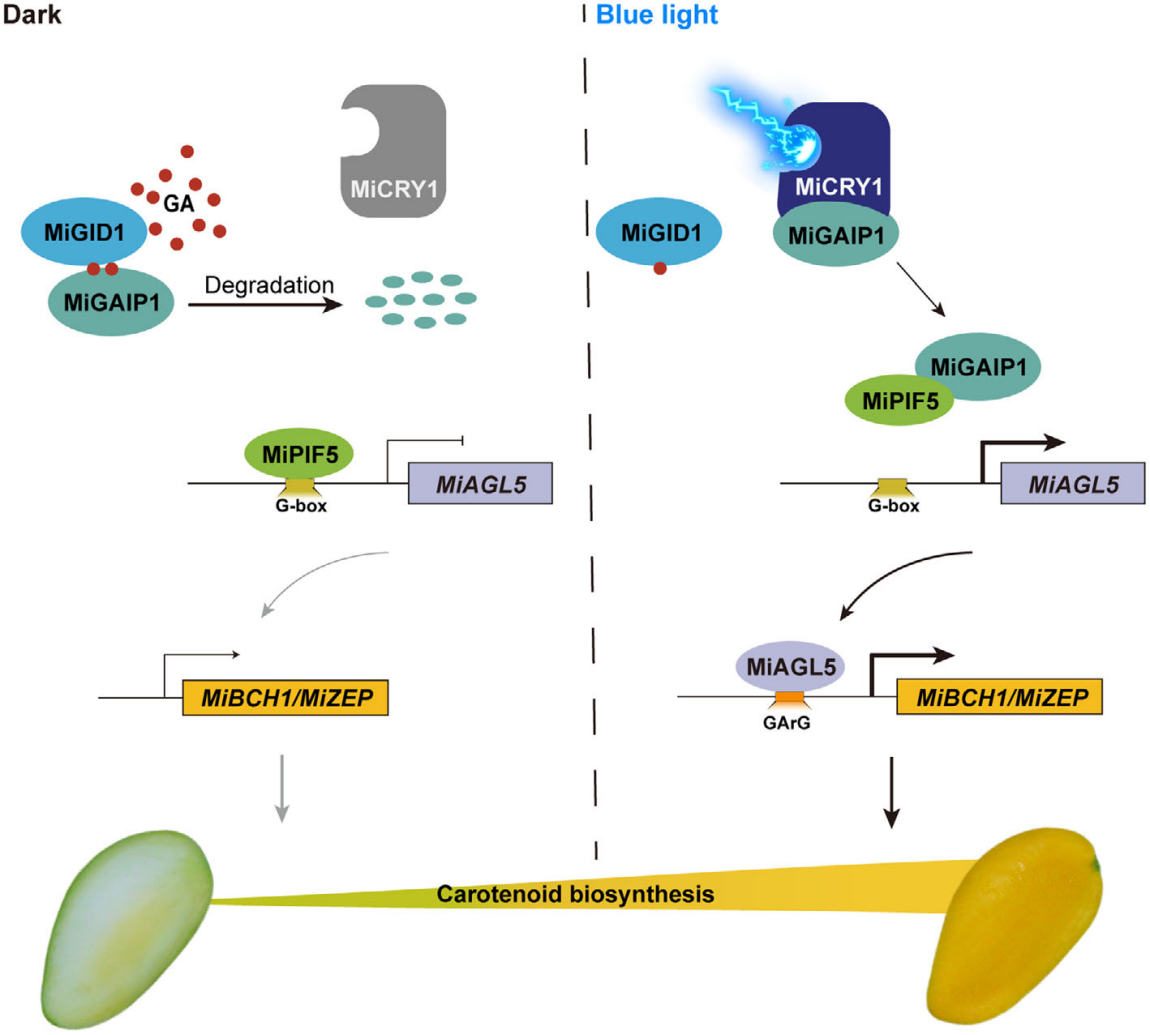
Proposed working model for blue light promotes carotenoid biosynthesis via the MiCRY1‐MiGAIP1‐MiPIF5‐MiAGL5 signalling pathway in mango fruit flesh. In the dark, inactive MiCRY1 is unable to connect with the GA and MiGID1 complex, while GA targets MiGAIP1 for degradation via the 26S proteasome pathway. The transcription repressor MiPIF5 binds to the promoter of *MiAGL5* and inhibits its expression, which further reduces the expression of carotenoid biosynthesis structural genes (*MiZEP* and *MiBCH1*). Under blue light, actived‐MiCRY1 increases the stability of MiGAIP1 protein through physically interactions. Meanwhile, blue light reduces the content of bioactive GA inhibiting the formation of GID1–GA complex. Massive MiGAIP1 interacts with MiPIF5 and alleviates its transcriptional inhibition in MiAGL5, thus promoting the expression of carotenoid structural genes and the biosynthesis of carotenoid in mango fruit flesh.

In summary, the present study elucidates a MiCRY1‐MiGAIP1‐MiPIF5‐MiAGL5 cascade‐mediated regulatory mechanism underlying blue light‐induced carotenoid biosynthesis in mango fruit. In darkness, the increased GA content facilitates the formation of the MiGID1‐GA complex, which binds to the MiGAIP1 protein and leads to its degradation. Concurrently, MiPIF5 binds to the promoter of *MiAGL5* and suppresses its expression, resulting in low expression levels of genes involved in carotenoid biosynthesis (*MiZEP* and *MiBCH1*) and a corresponding reduction in carotenoid content. Under blue light conditions, the activated MiCRY1 directly interacts with MiGAIP1, preventing its capture and subsequent degradation by the GID1‐GA complex. Furthermore, blue light reduces bioactive GA levels, which in turn hinders the formation of the GID1‐GA complex. These actions collectively promote an increase in the abundance of the MiGAIP1 protein in the mango fruit flesh. The interaction between MiGAIP1 and MiPIF5 inhibits MiPIF5's ability to repress *MiAGL5* transcription. MiAGL5 binds directly to the promoters of *MiZEP* and *MiBCH1*, thereby activating their expression and ultimately accelerating carotenoid biosynthesis. Thus, GA and blue light exert opposing effects on carotenoid biosynthesis by influencing the protein abundance of MiGAIP1 (Figure 9). Our results elucidate the molecular mechanisms underlying the blue light signalling‐mediated regulation of carotenoid biosynthesis in mango fruit through crosstalk with plant hormones.

## Author contributions

Y.T. and J.N. conceived and planned the study. M.Z. completed the majority of the experiments. Y.F. helped with completing the data analysis. F.J., Y.L., C.P., J.L., J.W. and R.Q. collected the samples. M.Z., Y.T. and J.N. wrote the manuscript. Q.Y. and S.B. revised the manuscript. All the authors have read and agreed to the published version of the manuscript.

## Conflict of interest

The authors declare no conflict of interest.

## Supporting information


**Figure S1** Representative images of mango flesh after GUS staining following transient expression of the *GUS* reporter gene.
**Figure S2** Functional analysis of MiZEP in ‘*Alisa Craig*’ tomato fruit.
**Figure S3** Heat map showing the differential expression patterns of five candidate genes in control and blue light‐treated mango flesh, as listed in Table S4.
**Figure S4** Expression patterns of candidate transcription factor genes in mango flesh under blue light treatment.
**Figure S5** Assay of transcriptional activation effect of MiERF1, MiERF61L, MiMYB70, and MiMUTE on the *MiZEP* promoter using dual‐luciferase assay.
**Figure S6** Multiple sequence alignment of the MiAGL5 protein in mango and other plants.
**Figure S7** PCR results showed that fragment of pTRV2 was expressed in the flesh which was injected with empty vector as well as MiAGL5‐TRV, while pTRV2 was not expressed in the flesh without any injection.
**Figure S8** MiAGL5 directly binds to the promoter of *MiBCH1* and activates its expression.
**Figure S9** Multiple sequence alignment and phylogenetic tree analysis of MiGAIP1.
**Figure S10** Yeast 2‐hybrid assays showing the interactions between MiGAIP1 and MiSLY1.
**Figure S11** Yeast 2‐hybrid assays showing interactions between MiGAIP1 and GA‐dependent MiGIDs (MiGID1A and MiGID1B).
**Figure S12** Yeast 2‐hybrid assays demonstrating that MiGAIP1 can not interact with MiPIF1 in yeast.
**Figure S13** Identification of MiPIF5.
**Figure S14** Yeast two‐hybrid assays indicated that MiGAIP1 did not interact with either MiCRY1‐mango009201 or MiCRY2‐mango029764.
**Figure S15** Pull‐down assay confirming that MiGAIP1 does not interact with MiCRY1‐mango021673 *in vitro*.
**Figure S16** Expression patterns of *MiPSYa*, *MiBCH1*, and *MiZEP* in mango flesh under treatment.
**Table S1** Carotenoid compound content in the mango flesh of control and overexpressing‐*MiAGL5*.
**Table S2** Primers for qPCR analysis.
**Table S3** Primers used for constructing vectors and EMSA.
**Table S4** FPKM values of five candidate genes in control and blue light‐treated mango flesh.
**File S1** Sequence data for protein.

## Data Availability

The data supporting the article can be obtained by contacting the corresponding author.
