# Supplementary material for: CRY1–GAIP1 complex mediates blue light to hinder the repression of PIF5 on AGL5 to promote carotenoid biosynthesis in mango fruit
Source: Plant Biotechnol J. 2025 Apr 22;23(7):2769–89. doi: 10.1111/pbi.70100 (PMC12205875; doi:10.1111/pbi.70100)
Supplement: Supplementary file 1 — Figure S1 Representative images of mango flesh after GUS staining following transient expression of the GUS reporter gene. Figure S2 Functional analysis of MiZEP in ‘Alisa Craig’ tomato fruit. Figure S3 Heat map showing the differential expression patterns of five candidate genes in control and blue light‐treated mango flesh, as listed in Table S4. Figure S4 Expression patterns of candidate transcription factor genes in mango flesh under blue light treatment. Figure S5 Assay of transcriptional activation effect of MiERF1, MiERF61L, MiMYB70, and MiMUTE on the MiZEP promoter using dual‐luciferase assay. Figure S6 Multiple sequence alignment of the MiAGL5 protein in mango and other plants. Figure S7 PCR results showed that fragment of pTRV2 was expressed in the flesh which was injected with empty vector as well as MiAGL5‐TRV, while pTRV2 was not expressed in the flesh without any injection. Figure S8 MiAGL5 directly binds to the promoter of MiBCH1 and activates its expression. Figure S9 Multiple sequence alignment and phylogenetic tree analysis of MiGAIP1. Figure S10 Yeast 2‐hybrid assays showing the interactions between MiGAIP1 and MiSLY1. Figure S11 Yeast 2‐hybrid assays showing interactions between MiGAIP1 and GA‐dependent MiGIDs (MiGID1A and MiGID1B). Figure S12 Yeast 2‐hybrid assays demonstrating that MiGAIP1 can not interact with MiPIF1 in yeast. Figure S13 Identification of MiPIF5. Figure S14 Yeast two‐hybrid assays indicated that MiGAIP1 did not interact with either MiCRY1‐mango009201 or MiCRY2‐mango029764. Figure S15 Pull‐down assay confirming that MiGAIP1 does not interact with MiCRY1‐mango021673 in vitro. Figure S16 Expression patterns of MiPSYa, MiBCH1, and MiZEP in mango flesh under treatment. Table S1 Carotenoid compound content in the mango flesh of control and overexpressing‐MiAGL5. Table S2 Primers for qPCR analysis. Table S3 Primers used for constructing vectors and EMSA. Table S4 FPKM values of five candidate genes in control and blue light‐treated m [file PBI-23-2769-s001.docx]

Supplemental Figure


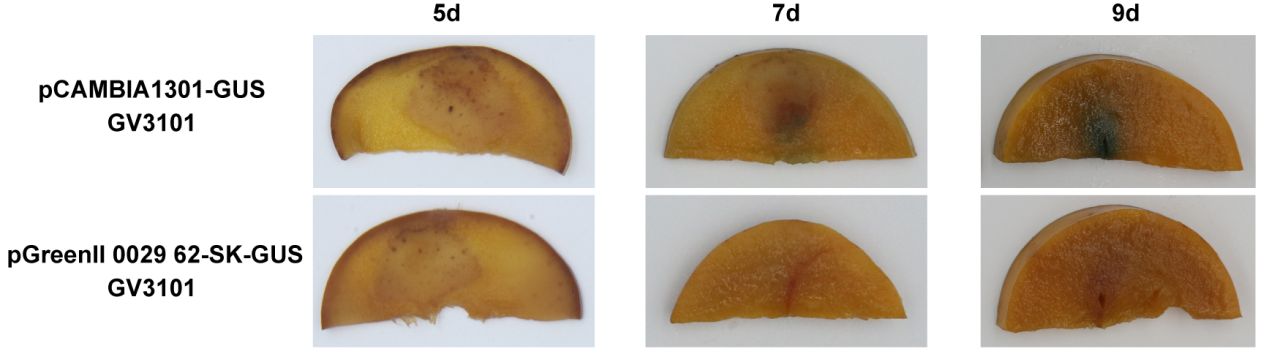


Fig S1 Representative images of mango flesh after GUS staining following transient expression of the *GUS* reporter gene.


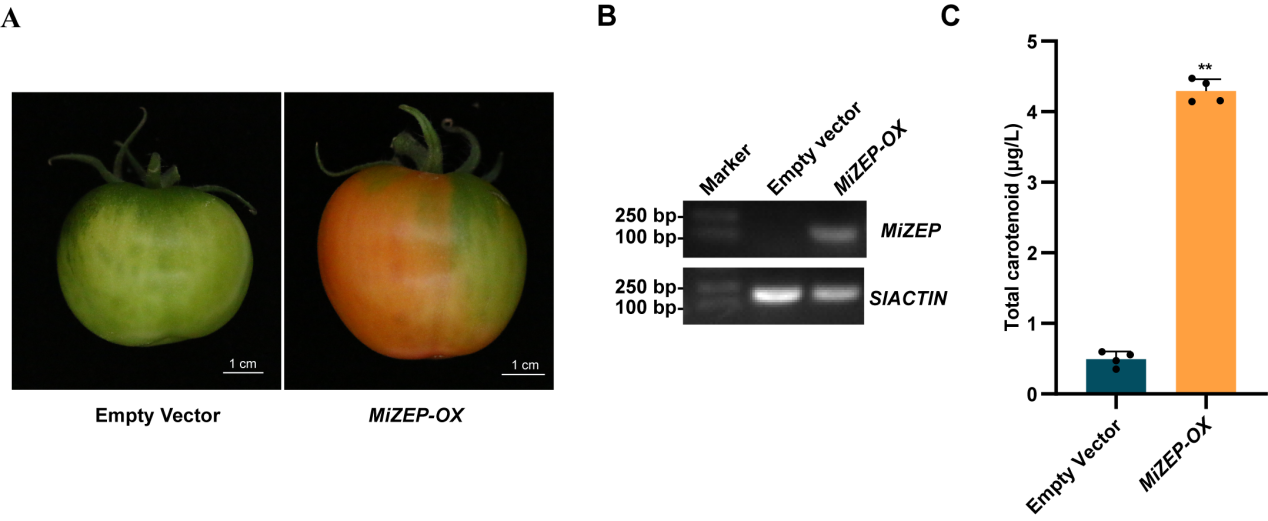


Fig. S2 Functional analysis of MiZEP in ‘Alisa Craig’ tomato fruit. (A) Representative images of the transient overexpression of *MiZEP* in tomato fruit. *MiZEP* was cloned into the pCAMBIA1301 vector with a *35S* promoter, and the empty vector was used as the control. The plasmids were transformed into *A. tumefaciens* GV3101. Selected green-mature stage tomato fruits were subjected to shoulder injections while still on the tree. The phenotypes were then examined after 7 d. Scale bars, 1 cm. (B) Results of the RT-PCR amplification of the *MiZEP*-specific fragments in ‘Alisa Craig’ tomato fruit and (C) total carotenoid content in tomato fruit transiently overexpressing *MiZEP*. Error bars represent the standard deviation of three biological replicates. Asterisks indicate statistically significant differences (**p* < 0.05 and ***p* < 0.01), as determined by a Student’s *t*-test.


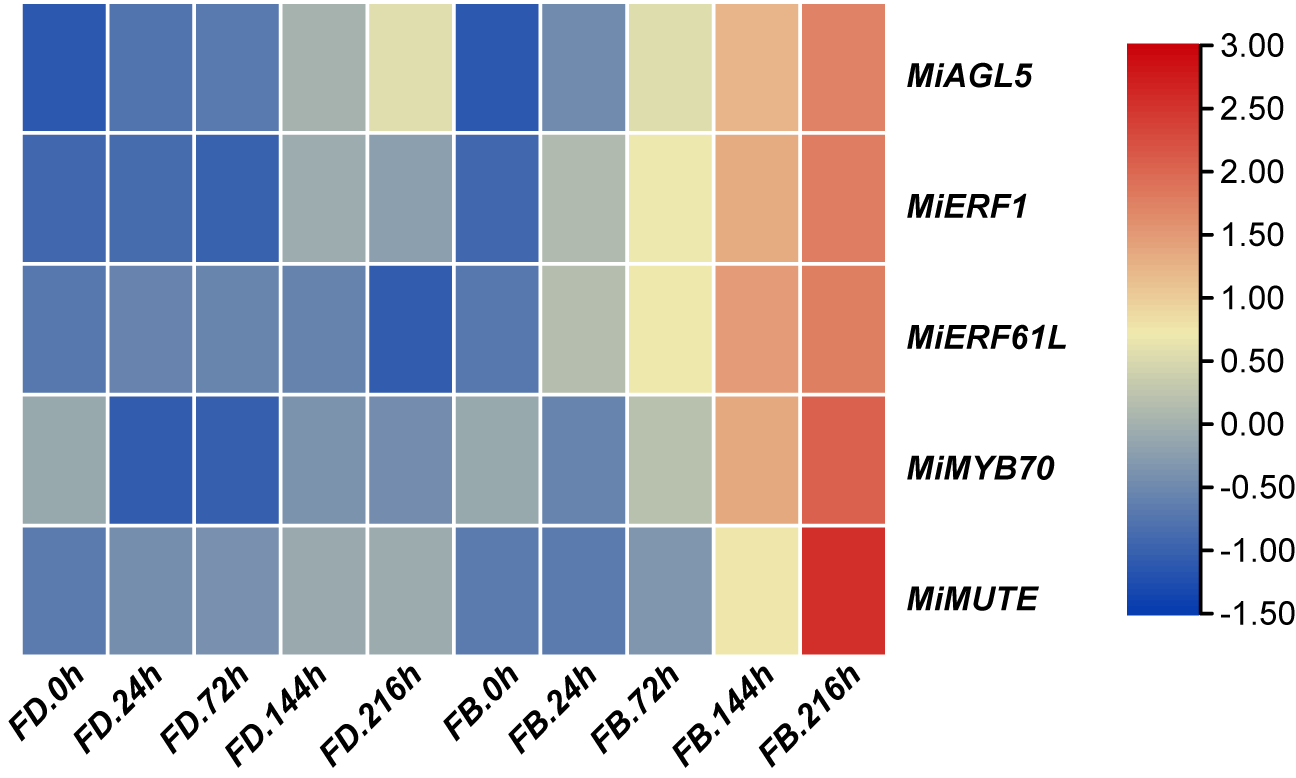


Fig. S3 Heat map showing the differential expression patterns of five candidate genes in control and blue light-treated mango flesh, as listed in Table S4. The FPKM values were used to generate a heatmap with hierarchical clustering analysis.


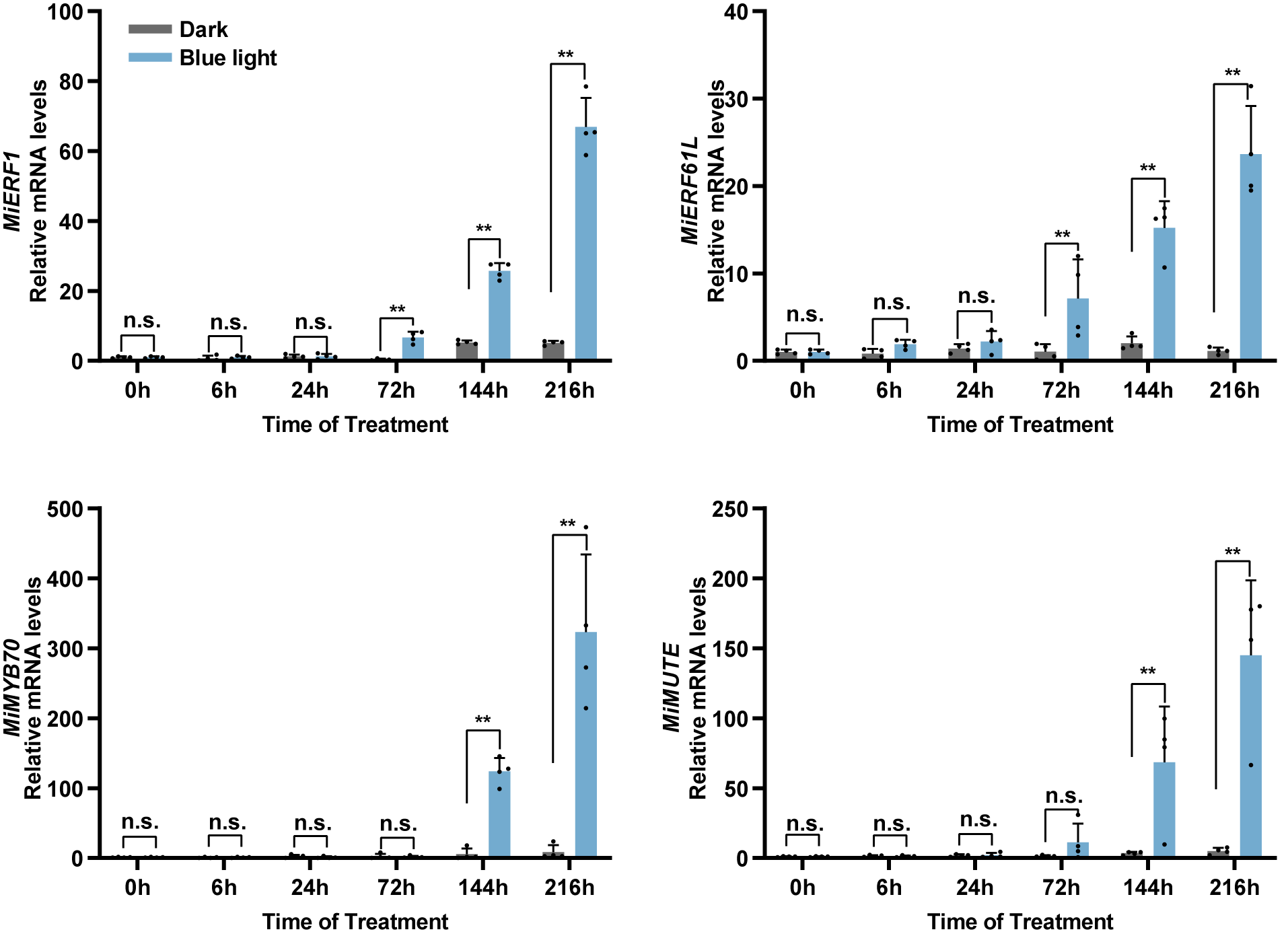


Fig. S4 Expression patterns of candidate transcription factor genes in mango flesh under blue light treatment. ***MiActin* was used as an internal reference. All the above data were expressed as standard deviation of three biological replicates.** Asterisks indicate significantly different values (**p* < 0.05 and ***p* < 0.01) as determined by a Student’s *t* test. n.s., no significant difference.


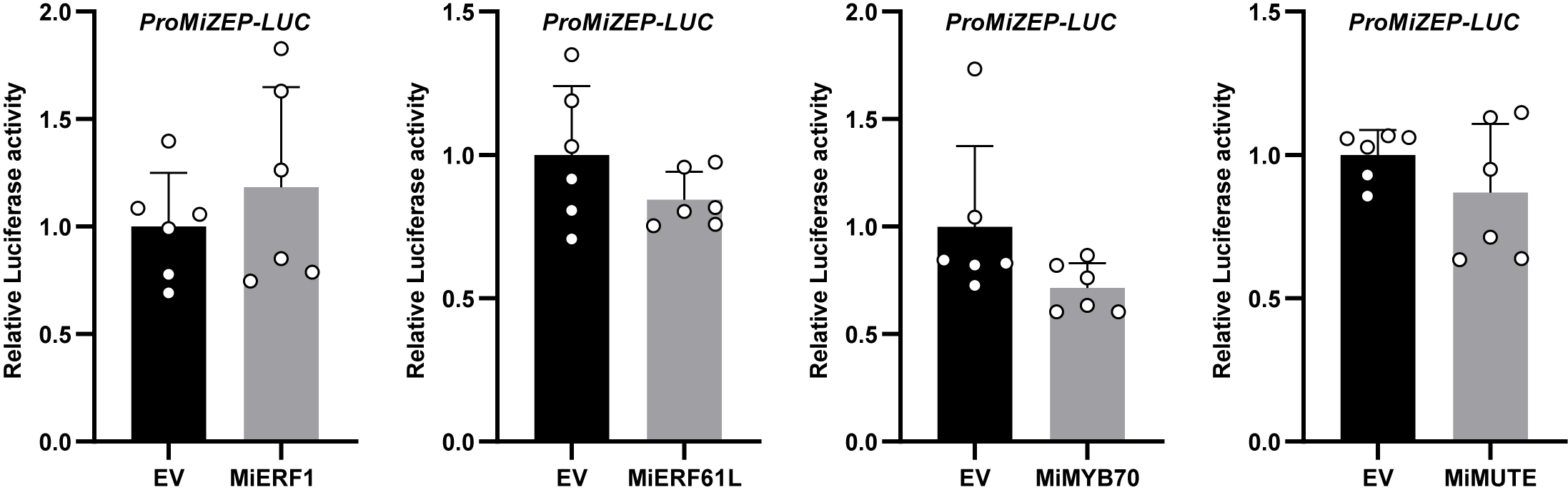


Fig. S5 Assay of transcriptional activation effect of MiERF1, MiERF61L, MiMYB70, and MiMUTE on the *MiZEP* promoter using dual-luciferase assay. **The empty SK vector was used as negative control (EV). All the above data were expressed as standard deviation of three biological replicates.** Asterisks indicate significantly different values (**p* < 0.05 and ***p* < 0.01) as determined by a Student’s *t* test.


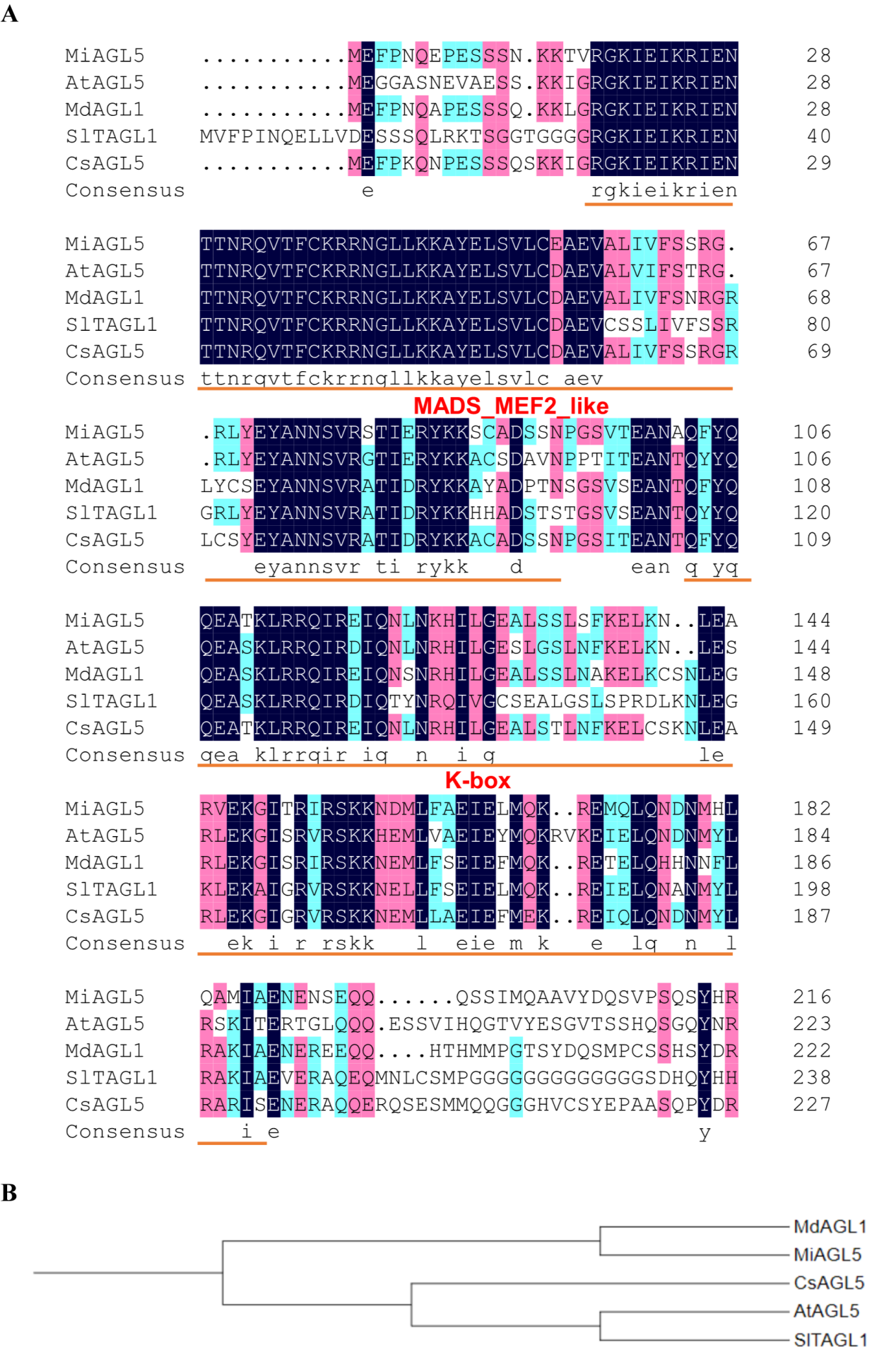


Fig. S6 Multiple sequence alignment of the **MiAGL5** protein in mango and other plants. Sequence data for AtAGL5 (***Arabidopsis thaliana*,** NP_850377.1), MdAGL5 (*Malus domestica*, NP_001280918.1), SlTAGL1 (*Solanum lycopersicum*, NP_001300859.1), and CsAGL5 (*Citrus sinensis*, KAH9770846.1) were downloaded from the NCBI BioProject database ([https://www.ncbi.nlm.nih.gov/bioproject](https://www.ncbi.nlm.nih.gov/bioproject)). The MADS and K-box domains are represented with lines.


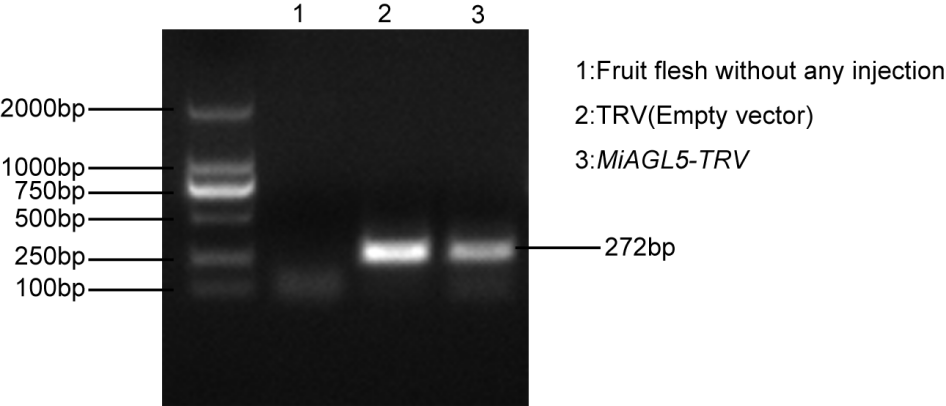


Fig. S7 PCR results showed that fragment of pTRV2 was expressed in the flesh which was injected with empty vector as well as MiAGL5-TRV, while pTRV2 was not expressed in the flesh without any injection.


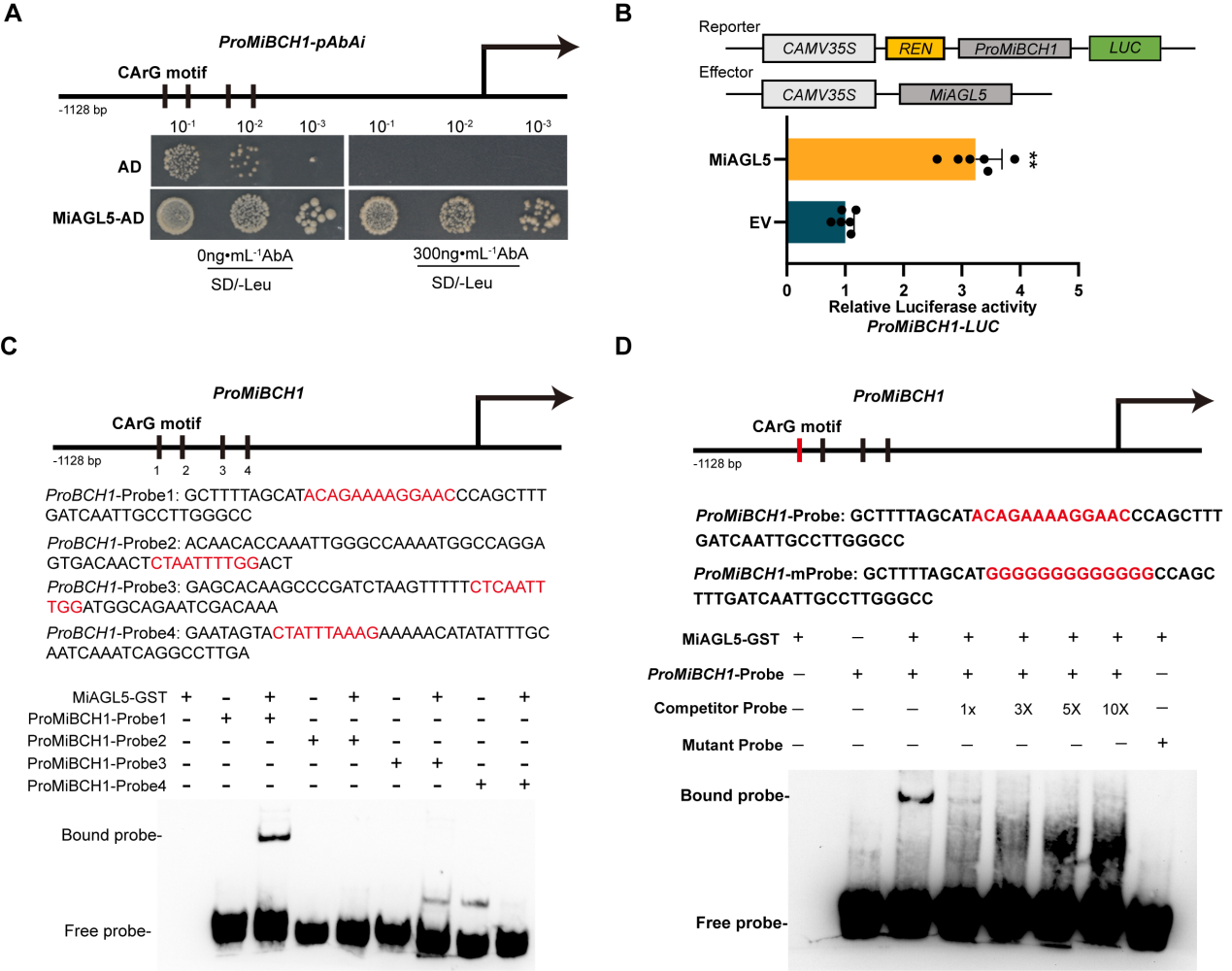


Fig. S8 MiAGL5 directly binds to the promoter of *MiBCH1* and activates its expression. (A) A Y1H assay showed that MiAGL5 directly binds to the promoter of *MiBCH1* containing GArG motifs*.* AD-empty (pGADT7) was used as the negative control. (B) A dual-luciferase assay revealed that MiAGL5 activate the promoter of *MiBCH1* in vivo. The promoter of *MiBCH1* was cloned into the pGreenII 0800-*LUC* vector, and the full-length CDS of MiAGL5 was cloned into the pGreenII 0029 62-SK vector. The empty vector of pGreenII 0029 62-SK was used as control. The relative luciferase activity was analyzed. (C) The binding of the MiAGL5 protein to four GArG motifs on the promoter of *MiBCH1.* An EMSA was performed using four biotin-labeled *MiBCH1* promoter fragments, each containing a GArG motif (ACAGAAAAGGAAC, CTAATTTTGG, CTCAATTTGG, CTATTTAAAG). (D) An EMSA showed that MiAGL5 binds to the GArG motif (ACAGAAAAGGAAC) on the promoter of *MiBCH1.* An EMSA was performed using a biotin-labeled *MiBCH1* promoter fragment, whereas the competitor was an unlabeled and identical *MiBCH1* promoter fragment probe (1-, 3-, 5-, 10-fold molar excess). The mutated probe was identical to the labeled probe, but the ACAGAAAAGGAAC was mutated to GGGGGGGGGGGGG. The MiAGL5-GST protein used for the EMSA was purified. Error bars represent the standard deviation of 3 biological replicates. Asterisks indicate significantly different values (**p* < 0.05 and ***p* < 0.01).


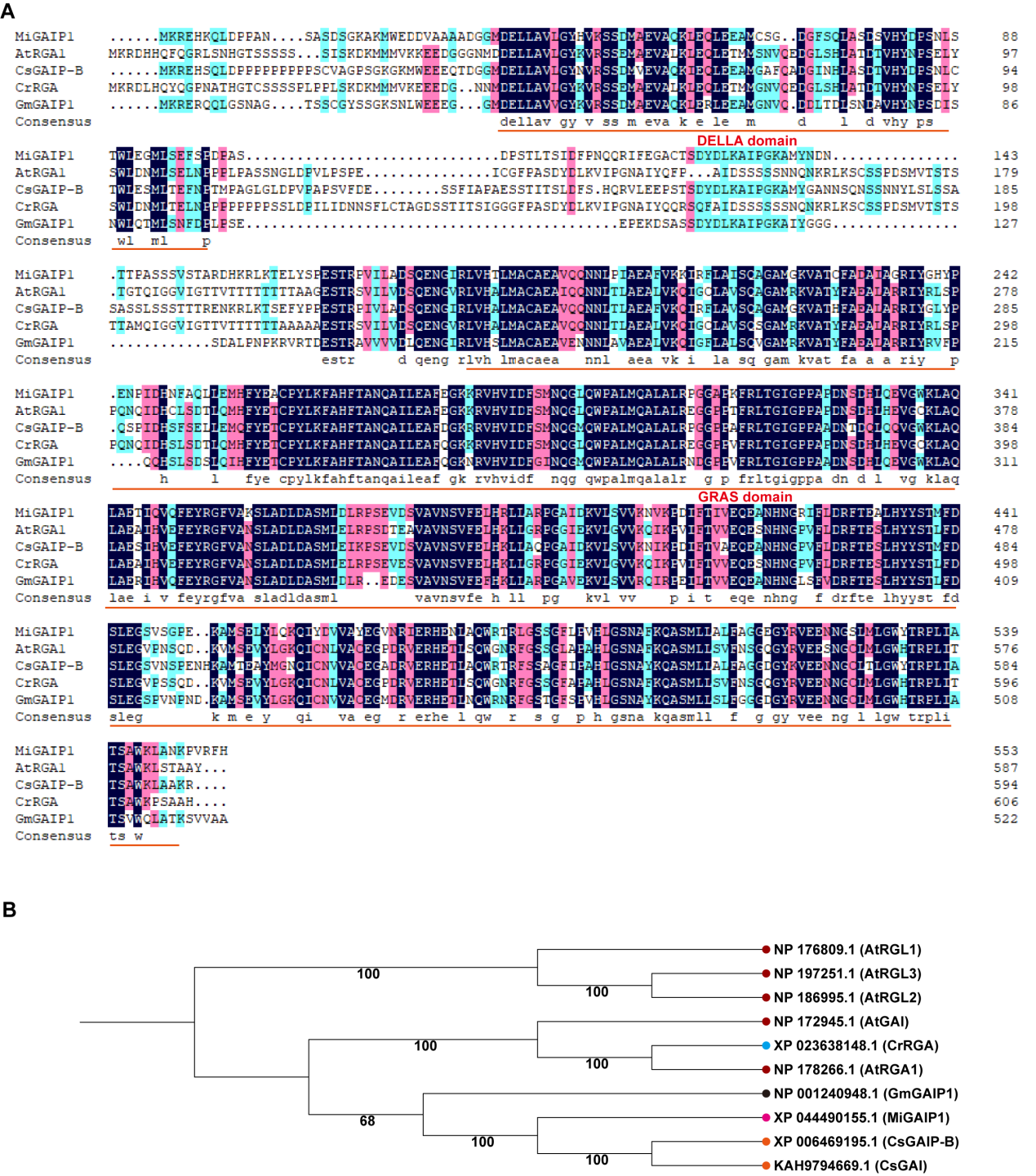


Fig. S9 Multiple sequence alignment and phylogenetic tree analysis of MiGAIP1. (A) Multiple sequence alignment of MiGAIP1 protein in mango and other plants. Sequence data for MiGAIP1 (*Mangifera indica*, XP_044490155.1), AtRGA1 (***Arabidopsis thaliana*,** NP_178266.1), CsGAIP-B (*Citrus sinensis*, XP_006469195.1), CrRGA (*Capsella rubella*, XP_023638148.1), and GmGAIP1 (*Glycine max*, NP_001240948.1) were downloaded from the NCBI BioProject database ([https://www.ncbi.nlm.nih.gov/bioproject](https://www.ncbi.nlm.nih.gov/bioproject)). The DELLA and GRAS domains are represented with lines. (B) Phylogenetic tree of MiGAIP1 in mango and other plants. Different plants are highlighted by distinct colors: *Arabidopsis thaliana* in rose red dots, *Capsella rubella* in blue dots, *Glycine max* in black dots, *Citrus sinensis* in orange dots, and *Mangifera indica* in magenta dots.


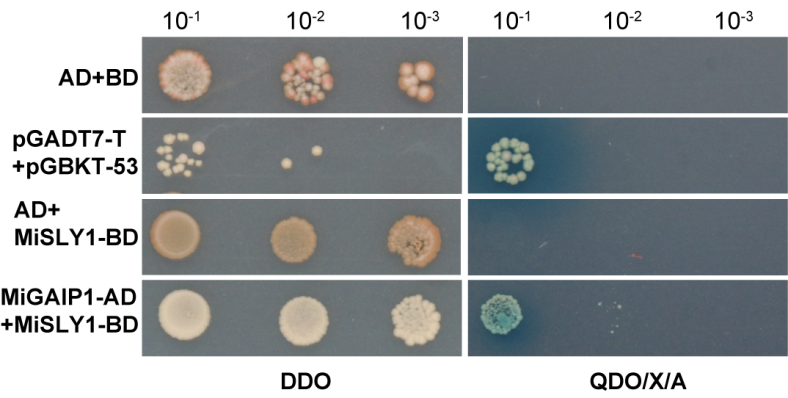


Fig. S10 Yeast 2-hybrid assays showing the interactions between MiGAIP1 and MiSLY1. Yeast cells co-expressing the proteins were grown on DDO (-Trp/-Leu) medium. Yeast grown on the QDO/X/A (-Ade/-His/-Leu/-Trp/X-*α*-gal/Aba) medium showed interactions. AD, pGADT7; BD, pGBKT7. The negative control contained empty AD and empty BD. The positive control contained pGADT7-T and pGBKT7-53.


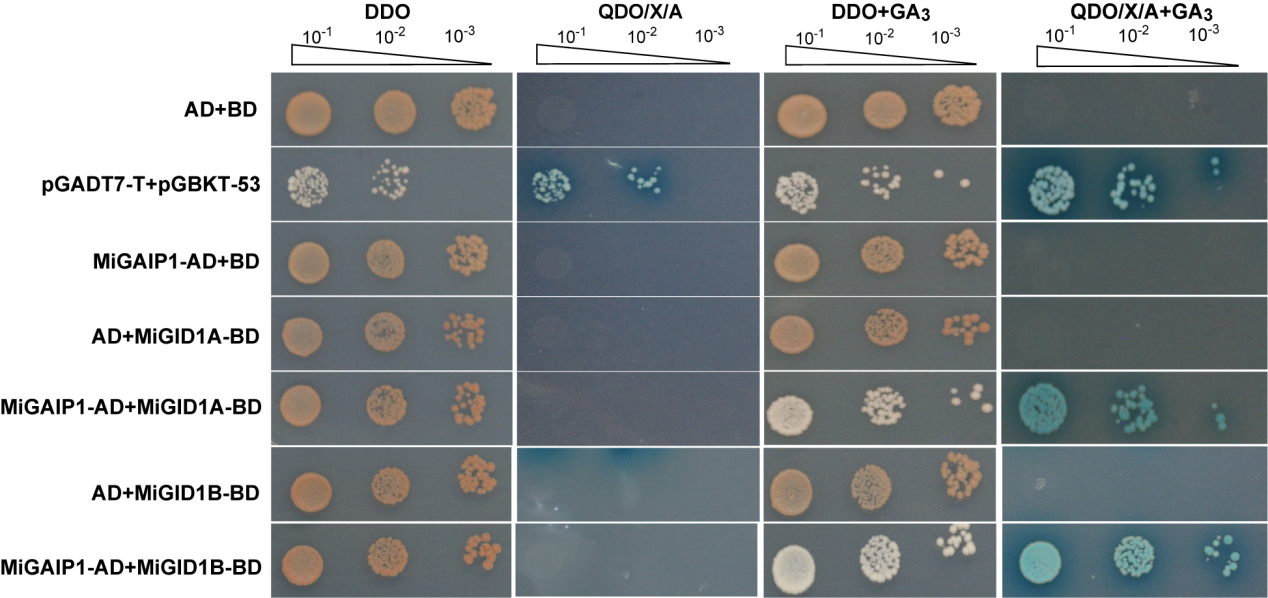


Fig. S11 Yeast 2-hybrid assays showing interactions between MiGAIP1 and GA-dependent MiGIDs (MiGID1A and MiGID1B). Yeast cells co-expressing the proteins were grown on DDO (-Trp/-Leu) and DDO + GA_3_ (100 μM) medium. Yeast grown on the QDO/X/A (-Ade/-His/-Leu/-Trp/X-*α*-gal/Aba) and QDO/X/A + GA_3_ (100 μM ) medium showed interactions.

AD, pGADT7; BD, pGBKT7. The negative control contained empty AD and empty BD. The positive control contained pGADT7-T and pGBKT7-53.


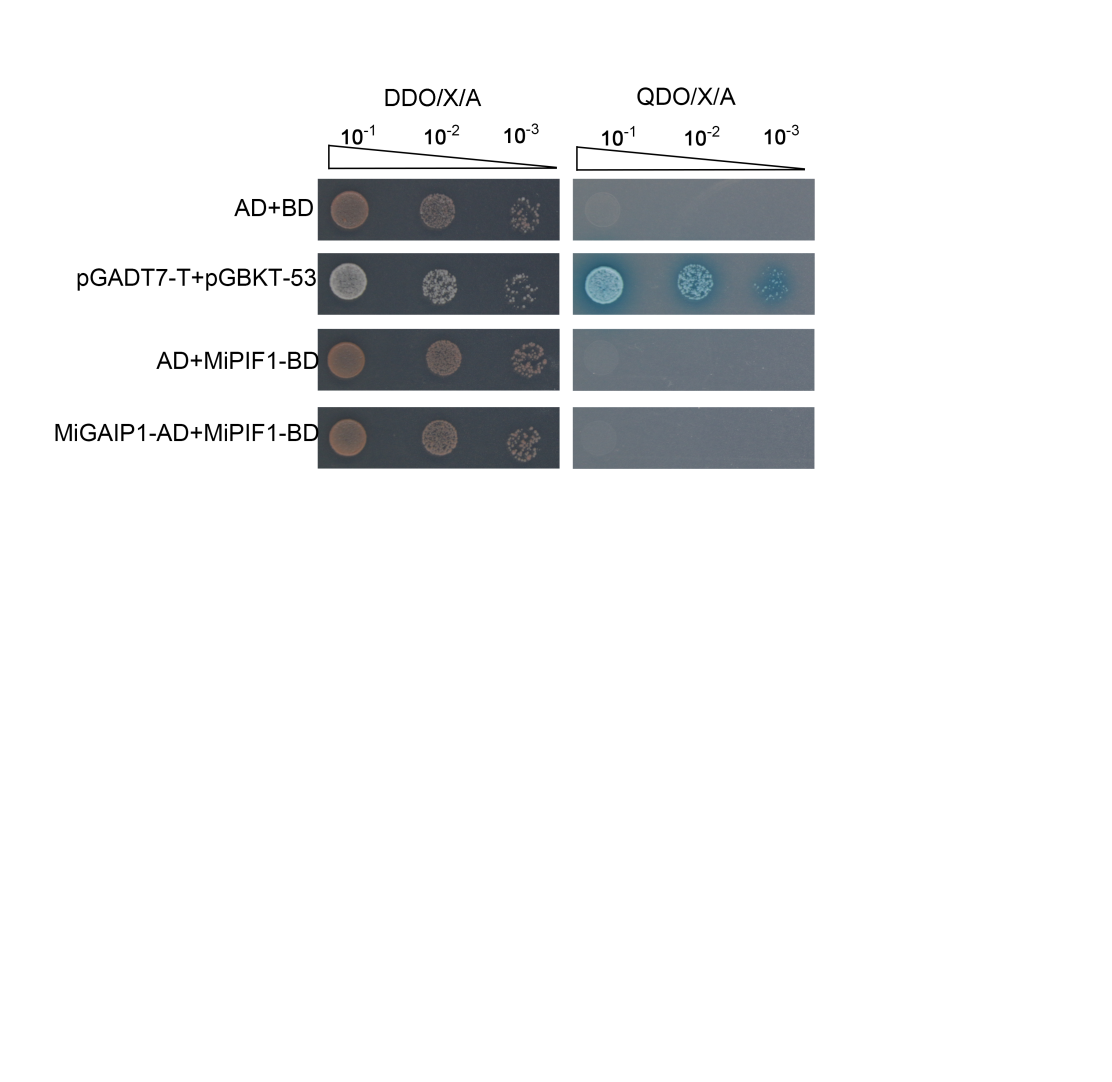


Fig. S12 Yeast 2-hybrid assays demonstrating that MiGAIP1 can not interact with MiPIF1 in yeast. Yeast cells co-expressing the proteins were grown on DDO (-Trp/-Leu) medium. Yeast grown on the QDO/X/A (-Ade/-His/-Leu/-Trp/X-*α*-gal/Aba) medium showed interactions. AD, pGADT7; BD, pGBKT7. The negative control contained empty AD and empty BD. The positive control contained pGADT7-T and pGBKT7-53.


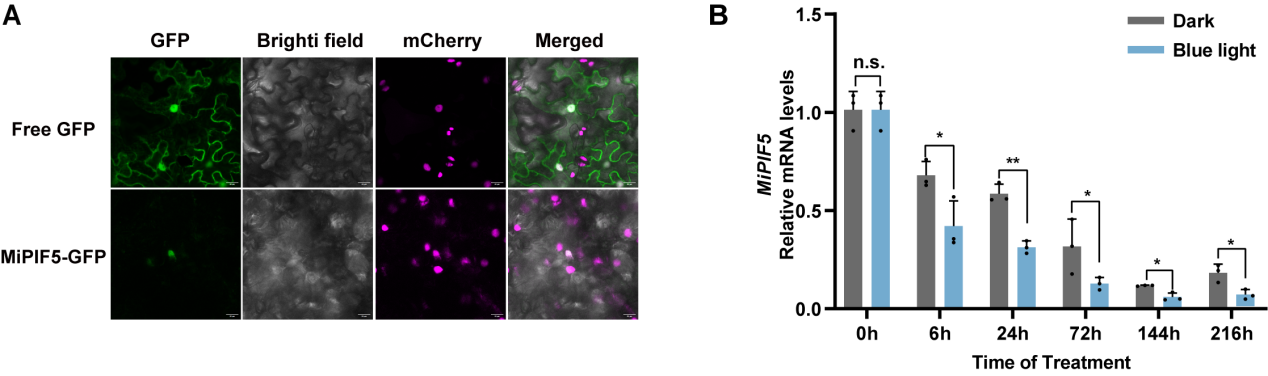


Fig. S13 Identification of MiPIF5. (A) S**ubcellular localization of MiPIF5. MiPIF5-GFP and GFP were transiently transformed into *N. benthamiana* leaves, and localization was observed under confocal laser microscopy. (B)** Expression patterns of *MiPIF5* in mango flesh under blue light treatment. ***MiActin* was used as an internal reference. All the above data were expressed as standard deviation of three biological replicates.** Asterisks indicate significantly different values (**p* < 0.05 and ***p* < 0.01) as determined by a Student’s *t* test. n.s., no significant difference.


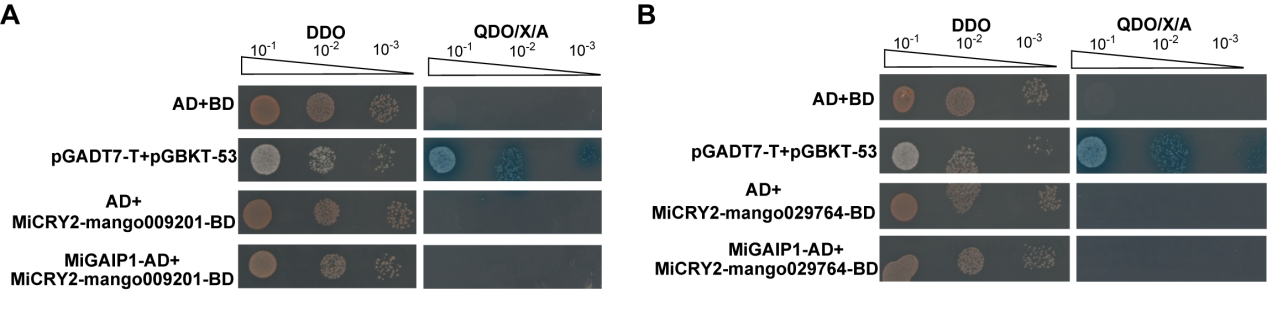


Fig. S14 Yeast two-hybrid assays indicated that MiGAIP1 did not interact with either MiCRY1-mango009201 or MiCRY2-mango029764. Yeast cells co-expressing the proteins were grown on DDO (-Trp/-Leu) medium. Yeast grown on the QDO/X/A (-Ade/-His/-Leu/-Trp/X-*α*-gal/Aba) medium demonstrated interactions. AD, pGADT7; BD, pGBKT7. The negative control contained empty AD and empty BD. The positive control contained pGADT7-T and pGBKT7-53.


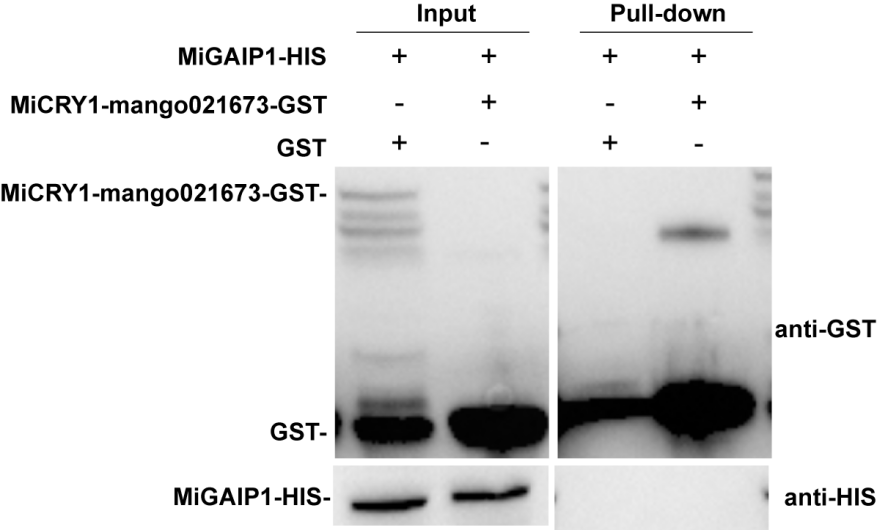


Fig. S15 Pull-down assay confirming that MiGAIP1 does not interact with MiCRY1-mango021673 in vitro. Immobilized GST and MiCRY1-mango021673-GST were used to pull down MiGAIP1-His, and immunoprecipitated fractions were detected using anti-His antibody. The bait proteins were probed with anti-GST antibody.

**
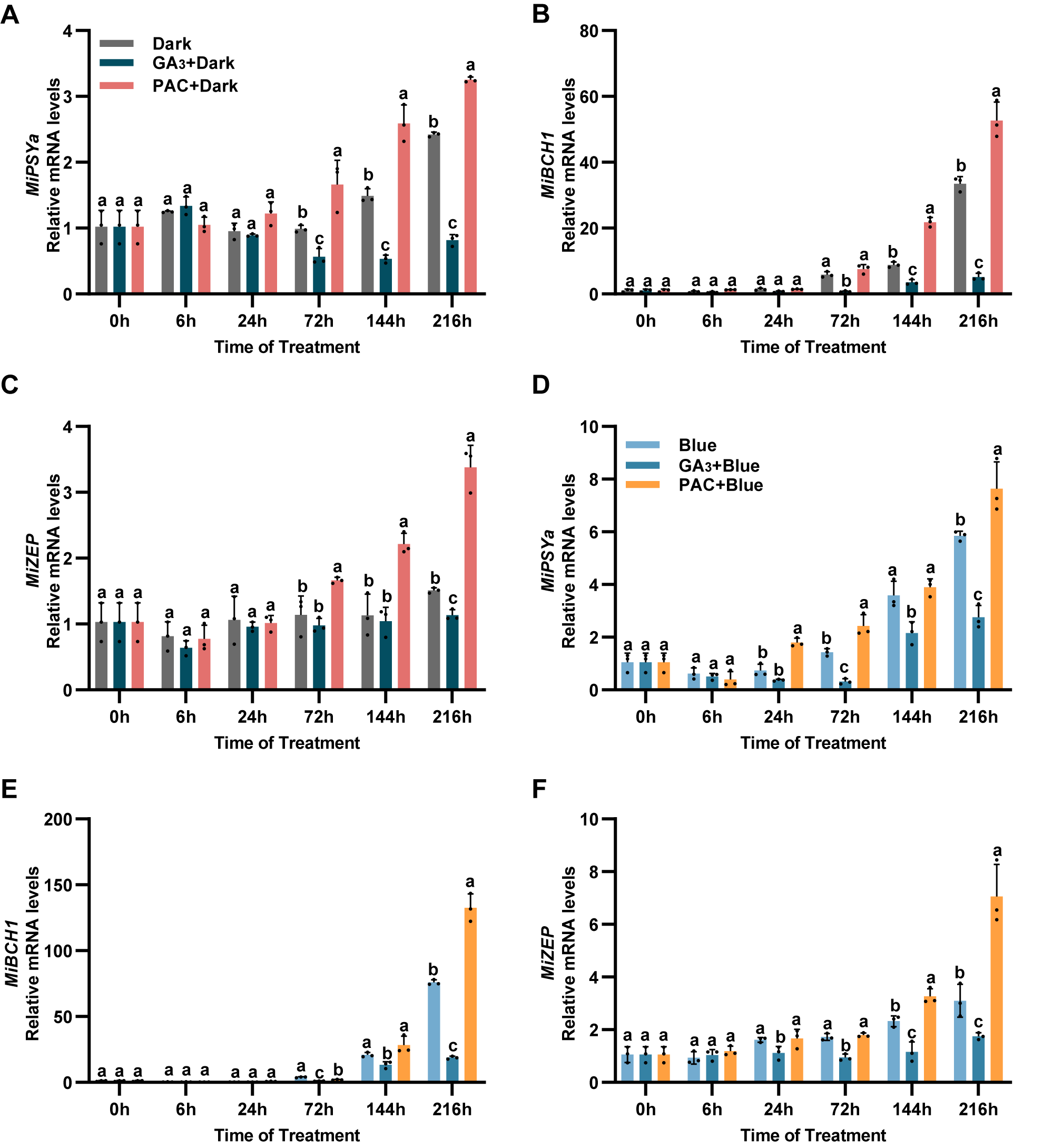
**

Fig. S16 Expression patterns of *MiPSYa*, *MiBCH1*, and *MiZEP* in mango flesh under treatment. ***MiActin* was used as an internal reference.** Number under the X axes in (A) to (F) indicate the time after treatment. **Error bars represent the standard deviation of three biological replicates.**

Error bars represent the standard deviation of three biological replicates. Different lowercase letters above the error bars indicate significant differences, as determined by one-way ANOVA (Tukey) (*p* < 0.05).

Supplemental Table

Table S1 Carotenoid compound content in the mango flesh of control and overexpressing-*MiAGL5*

| Index | EV-1 | EV-2 | EV-3 | AGL5-OX-1 | AGL5-OX-2 | AGL5-OX-3 |
| --- | --- | --- | --- | --- | --- | --- |
| α-carotene | 0.21708779 | 0.150772573 | 0.218988445 | 0.348102751 | 0.213585252 | 0.325185934 |
| ε-carotene | N/A | N/A | N/A | N/A | N/A | N/A |
| lycopene | N/A | N/A | N/A | N/A | N/A | N/A |
| γ-carotene | 0.0870052472 | 0.11868989 | 0.0867834989 | N/A | N/A | N/A |
| phytofluene | 0.100848638 | 0.0754918919 | 0.0831735252 | 0.100120955 | 0.0713733958 | 0.0826936136 |
| β-carotene | 1.98690414 | 1.34074474 | 1.83140077 | 2.34470065 | 2.91426971 | 2.83930881 |
| (E/Z)-phytoene | 1.72478103 | 1.15560761 | 1.4016197 | 1.1470894 | 1.00646975 | 1.29506871 |
| lutein dilaurate | 0.0702096872 | 0.0606736737 | 0.0706326779 | 0.0899095874 | 0.0675115095 | 0.0932382781 |
| lutein dimyristate | 0.196738042 | 0.148104505 | 0.154309548 | 0.233539644 | 0.180683846 | 0.260582053 |
| violaxanthin dibutyrate | 0.290137235 | 0.198069269 | 0.228814109 | 0.312625405 | 0.232432267 | 0.344490703 |
| violaxanthin-myristate-caprate | 1.06695257 | 1.23608408 | 0.851011555 | 0.676500809 | 0.580875942 | 0.729173403 |
| β-cryptoxanthin myristate | 0.0549836529 | 0.0753445445 | 0.056200892 | 0.0531223706 | 0.044026482 | 0.0466475344 |
| zeaxanthin | 0.0944337033 | 0.0625107107 | 0.0844625988 | 0.10253661 | 0.1127406804 | 0.122985449 |
| violaxanthin | 0.149721493 | 0.115181381 | 0.114492195 | 0.232194579 | 0.264070483 | 0.282252627 |
| neoxanthin | 0.0136995358 | 0.00405171171 | 0.0399260085 | 0.0104176173 | 0.00788761459 | 0.00999223929 |
| lutein | 0.248391524 | 0.165555355 | 1.07074802 | 0.380192152 | 0.340853534 | 0.48505861 |
| β-cryptoxanthin | 0.356827447 | 0.302154154 | 0.306555848 | 0.552392799 | 0.576339377 | 0.522002829 |
| 8'-apo-beta-carotenal | 0.00504361251 | 0.00439835836 | 0.00466943037 | 0.0049839199 | 0.00414886942 | 0.00493005255 |
| α-cryptoxanthin | 0.0487880928 | 0.0322068068 | 0.0421921751 | 0.0461110437 | 0.0401780403 | 0.0526400566 |
| canthaxanthin | 0.000216992936 | 0.000185663063 | 0.000146725725 | 0.000186666869 | 0.000167156447 | 0.000190107316 |
| echinenone | 0.00159476287 | 0.00108785986 | 0.00143173525 | 0.00279370955 | 0.00217086983 | 0.00225109135 |
| neochrome palmitate | 0.113590111 | 0.0803807808 | 0.0830320292 | 0.260978964 | 0.216781422 | 0.244438157 |
| rubixanthin laurate | 0.03890111 | 0.0382964965 | 0.0363213055 | 0.0406620146 | 0.0410853534 | 0.0548940986 |
| rubixanthin palmitate | 0.101547931 | 0.0856652653 | 0.0680735861 | 0.111844862 | 0.103734365 | 0.140614794 |
| β-cryptoxanthin laurate | 0.0388910192 | 0.038803003 | 0.0362994121 | 0.0318824838 | 0.0272578937 | 0.0368896524 |
| β-cryptoxanthin palmitate | 0.0764183653 | 0.0992424424 | 0.0756399757 | 0.0865928398 | 0.0631949481 | 0.0831333872 |
| lutein myristate | N/A | N/A | N/A | 0.12697856 | 0.117023834 | 0.139832053 |
| violaxanthin-myristate-laurate | N/A | N/A | N/A | 0.42484021 | 0.224691383 | 0.287336297 |
| lutein dipalmitate | 0.139331382 | 0.138328529 | 0.139935536 | 0.148553398 | 0.140869016 | 0.151509297 |

Table S2 Primers for qPCR analysis

| Gene Name | Forward primer (5'to 3') | Reverse primer (5'to 3') |
| --- | --- | --- |
| *MiPSYa* | CCAGGGTCGTCCATTTGACA | ACTCTCTGTCGTTGCCTGTG |
| *MiZDS* | CGCTCTGGCTCTTGCTCTAA | CTAAGATGGAACCTGCCCCC |
| *MiBCH1* | TGGCGACTGGAGTTTCATCC | CTCCACCCTCCATTTGCCAT |
| *MiLCYb* | CGTTGTGGACTTGGCAGTTG | TCCTGGCCACCATATACCCA |
| *MiBCH2* | TCCGTTTGGTTACCGCAACT | AACTCCATACCCACAGCAGC |
| *MiZEP* | AAGGTGGCTTCTCGAACCTG | CACCAACAGGCTCCCTTCTT |
| *MiActin* | CCAAGCAGCATGAAGATCAA | ATCTGCTGGAAGGTGCTGAG |
| *MiAGL5* | ACCTTCTGTAAGCGTCGCAA | GTTTGGTGGCTTCTTGCTGG |
| *MiERF1* | CCAACCAGGAAAACGGTTCG | GGGGCACGATTATGCTAGGT |
| *MiERF61L* | CAGCCCTGTATCCAAGCCAT | GGCGTCAACTGAGCTCTTCA |
| *MiMYB70* | GAGACCGATCGGATCAAGGG | TCAACTCCGGCCAAACTACC |
| *MiMUTE* | CTGCGTTCTTTAACGCCATGT | GACACTCCAGCCCTATCTTGATG |
| *SlACTIN* | CCTCAGCACATTCCAGCAG | CCACCAAACTTCTCCATCCC |
| *SlPSY* | CGGCAGCCTTAGATAGGTGG | AAGCACCATCGAGCATGTCA |
| *SlZEP* | GTTCTCCAACTGCAAAGCCG | CACACCAGGTACGAGCCTTT |
| *MiPDS* | GAAGTTCTGCCAGCTCCTGT | GATCTTTGCAGGGGACGACA |
| *MiLCYb* | CGTTGTGGACTTGGCAGTTG | TCCTGGCCACCATATACCCA |
| *MiGAIP1* | GATGTGGGAGGATGACGTGG | TTCTGAGCAACTTCCGCCAT |
| *MiPIF5* | AAATCTGGGTCTTCCCGCAG | TTCCCATCCACATAACCTGAAGT |
| *MiCRY1* | GGGGAGGGTGTCTAGATGGT | CCAGGCGACCATGCTCTTG |

Table S3 Primers used for constructing vectors and EMSA

| Gene | Forward primer (5'to 3') | Reverse primer (5'to 3') |
| --- | --- | --- |
| MiAGL5-pCAMBIA1300 | AGCTCGGTACCCGGG ATGGAGTTTCCAAACCAAGAAC | CATGTCGACTCTAGA ATGATTGGGTTCCAGG |
| MiGAIP1-pCAMBIA1205 | CGGGCTGCAGGAATTCATGAAGAGAGAACACAAACAACTCG | CCCCCTCGAGGTCGACTCAGTGAAACCGAACCGG |
| MiPIF5-pCAMBIA1300 | AGCTCGGTACCCGGGATGAATCCATGTATACCAGATTG | CATGTCGACTCTAGAACCTGTCTTGCCACTTAGAGAG |
| MiCRY1-pCAMBIA1300 | AGCTCGGTACCCGGGATGTCAGGTGGTGGGTGTAGTATAG | CATGTCGACTCTAGACCCAGTTTGAGAAAGCCG |
| MiAGL5-BD | ATGGCCATGGAGGCCGAATTCATGGAGTTTCCAAACCAAGAAC | CCGCTGCAGGTCGACGGATCCTCAATGATTGGGTTCCAGG |
| MiAGL5-VP16-BD | GGAGGACCTGCATATGATGGAGTTTCCAAACCAAGAAC | AACCCAGCATGAATTCATGATTGGGTTCCAGG |
| MiGAIP1-BD | ATGGCCATGGAGGCCGAATTCATGAAGAGAGAACACAAACAACTCG | CCGCTGCAGGTCGACGGATCCTCAGTGAAACCGAACCGG |
| MiGAIP1-VP16-BD | GGAGGACCTGCATATGATGAAGAGAGAACACAAACAACTCG | AACCCAGCATGAATTCGTGAAACCGAACCGG |
| MiAGL5-AD | GCCATGGAGGCCAGTGAATTCATGGAGTTTCCAAACCAAGAAC | CAGCTCGAGCTCGATGGATCCTCAATGATTGGGTTCCAGG |
| MiGAIP1-AD | GCCATGGAGGCCAGTGAATTCATGAAGAGAGAACACAAACAACTCG | CAGCTCGAGCTCGATGGATCCTCAGTGAAACCGAACCGG |
| MiGAIP1N-AD | GCCATGGAGGCCAGTGAATTCATGAAGAGAGAACACAAACAACTCG | CAGCTCGAGCTCGATGGATCCGGATGAAGAAGCAGGGGTAG |
| MiGAIP1C-AD | GCCATGGAGGCCAGTGAATTCGTTTCCACAGCTAGAGATCACAAAC | CAGCTCGAGCTCGATGGATCCTCAGTGAAACCGAACCGG |
| MiPIF5-AD | GCCATGGAGGCCAGTGAATTCATGAATCCATGTATCCCAGATTG | CAGCTCGAGCTCGATGGATCCTTATGTACCTGTCTTGCCACTTAGAG |
| ProMiBCH1-pAbAi | TGAAAAGCTTGAATTCGAGCTCGATAAGATCGTGACCAAAAC | AGCACATGCCTCGAGGTCGACGTTTGGGGACCAACTCAG |
| ProMiZEP-pAbAi | TGAAAAGCTTGAATTCGAGCTCGATATGATACCAAAGAATC | AGCACATGCCTCGAGGTCGACATATTATAAAACTTTAAAAGGC |
| ProMiZEPS1-pAbAi | TGAAAAGCTTGAATTCGAGCTCGATATGATACCAAAGAATC | AGCACATGCCTCGAGGTCGACAGATTAATTAATGCTACCTTTG |
| ProMiZEPS2-pAbAi | TGAAAAGCTTGAATTCGAGCTCCAAAGGTAGCATTAATTAATCT | AGCACATGCCTCGAGGTCGACATTAATTTAAAATCATTCAATC |
| ProMiZEPS3-pAbAi | TGAAAAGCTTGAATTCGAGCTCGATTGAATGATTTTAAATTAAT | AGCACATGCCTCGAGGTCGACATATTATAAAACTTTAAAAGGC |
| ProMiAGL5-pAbAi | TGAAAAGCTTGAATTCGAGCTCCGACGCATCCTACTTTAGAGCAA | AGCACATGCCTCGAGGTCGACCACTTCCATACCCAACATTAGCA |
| MiSLY1-BD | ATGGCCATGGAGGCCGAATTCATGAAGCGTACGGTTTTTGATTC | CCGCTGCAGGTCGACGGATCCTCAACTACCCCGATTACTAGAATTC |
| MiGID1A-BD | ATGGCCATGGAGGCCGAATTCATGGCTGCAAGTAATGAAGTTAATC | CCGCTGCAGGTCGACGGATCCTTAACAGTTAGAACACACAAAATTACTTATC |
| MiGID1B-BD | ATGGCCATGGAGGCCGAATTCATGGCTGGTAGTAATGAAGTCAAC | CCGCTGCAGGTCGACGGATCCTTAACAGTTAGGATTCACAAACTTC |
| MiCRY2-mango009201-BD | ATGGCCATGGAGGCCGAATTCATGACGACCATGGCAAGC | CCGCTGCAGGTCGACGGATCCTTATTGCCTGTTTACCCTAAAATG |
| MiCRY2-mango029764-BD | ATGGCCATGGAGGCCGAATTCATGAGATTTTCACACTCTCTCTCTG | CCGCTGCAGGTCGACGGATCCTCAACCAGTCAGCTTTTGTCTC |
| MiERF1-SK | GCGGCCGCTCTAGAACTAGTGATGGCGGGGCCTG | GGTCGACGGTATCGATAAGCTCTATAATAGAACTCCTTTCGTTTC |
| MiERF61L-SK | GCGGCCGCTCTAGAACTAGTGATGCAAGGAATTCAAGAAAATAGTT | GGTCGACGGTATCGATAAGCTTTAATTAGCAAGAACTTCCCAAATT |
| MiMYB70-SK | GCGGCCGCTCTAGAACTAGTGATGAATCGGGTTTTAGAGACCG | GGTCGACGGTATCGATAAGCTTTAGTAACCCTTAATCCCATTA |
| MiMUTE-SK | GCGGCCGCTCTAGAACTAGTGATGTCTCACATAGCTGTGGAG | GGTCGACGGTATCGATAAGCTTTACATCTCGTGGGAGTAAAC |
| MiAGL5-SK | GCGGCCGCTCTAGAACTAGTGATGGAGTTTCCAAACCAAGAAC | GGTCGACGGTATCGATAAGCTTCAATGATTGGGTTCCAGG |
| MiGAIP1-SK | GCGGCCGCTCTAGAACTAGTGATGAAGAGAGAACACAAACAACTCG | GGTCGACGGTATCGATAAGCTTCAGTGAAACCGAACCGG |
| MiPIF5-SK | GCGGCCGCTCTAGAACTAGTG ATGAATCCATGTATACCAGATTG | GGTCGACGGTATCGATAAGCT TTAACCTGTCTTGCCACTTAGAGAG |
| ProMiZEP-1-LUC | TCGAGGTCGACGGTATCGATACAAAGAATCATATTTTTGGTTGATC | CCGCTCTAGAACTAGTGGATCATCTTTTAGTGCTCGTGTGTGGC |
| ProMiZEP-2-LUC | TCGAGGTCGACGGTATCGATATAACACTTAATTATGTGATGACATATGTCAG | CCGCTCTAGAACTAGTGGATCATCTTTTAGTGCTCGTGTGTGGC |
| ProMiZEP-3-LUC | TCGAGGTCGACGGTATCGATAATACTTAAAATGGATATAC | CCGCTCTAGAACTAGTGGATCATCTTTTAGTGCTCGTGTGTGGC |
| ProMiZEP-4-LUC | TCGAGGTCGACGGTATCGATACACTTCTTCACCCAACCAAAAG | CCGCTCTAGAACTAGTGGATCATCTTTTAGTGCTCGTGTGTGGC |
| ProMiZEP-5-LUC | TCGAGGTCGACGGTATCGATAGAATACGAATCTTCATACAGCGTAAG | CCGCTCTAGAACTAGTGGATCATCTTTTAGTGCTCGTGTGTGGC |
| ProMiBCH1-LUC | TCGAGGTCGACGGTATCGATACAGCCTTGAATACGTTACAATTGCG | CCGCTCTAGAACTAGTGGATCTGAAGATTCGAGGGATTGGATC |
| ProMiAGL5-LUC | GGTATCGATAAGCTTGCAAAAGACCCCCATTATCTTCC | TTTGGCGTCTTCCATCTTATTAGAAGAACTTTCTGGTTCTTGG |
| MiAGL5-PGEX4T | GATCTGGTTCCGCGTGGATCCATGGAGTTTCCAAACCAAGAAC | AGTCAGTCACGATGCGGCCGCTCAATGATTGGGTTCCAGG |
| ProMiZEP-Probe | CACTTCTTCACCCAACCAAAAGCTTAATTTTGTTAATGAATACGAATCTTCATACAGCGTAAG | CTTACGCTGTATGAAGATTCGTATTCATTAACAAAATTAAGCTTTTGGTTGGGTGAAGAAGTG |
| ProMiZEP-mProbe | CACTTCTTCACCCAACCAAAAGAAAAAAAAAATTAATGAATACGAATCTTCATACAGCGTAAG | CTTACGCTGTATGAAGATTCGTATTCATTAATTTTTTTTTTCTTTTGGTTGGGTGAAGAAGTG |
| ProMiBCH1-Probe | GCTTTTAGCATACAGAAAAGGAACCCAGCTTTGATCAATTGCCTTGGGCC | GGCCCAAGGCAATTGATCAAAGCTGGGTTCCTTTTCTGTATGCTAAAAGC |
| ProMiBCH1-mProbe | GCTTTTAGCATGGGGGGGGGGGGGCCAGCTTTGATCAATTGCCTTGGGCC | GGCCCAAGGCAATTGATCAAAGCTGGCCCCCCCCCCCCCATGCTAAAAGC |
| MiGAIP1-PGEX4T | GATCTGGTTCCGCGTGGATCCATGAAGAGAGAACACAAACAACTCG | AGTCAGTCACGATGCGGCCGCTCAGTGAAACCGAACCGG |
| ProMiAGL5-Probe (MiGAIP1) | TTTCAACAATCAAGAAATGAGAAAGGAAAGAAGAAAAAAGAAAGAAAAAAAAAAAAAGTGTCT | AGACACTTTTTTTTTTTTTCTTTCTTTTTTCTTCTTTCCTTTCTCATTTCTTGATTGTTGAAA |
| ProMiAGL5-mProbe(MiGAIP1) | TTTCAACAATCCCCCCCCCCCCCCCCCCCCCCCCCCCCCCCCCCCCCCCCCCCAAAAGTGTCT | AGACACTTTTGGGGGGGGGGGGGGGGGGGGGGGGGGGGGGGGGGGGGGGGGGGATTGTTGAAA |
| MiPIF5-PGEX4T | GATCTGGTTCCGCGTGGATCCATGAATCCATGTATACCAGATTG | AGTCAGTCACGATGCGGCCGCTTAACCTGTCTTGCCACTTAGAGAG |
| ProMiAGL5-Probe (MiPIF5) | GAGCATCACATGGGAGTCTATTTTCACTTTGTAATTTGAAAAGGAAAGAGATATATAC | GTATATATCTCTTTCCTTTTCAAATTACAAAGTGAAAATAGACTCCCATGTGATGCTC |
| ProMiAGL5-mProbe (MiPIF5) | GAGCATTTTTTTTGAGTCTATTTTCACTTTGTAATTTGAAAAGGAAAGAGATATATAC | GTATATATCTCTTTCCTTTTCAAATTACAAAGTGAAAATAGACTCAAAAAAAATGCTC |
| MiZEP-pCAMBIA1301 | AGAACACGGGGGACTCTTGACATGGAAATGAGTTTGCAGTCTC | GGGGAAATTCGAGCTGGTCACTTAACTTGTTTCAGTAGTGACAGAAGAG |
| MiAGL5-pCAMBIA1301 | AGAACACGGGGGACTCTTGACATGGAGTTTCCAAACCAAGAAC | GGGGAAATTCGAGCTGGTCACTCAATGATTGGGTTCCAGG |
| MiGAIP1-pCAMBIA1301 | AGAACACGGGGGACTCTTGACATGAAGAGAGAACACAAACAACTCG | GGGGAAATTCGAGCTGGTCACTCAGTGAAACCGAACCGG |
| MiPIF5-pCAMBIA1301 | AGAACACGGGGGACTCTTGACATGAATCCATGTATACCAGATTG | GGGGAAATTCGAGCTGGTCACTTAACCTGTCTTGCCACTTAGAGAG |
| MiAGL5-pTRV2 | ATTCTGTGAGTAAGGTTACCGATGATTGGGTTCCAGGAGG | GCCCGGGCCTCGAGACGCGTGGAAAACGAGAATTCTGAACAGC |
| MiGAIP1-pTRV2 | ATTCTGTGAGTAAGGTTACCGCCGTTCTCCTGTGAATCAGC | GCCCGGGCCTCGAGACGCGTGCCGATCCGTCGACGC |
| MiPIF5-pTRV2 | ATTCTGTGAGTAAGGTTACCGCATGACTAGTGCTAGGCGGTG | GCCCGGGCCTCGAGACGCGTGGATCAATCCATGGCTATTGCC |
| MiGAIP1-p2YN | TACGAACGATAGTTAATTAACATGAAGAGAGAACACAAACAACTCG | ACTGCCACCTCCTCCACTAGTGTGAAACCGAACCGG |
| MiGAIP1-p2YC | TACGAACGATAGTTAATTAACATGAAGAGAGAACACAAACAACTCG | ACTGCCACCTCCTCCACTAGTGTGAAACCGAACCGG |
| MiPIF5-p2YN | TACGAACGATAGTTAATTAACATGAATCCATGTATACCAGATTG | ACTGCCACCTCCTCCACTAGTACCTGTCTTGCCACTTAGAGAG |
| MiPIF5-p2YC | TACGAACGATAGTTAATTAACATGAATCCATGTATACCAGATTG | ACTGCCACCTCCTCCACTAGTACCTGTCTTGCCACTTAGAGAG |
| MiCRY1-p2YN | TACGAACGATAGTTAATTAACATGTCAGGTGGTGGGTGTAGTATAG | ACTGCCACCTCCTCCACTAGTCCCAGTTTGAGAAAGCCG |
| MiCRY1-p2YC | TACGAACGATAGTTAATTAACATGTCAGGTGGTGGGTGTAGTATAG | ACTGCCACCTCCTCCACTAGTCCCAGTTTGAGAAAGCCG |
| MiGAIP1-nLUC | ACGGGGGACGAGCTCGGTACCATGAAGAGAGAACACAAACAACTCG | CGCGTACGAGATCTGGTCGACGTGAAACCGAACCGG |
| MiGAIP1-cLUC | TACGCGTCCCGGGGCGGTACCATGAAGAGAGAACACAAACAACTCG | ACGAAAGCTCTGCAGGTCGACTCAGTGAAACCGAACCGG |
| MiPIF5-nLUC | ACGGGGGACGAGCTCGGTACCATGAATCCATGTATACCAGATTG | CGCGTACGAGATCTGGTCGACACCTGTCTTGCCACTTAGAGAG |
| MiPIF5-cLUC | TACGCGTCCCGGGGCGGTACCATGAATCCATGTATACCAGATTG | ACGAAAGCTCTGCAGGTCGACTTAACCTGTCTTGCCACTTAGAGAG |
| MiCRY1-nLUC | ACGGGGGACGAGCTCGGTACCATGTCAGGTGGTGGGTGTAGTATAG | CGCGTACGAGATCTGGTCGACCCCAGTTTGAGAAAGCCG |
| MiCRY1-cLUC | TACGCGTCCCGGGGCGGTACCATGTCAGGTGGTGGGTGTAGTATAG | ACGAAAGCTCTGCAGGTCGACTTACCCAGTTTGAGAAAGCCG |
| MiGAIP1-pCAMBIA1307 | AACTCGGTATCTAGAACTAGTATGAAGAGAGAACACAAACAACTCG | GTCGACGGTATCGATAAGCTTTCAGTGAAACCGAACCGG |
| MiGAIP1-pET32a | GCCATGGCTGATATCGGATCCATGAAGAGAGAACACAAACAACTCG | CTCGAGTGCGGCCGCAAGCTTTCAGTGAAACCGAACCGG |
| MiCRY1-pGEX4T | GATCTGGTTCCGCGTGGATCCATGTCAGGTGGTGGGTGTAGTATAG | AGTCAGTCACGATGCGGCCGCTTACCCAGTTTGAGAAAGCCG |

Table S4 FPKM values of five candidate genes in control and blue light-treated mango flesh

| Gene Name | *MiAGL5* | *MiERF1* | *MiERF61L* | *MiMYB70* | *MiMUTE* |
| --- | --- | --- | --- | --- | --- |
| ID | mango018013 | mango034445 | mango028026 | mango014959 | mango003016 |
| F-0h1_fpkm | 0.74 | 67.08 | 17.61 | 14.61 | 0.81 |
| F-0h2_fpkm | 0.28 | 55.89 | 23.93 | 16.14 | 1.31 |
| F-0h3_fpkm | 0.96 | 43.69 | 20.15 | 15.76 | 0.80 |
| FD-24h1_fpkm | 1.72 | 60.60 | 20.83 | 7.07 | 0.48 |
| FD-24h2_fpkm | 1.09 | 38.63 | 23.45 | 6.29 | 0.99 |
| FD-24h3_fpkm | 1.17 | 74.81 | 24.43 | 9.85 | 2.46 |
| FD-72h1_fpkm | 1.47 | 41.37 | 22.92 | 7.76 | 2.03 |
| FD-72h2_fpkm | 1.58 | 66.03 | 20.47 | 8.77 | 0.86 |
| FD-72h3_fpkm | 1.39 | 49.18 | 25.97 | 7.26 | 1.19 |
| FD-144h1_fpkm | 1.27 | 94.72 | 26.46 | 11.94 | 2.60 |
| FD-144h2_fpkm | 5.89 | 109.11 | 20.79 | 14.27 | 1.51 |
| FD-144h3_fpkm | 4.29 | 123.82 | 21.01 | 12.25 | 1.51 |
| FD-216h1_fpkm | 7.96 | 86.30 | 14.33 | 11.39 | 1.78 |
| FD-216h2_fpkm | 5.49 | 93.50 | 16.13 | 13.43 | 2.65 |
| FD-216h3_fpkm | 7.97 | 107.78 | 17.72 | 11.67 | 1.36 |
| F-0h1_fpkm | 0.74 | 67.08 | 17.61 | 14.61 | 0.81 |
| F-0h2_fpkm | 0.28 | 55.89 | 23.93 | 16.14 | 1.31 |
| F-0h3_fpkm | 0.96 | 43.69 | 20.15 | 15.76 | 0.80 |
| FB-24h1_fpkm | 2.95 | 125.27 | 38.61 | 9.71 | 0.84 |
| FB-24h2_fpkm | 1.05 | 135.02 | 32.97 | 9.75 | 0.57 |
| FB-24h3_fpkm | 2.18 | 115.37 | 44.45 | 13.86 | 1.50 |
| FB-72h1_fpkm | 11.49 | 241.46 | 69.10 | 22.28 | 2.78 |
| FB-72h2_fpkm | 5.55 | 184.76 | 66.03 | 24.39 | 0.86 |
| FB-72h3_fpkm | 3.99 | 162.08 | 36.95 | 10.76 | 0.70 |
| FB-144h1_fpkm | 14.81 | 295.88 | 88.33 | 43.38 | 3.94 |
| FB-144h2_fpkm | 16.12 | 341.01 | 129.76 | 52.04 | 4.58 |
| FB-144h3_fpkm | 11.28 | 328.09 | 72.08 | 30.63 | 3.33 |
| FB-216h1_fpkm | 21.83 | 437.03 | 119.14 | 77.54 | 12.55 |
| FB-216h2_fpkm | 26.25 | 432.95 | 146.56 | 61.19 | 15.59 |
| FB-216h3_fpkm | 21.19 | 492.39 | 88.80 | 65.18 | 16.42 |

Supplemental File

Supplemental File S1 Sequence data for protein

>MiAGL5 (mango018013) [*Mangifera indica*]

MEFPNQEPESSSNKKTVRGKIEIKRIENTTNRQVTFCKRRNGLLKKAYELSVLCEAEVALIVFSSRGRLYEYANNSVRSTIERYKKSCADSSNPGSVTEANAQFYQQEATKLRRQIREIQNLNKHILGEALSSLSFKELKNLEARVEKGITRIRSKKNDMLFAEIELMQKREMQLQNDNMHLQAMIAENENSEQQQSSIMQAAVYDQSVPSQSYHRDFIRVNLLEPNH*

>AtAGL5 (AT2G42830.2) [*Arabidopsis thaliana*]

MEGGASNEVAESSKKIGRGKIEIKRIENTTNRQVTFCKRRNGLLKKAYELSVLCDAEVALVIFSTRGRLYEYANNSVRGTIERYKKACSDAVNPPTITEANTQYYQQEASKLRRQIRDIQNLNRHILGESLGSLNFKELKNLESRLEKGISRVRSKKHEMLVAEIEYMQKRVKEIELQNDNMYLRSKITERTGLQQQESSVIHQGTVYESGVTSSHQSGQYNRNYIAVNLLEPNQNSSNQDQPPLQLV*

> MdAGL1 (NP_001280918.1) [*Malus domestica*]

MEFPNQAPESSSQKKLGRGKIEIKRIENTTNRQVTFCKRRNGLLKKAYELSVLCDAEVALIVFSNRGRLYCsEYANNSVRATIDRYKKAYADPTNSGSVSEANTQFYQQEASKLRRQIREIQNSNRHILGEALSSLNAKELKCsNLEGRLEKGISRIRSKKNEMLFSEIEFMQKRETELQHHNNFLRAKIAENEREEQQHTHMMPGTSYDQSMPCsSHSYDRNFLPAVILESNNNHYPHQVQTALQLV*

>SlTAGL1 (NP_001300859.1) [*Solanum lycopersicum*]

MVFPINQELLVDESSSQLRKTSGGTGGGGRGKIEIKRIENTTNRQVTFCKRRNGLLKKAYELSVLCDAEVCsSLIVFSSRGRLYEYANNSVRATIDRYKKHHADSTSTGSVSEANTQYYQQEASKLRRQIRDIQTYNRQIVGCsEALGSLSPRDLKNLEGKLEKAIGRVRSKKNELLFSEIELMQKREIELQNANMYLRAKIAEVERAQEQMNLCsMPGGGGGGGGGGGGGSDHQYHHQPNYEDARNNFLPVNLLEPNPHYSRRDNGDQTPLQLV*

>CsAGL5 (XP_024956880.1) [*Citrus sinensis*]

MEFPKQNPESSSQSKKIGRGKIEIKRIENTTNRQVTFCKRRNGLLKKAYELSVLCDAEVALIVFSSRGRLCsYEYANNSVRATIDRYKKACADSSNPGSITEANTQFYQQEATKLRRQIREIQNLNRHILGEALSTLNFKELCsKNLEARLEKGIGRVRSKKNEMLLAEIEFMEKREIQLQNDNMYLRARISENERAQQERQSESMMQQGGGHVCsYEPAASQPYDRNFLPVNLLEPNHQYARQDDQPPLQLVIAASTSEICKSLSATLH*

>MiPIF5 (mango026944) [*Mangifera indica*]

MNPCIPDWNFEGDPPVTDQKKPIGRPEHDLVELLWQNGQVVLNSQTYRKPSLNPNEFRQVQKQTLRERGSYGNSSNLIQDDETISWIQYPLEDSFEKDVSNFFSELSPSRMEAGKNTIRFGDEKLVKFGASGVATNSQPTNVTNPVFPGMNRNAMPPPRFEFHDAAQKDKNLGGFGEVVNICQTTAPLKGEQRYSNGQFVHKGTGTMTNGEVRDPSMMTVGSSHCGSNQVAYDLDISRASSNGAGTTGISPGNLNTDVQRVISQSERGKAETLEPTVTSSSGGSGSSFNRTSKQSTGVISHKRKSRDAGESECQSDAAELDSAEGNKSSQKSGSSRRSRAAEVHNLSERRRRDRINEKMRALQELIPHCNKTDKASMLDEAIEYLKSLQLQLQVMWMGSGMAQMMFPGVQHYMSRMGMGMGPTSLPSITSPVHLSRVPLVDQSMAIAQAQNQAVMCQNSMLNPVSFQNQMQNANFSDQCAHYMGFHPMQTTSQPINMFRFGSPTVQSQVTSPPSTSHGPFMAGAATDNTSLSGKTG*

>CsPIF4 (KAH9770529.1) [*Citrus sinensis*]

MNPCIPDWNFEGDIPISNQMKPMGQDNELVELLWQNGHVVLSSQAQTQRKPSLNHNEPRQVQKQTLRGSGSYGNSSNLIQDDETVSWIHCPIEDSFEKDFYSHLFSELPPSGPMEVDKHTRQLREEKMVKFDPPGAVTSSQHPNVNHSVVPELQRNAMPPPRFEVHDAAPQNKNLGDLGKLVNFSQSTAPPKGELGPCSGQFDRKRSGNLTQGEVRECSMMTVGSSHCGSNQVAYDLDMSRASSSGVGTTGLSPGKLNDDVRKVISPSERGKTETIEPTVTSSSGGSGSSFNRTSKQSTGDNSLKRKSRDAVDSECQSEAAGFESGAGNKTAQRSGSCRRSRAAEVHNLSERRRRDRINEKMRALQELIPHCNKTDKASMLDEAIEYLKSLQLQLQVMWMGSGMAPLMFPGMQHYMSRMGMGMGPPPLPSVTNPMHFSRVPLVDQSMSMAQAQNRAVMCQASVLNPVNYQNQMQNSNFTEQYARYMGFHPMQANSQPMNMFRFGSPTMQNQIVSLPSSSCVPFSGGAATDNSPLSGKMD*

>SlPIF4 (NP_001294937.1) [*Solanum lycopersicum*]

MNPYLPEWNIETELPAPHQKKPMGFDHELVELLWRNGEVVLHSQTHKKQPGYDPNECRQFNKHDQPTIRVAGNQTNLIQDDETVAWLNCPIDDSFDKEFCSPFLSDISTNPHLGEEPDKSIRQSEDNNKVFKFDPLEINHVLPQSHHSGFDPNPMPPPRFHNFGSAQQKHHIVGGDQKGVNFPPPIRSSNVQLGGKEARSNLMLQDIKEGSVMTVGSSHCGSNQVDTSRFSSSANRGLSAAMITDYTGKISPQSDTMDRDTFEPANTSSSSGRSGSSYARACNQSTATNSQGHKRKSRDGEEPECQSKADELESAGGNKSAQKSGTARRSRAAEVHNLSERRRRDRINEKMKALQELLPHSTKTDKASMLDEAIEYLKSLQMQLQMMWMGSGMASMMFPGVQHYISRMGMGMGPPSVPSMHNAMHLARLPLVDPAIPLTQAAPNNQAAAMCQNSMLNQVNYQRHLQNPNFPDQYASYMGFHPLQGASQPINIFGLGSHTAQQTQQLPHPTNSNAPAT*

>AtPIF5 (AT3G59060.4) [*Arabidopsis thaliana*]

MEQVFADWNFEDNFHMSTNKRSIRPEDELVELLWRDGQVVLQSQARREPSVQVQTHKQETLRKPNNIFLDNQETVQKPNYAALDDQETVSWIQYPPDDVIDPFESEFSSHFFSSIDHLGGPEKPRTIEETVKHEAQAMAPPKFRSSVITVGPSHCGSNQSTNIHQATTLPVSMSDRSKNVEERLDTSSGGSSGCSYGRNNKETVSGTSVTIDRKRKHVMDADQESVSQSDIGLTSTDDQTMGNKSSQRSGSTRRSRAAEVHNLSERRRRDRINERMKALQELIPHCSRTDKASILDEAIDYLKSLQMQLQVMWMGSGMAAAAAAAASPMMFPGVQSSPYINQMAMQSQMQLSQFPVMNRSAPQNHPGLVCQNPVQLQLQAQNQILSEQLARYMGGIPQMPPAGNQMQTVQQQPADMLGFGSPAGPQSQLSAPATTDSLHMGKIG*

>CsPIF5 (XP_010504884.1) [*Camelina sativa*]

MEQVFADWNFEDNFHMSTNKRSIRPEDELEELLWRDGQVVLQSQARREPSVQLQSHKQDTLRKPCNIFLDDQEIVPKPNNNALLDDQETVSWIQYPPDDVVDPFESEFSSHLFSTVNHLGSPEKPRSVEETVKNEAQAMAPPKFRSSVITVGPSHCGRNQSTNDHQVTTLPVSMTDGSKNVEERLDTSSGGSSGCSYGRNNKETVSGRSVTIDRKRKHVMDADQESVSQSDVPLTSTDDIQVTGNKSSQRSGSTRRSRAAEVHNLSERRRRDRINERMKALQELIPHCSKTDKASILDEAIDYLKSLQMQLQVMWMGSGMAAAAAAAAATTPMMFPGVQSSPYINQMAMQSQMQLPQFPVMNRSAPQNHPGLVCQNPVQLQLQAQNQILSEQLARYMGGFPQMPPAANQAVQQQQQPTDMMRFGSPAGPQSQLSAPASTDSLRMGKIG*

>CrPIF5 (XP_006291151.1) [*Capsella rubella*]

MEQVFADWNFEDNFHMSTNKRSIRPEDELVELLWRDGQVVLQSQARREPSVQVQNHKQALRKPHNIFLDNQETVQKPNNALLDDQETVSWIQYPPDDVVDPFESEFSSHLFSSINHLDGPEKPRSIEETVKSEAQAMAPPKFRSSVITVGPSHCGSNQSTNDHQVNTLPVSMTDRSKNVEERLDTSSGGSSGCSYGRNNKETVSGRSVTIDRKRKHVMDADQESVSQSDVRLTSTDDHQVTGNKSSQRSGSTRRSRAAEVHNLSERRRRDRINERMKALQELIPHCSKTDKASILDEAIDYLKSLQMQLQVMWMGSGMAAAAAAAATTPMMFPGVQPSPYMNQMAMQSQMQMPQFPVMNRSAPQNHPGLVCQNPVQLQLQAQNQMLSDQLARYMGGFPQMPSAANQAVQQPTTDMMKFGSPAGPQSQLSAPATTDSLRMG*

>MiGAIP1 (mango014171) [*Mangifera indica*]

MKREHKQLDPPANSASDSGKAKMWEDDVAAAADGGMDELLAVLGYHVKSSDMAEVAQKLEQLEEAMCSGDGFSQLASDSVHYDPSNLSTWLEGMLSEFSPDPASDPSTLTSIDFPNQQRIFEGACTSDYDLKAIPGKAMYNDNTTPASSSVSTARDHKRLKTELYSPESTRPVILADSQENGIRLVHTLMACAEAVQQNNLPIAEAFVKKIRFLAISQAGAMGKVATCFADAIAGRIYGHYPENPIDHNFAQLLEMHFYEACPYLKFAHFTANQAILEAFEGKKRVHVIDFSMNQGLQWPALMQALALRPGGAPKFRLTGIGPPAPDNSDHLQEVGWKLAQLAETIQVQFEYRGFVAKSLADLDASMLDLRPSEVDSVAVNSVFELHRLLARPGAIDKVLSVVKNVKPDIFTIVEQEANHNGRIFLDRFTEALHYYSTMFDSLEGSVSGPEKAMSELYLQKQIYDVVAYEGVNRIERHENLAQWRTRLGSSGFLPVHLGSNAFKQASMLLALFAGGEGYRVEENNGSLMLGWYTRPLIATSAWKLANKPVRFH*

>AtRGA1 (AT2G01570.1) [*Arabidopsis thaliana*]

MKRDHHQFQGRLSNHGTSSSSSSISKDKMMMVKKEEDGGGNMDDELLAVLGYKVRSSEMAEVALKLEQLETMMSNVQEDGLSHLATDTVHYNPSELYSWLDNMLSELNPPPLPASSNGLDPVLPSPEICGFPASDYDLKVIPGNAIYQFPAIDSSSSSNNQNKRLKSCSSPDSMVTSTSTGTQIGGVIGTTVTTTTTTTTAAGESTRSVILVDSQENGVRLVHALMACAEAIQQNNLTLAEALVKQIGCLAVSQAGAMRKVATYFAEALARRIYRLSPPQNQIDHCLSDTLQMHFYETCPYLKFAHFTANQAILEAFEGKKRVHVIDFSMNQGLQWPALMQALALREGGPPTFRLTGIGPPAPDNSDHLHEVGCKLAQLAEAIHVEFEYRGFVANSLADLDASMLELRPSDTEAVAVNSVFELHKLLGRPGGIEKVLGVVKQIKPVIFTVVEQESNHNGPVFLDRFTESLHYYSTLFDSLEGVPNSQDKVMSEVYLGKQICNLVACEGPDRVERHETLSQWGNRFGSSGLAPAHLGSNAFKQASMLLSVFNSGQGYRVEESNGCLMLGWHTRPLITTSAWKLSTAAY*

>CsGAI (KAH9794669.1) [*Citrus sinensis*]

MKREHSQLDPPPPPPSCLAGPSGKGKMWEEEQTDGGGMDELLAVLGYNVRSSDMVEVAQKIEQLEEAMGAFQADGINHLATDTVHYDPSNLCTWLESMLTEFNPTMPAGLGLDPVPAPSVFDDSSFVAPAESSTITSLDFSHQRVLEEPSTSDYDLKAIPGKAMYGANNSQNSSNNYLSLSSASASSLSSSTTTRENKRLKTSEFYPPESTRPIVLADSQENGIRLVHALMACAEAVQQNNLTLAEAFVKQIRFLAVSQAGAMGKVATHFAEALARRIYGLYPQSPIDHSFSELLEMQFYETCPYLKFAHFTANQAILEAFDGKRRVHVIDFSMNQGMQWPALMQALALRPGGPPAFRLTGIGPPAADNTDQLQQVGWKLAQLAESIHVEFEYRGFVANSLADLDASMLEIKPSEVDSVAVNSVFELHKLLAQPGAIDKVLSVVENIKPDIFTVAEQEANHNSPVFLDRFTESLHYYSTMFDSLEGSVNSPENHKAMTEAYLGNQICNVVACEGADRVERHETLAQWRTRFGSAGFIPAHIGSNAYKQASMLLALFAGGDGYKVEENNGCLTLGWYTRPLIATSAWKLAAKR*

>CrRGA (XP_023638148.1) [*Capsella rubella*]

MKRDLHQYQGPNATHGTCSSSSPLPPLSKDKMMMVKEEEDGNNMDELLAVLGYKVRSSEMAEVALKLEQLETMMGNVQEDGLSHLATDTVHYNPSELYSWLDNMLTELNPPPPPPPPSSLDPILIDNNSFLCTAGDSSTITSIGGGFPASDYDLKVIPGNAIYQQRSQFAIDSSSSSSNQNKRLKSCSSPDSMVTSTSTTAMQIGGVIGTTVTTTTTTAAAAAESTRSVILVDSQENGVRLVHALMACAEAVQQNNLTLAEALVKQIGCLAVSQSGAMRKVATYFAEALARRIYRLSPPQNQIDHSLSDTLQMHFYETCPYLKFAHFTANQAILEAFQGKKRVHVIDFSMNQGLQWPALMQALALREGGPPVFRLTGIGPPAPDNSDHLHEVGCKLAQLAEAIHVEFEYRGFVANSLADLDASMLELRPSEVESVAVNSVFELHKLLGRPGGIEKVLGVVKQIKPVIFTVVEQESNHNGPVFLDRFTESLHYYSTLFDSLEGVPSSQDKVMSEVYLGKQICNLVACEGPDRVERHETLSQWGNRFGSSGFAPAHLGSNAFKQASMLLSVFNSGQGYRVEENNGCLMLGWHTRPLITTSAWKPSAAH*

>GmGAI1 (NP_001240948.1 ) [*Glycine max*]

MKRERQQLGSNAGTSSCGYSSGKSNLWEEEGGMDELLAVVGYKVRSSDMAEVAQKLERLEEAMGNVQDDLTDLSNDAVHYNPSDISNWLQTMLSNFDPLPSEEPEKDSASSDYDLKAIPGKAIYGGGSDALPNPKRVRTDESTRAVVVVDLQENGIRLVHSLMACAEAVENNNLAVAEALVKQIGFLALSQVGAMRKVATYFAEALARRIYRVFPQQHSLSDSLQIHFYETCPYLKFAHFTANQAILEAFQGKNRVHVIDFGINQGMQWPALMQALALRNDGPPVFRLTGIGPPAADNSDHLQEVGWKLAQLAERIHVQFEYRGFVANSLADLDASMLDLREDESVAVNSVFEFHKLLARPGAVEKVLSVVRQIRPEILTVVEQEANHNGLSFVDRFTESLHYYSTLFDSLEGSPVNPNDKAMSEVYLGKQICNVVACEGMDRVERHETLNQWRNRFGSTGFSPVHLGSNAYKQASMLLSLFGGGDGYRVEENNGCLMLGWHTRPLIATSVWQLATKSVVAAH*
